# Quantitative Assessment of a Novel Device Designed for Patient-centric Sampling of Dried Plasma Using Targeted Proteomics

**DOI:** 10.1021/acs.analchem.4c05455

**Published:** 2025-06-18

**Authors:** Andreas Hober, Marcus Henricsson, Tim Ruckh, Pia Davidsson, Benjamin Challis, Tasso Miliotis

**Affiliations:** 1 Translational Science & Experimental Medicine, Research and Early Development, Cardiovascular, Renal and Metabolism (CVRM), BioPharmaceuticals R&D, AstraZeneca, 431 83 Gothenburg, Sweden; 2 R&D Digital Health, AstraZeneca, 431 83 Gothenburg, Sweden; 3 Translational Science & Experimental Medicine, Research and Early Development, Cardiovascular, Renal and Metabolism (CVRM), BioPharmaceuticals R&D, AstraZeneca, Cambridge CB2 0AA, U.K.

## Abstract

Technological advancements have significantly enhanced proteomics by improving the quality, depth, and timeliness of proteomic data, making mass spectrometry a promising tool for personalized medicine. To enable successful longitudinal population-level monitoring, it is essential to have cost-efficient and convenient sample collection methods that yield reliable results. Microsampling performed by patients in their own homes fulfills this requirement. In this study, we evaluate a novel microsampling device that readily prepares a dried plasma sample from blood collected through a finger prick. The device was assessed quantitatively through a targeted proteomics approach, using a selection of stable isotope-labeled protein fragments as standards for plasma proteins spanning a range from 1300 μM to 30 nM. All samples were analyzed using selected reaction monitoring to ensure quantitative robustness. The device was assessed both from a storage perspective and across a group of healthy donors to ensure reliable performance between individuals. The dried plasma obtained from the device shows an excellent quantitative correlation with conventional plasma (*R* > 0.99) and high quantitative precision, with a coefficient of variance (CV) below 10% for 80% of all peptides quantified in the group of healthy donors. All of the targets that perform well in the microsampling device also show good long-term stability when stored at room temperature for up to 232 days, further showcasing the potential benefits of collecting samples in a dry format.

## Graphical abstract

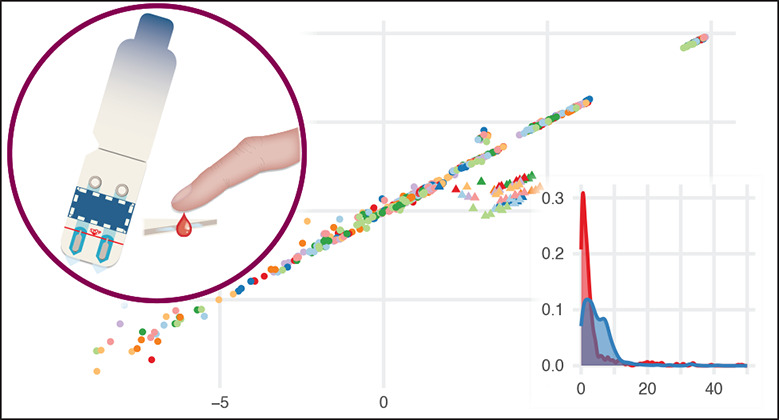

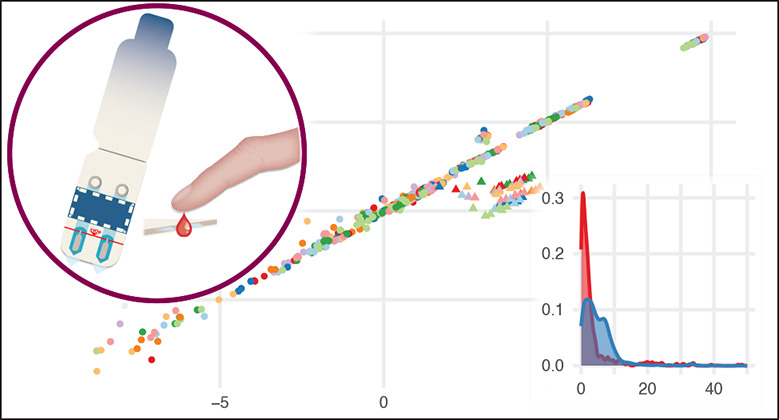

## Introduction

The consequences of the healthcare restrictions due to the coronavirus disease 2019 (COVID-19) pandemic had severe implications on healthcare services that were partially or entirely disrupted in many countries. It also impacted clinical trials, particularly site opening, trial activation, and participant recruitment.^1,2^ The pharmaceutical industry has been working on the decentralization of clinical trials long before the COVID-19 pandemic.^3–5^ During the past decade, home sampling utilizing dried sample matrix spot approaches has gained significant traction by pharmaceutical companies, where various dried blood spots (DBS), as well as dried urine methods, have been implemented in clinical trials.^6–9^ Nevertheless, the recent global pandemic has had a catalytic effect on allocating more resources regarding patient-centric microsampling. The driving forces for using home sampling in clinical studies have been to use technology that retains more patients as active participants in clinical trials.

The utilization of finger pricking and collection of 30–60 μL of capillary blood instead of drawing several milliliters of venous blood when visiting a phlebotomist in a point-of-care facility will decrease patient burden. In fact, the necessity for centrifugation of blood samples and the requirement of cold-chain storage handling remain a substantial obstacle in bringing the sampling event closer to the patient to enable self-sampling or sampling by nonprofessionals. Moreover, dry ice transportation is typically required for conventional liquid samples (e.g., blood, plasma, urine), and international shipment is subjected to strict regulations that add significantly to costs and timelines.^10,11^ In contrast, the shipment of dried samples is drastically less complex, as simple envelopes can be sent by standard mail or courier services. It has also been shown that proteins stored as dried blood or plasma on paper are stable for at least 14 days when stored at room temperature^12,13^ without the need for refrigeration or cold chain storage. Björkesten et al. established that the drying process was reproducible and barely influenced the detection of blood proteins.^14^ They concluded that the detection of some proteins was not significantly affected even following storage for 30 years if the dried blood samples had been stored at either +4 or −24 °C.

For the longitudinal monitoring of a larger population to be successful, sample collection must be cost-efficient and convenient for the patient while producing reliable results. Microsampling can meet these requirements by circumventing traditional venipuncture sampling and cold-chain transportation. This approach also promotes carbon emission reduction and increases trial accessibility, which can facilitate participation by previously underserved populations.^15^

Microsampling using DBS techniques has several limitations, such as variable spot sizes and spot homogeneity, resulting in inconsistent concentration results due to variability in hematocrit levels.^16–18^ Another disadvantage of DBS sampling, from an analytical perspective, is that the sample is contaminated with variable amounts of intracellular components originating from leukocytes and erythrocytes. The presence of these components may cause matrix interferences as well as ionization suppression in mass-spectrometry-based assays, which may result in lower detection sensitivity. These complexities lead to measurement variability from DBS samples, but they may be mitigated by generating dried plasma spots (DPS). Today, blood plasma is a well-established matrix in laboratory medicine. Thus, DPS is a promising microsampling technique, and the dried plasma matrix is expected to lead to the same quantitative result that neat plasma would for an analytical measurement.

The advantages of collecting, drying and transporting small, dried samples have supported quantitative assay development for therapeutic drug monitoring.^19–23^ The first-generation DPS cards utilized either vertical^22^ or lateral^24,25^ flow separation membranes (e.g., Whatman CF12, Ahlstrom 226, or Munktell TFN) to produce cell-free plasma. However, both of these formats are associated with challenges, including variable sample output related to hematocrit values.^26–28^ It has also been shown that lateral flow devices have unrestricted plasma flow across an undefined collection area, resulting in a concentration gradient of analytes due to chromatographic effects.^25^ These quantitative sampling shortcomings have been addressed by commercial manufacturers and academic groups by implementing volumetric control of the sampling, circumventing these issues to enable precise and reproducible sampling.^29–33^

In this study, we evaluated a novel DPS microsampling device, Capitainer Plasma, from Capitainer AB (Stockholm, Sweden), a prototype of a product that will be launched at the end of 2024 with the name Capitainer SEP10. The device prepares a volume-defined dried plasma sample from a fingerprick capillary blood sample with a reported volume precision below 3% (coefficient of variance (CV)).^34^ Briefly, the DPS card is based on passive filtration and utilizes an integrated metering channel for sampling a precise and consistent plasma volume, which is stored in a precut paper disk. We assessed the device quantitatively through a targeted proteomics approach. We used a selection of stable isotope-labeled protein fragments as standards for plasma proteins ranging from 1300 μM to 30 nM. Each standard corresponds to one single target protein fragment and releases its peptides upon digestion, accounting for potential digestion kinetic effects. This allows for a more robust quantification that enables the use of multiple peptides per protein target.^35,36^ All samples were analyzed using selected reaction monitoring, and to ensure quantitative robustness, the device was assessed both from a storage perspective and across a group of healthy donors to ensure reliable performance between individuals.

## Methods

### Sample Collection

All samples were collected from healthy volunteers with informed consent of all donors. The study was performed in accordance with the local ethical regulations (Regionala etikprövningsnämnden, Dnr: 033-10).

### Method Development for MS Analysis

Stable isotope-labeled recombinant protein fragments (qRePS, ProteomEdge, Stockholm, Sweden) were reduced, alkylated, and digested with trypsin (SoLu-trypsin, Sigma-Aldrich, Saint Louis, MO, USA). Briefly, 50 pmol of qRePS was diluted in 1× PBS (Gibco) and 10 mM TCEP (Bond-Breaker TCEP Solution, Thermo Fisher Scientific) to a total volume of 40 μL and incubated in a ThermoMixer (Eppendorf, Hamburg, Germany) at 60 °C and 850 rpm for 30 min. All standards were subsequently alkylated by the addition of 10 μL of 200 mM 2-chloroacetamide (CAA) to a final concentration of 40 mM and incubated at room temperature (RT) for 30 min. One microgram of trypsin was added to each standard, and the standards were digested overnight in a ThermoMixer at 37 °C and 850 rpm. The digestions were quenched by the addition of 10 μL of 10% formic acid (FA). Approximately 6 pmol of each digested standard (Table S1) was individually loaded onto the LC system. All targets were screened for any +1 and +2 product ions (b- and y-ions) from their corresponding proteotypic +2 and +3 precursor ions (5–25 amino acids). A maximum of 10 transitions were kept per precursor ion and subjected to collision energy optimization. The final transition list and the corresponding collision energies can be seen in Table S2. All method development was performed using a Thermo TSQ Altis instrument (Thermo Fisher Scientific, Waltham, MA, USA) connected to a Vanquish Horizon LC system (Thermo Thermo Fisher Scientific). The LC system was equipped with a 150 mm Acclaim VANQUISH C18 column (P/N 071399-V, Thermo Fisher Scientific) and operated with an LC gradient as specified in Table S3.

### Elution Optimization

Venous anticoagulated blood (K_2_EDTA) was collected from two individuals and used to prepare two sets of samples. Each set consisted of 18 and 9 samples, respectively, prepared by aspirating the blood using a microsampling device (Capitainer Plasma, Capitainer, Stockholm, Sweden). The remaining blood was prepared into plasma by centrifugation at 2,000 rcf for 10 min. The neat plasma was aliquoted and stored at −80 °C. All dry samples were allowed to dry fully for 24 h at room temperature (RT) before being stored at −20 °C.

The elution optimization was performed in two rounds (Figure 1A), and all conditions were prepared in triplicates. In the first optimization round, six different elution buffers were used: (i) 1× PBS, (ii) 1× PBS, 10 mM TCEP, 1% sodium deoxycholate (SDC), (iii) 1× PBS, 10 mM TCEP, 0.1% RapiGest, (iv) 50 mM ammonium bicarbonate (AmBic), (v) 50 mM AmBic, 10 mM TCEP, 1% SDC, and (vi) 50 mM AmBic, 10 mM TCEP, 0.1% RapiGest. The discs were loosened from the microsampling device using tweezers and incubated in 400 μL of elution buffer on a ThermoMixer for 1 h at 60 °C and 850 rpm in a LoBind 2.0 mL deep well plate (Eppendorf). The second optimization round was performed with three different elution buffers: (ii) 1× PBS, 10 mM TCEP, 1% SDC, (v) 50 mM AmBic, 10 mM TCEP, 1% SDC, and (vii) 50 mM Tris, 10 mM TCEP, 1% SDC. For all dry samples, corresponding plasma samples were prepared by diluting plasma 40 times in the corresponding buffer.

**Figure 1:**
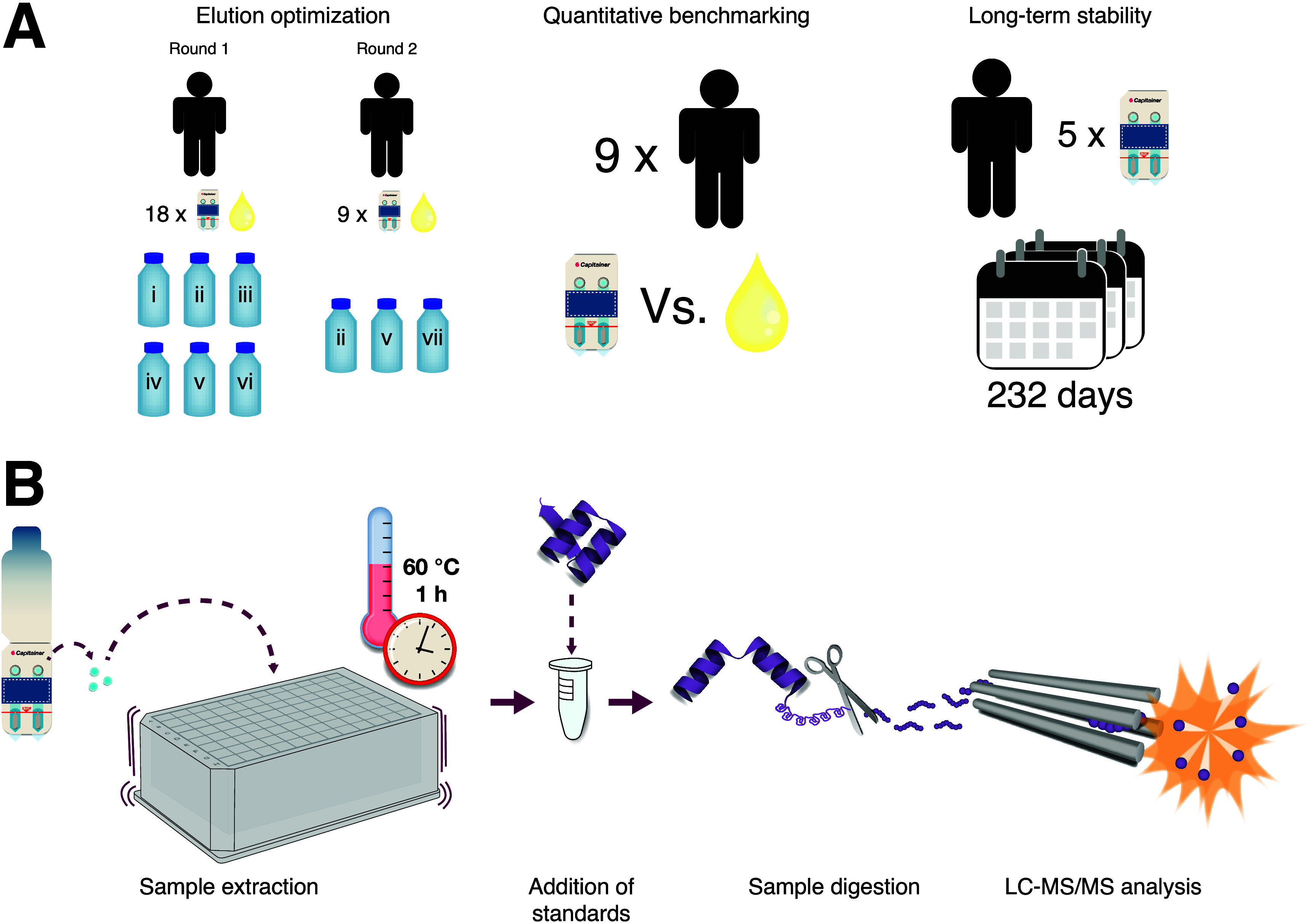
(A) Study design for the three main experiments of the quantitative assessment of the microsampling device. (B) Workflow for sample preparation of samples collected by using the microsampling device.

Forty microliters of each eluted sample (equivalent to 1 μL of neat plasma) was transferred into a LoBind microtiter plate (twin.tec PCR plate, Eppendorf), spiked with 4.79 μL of a pretiterd mixture of qRePS (Table S1) and incubated in a ThermoMixer at 60 °C and 850 rpm for 30 min to reduce the samples. The samples were subsequently alkylated by adding 10 μL of 200 mM CAA to a final concentration of 40 mM and incubated for 30 min in darkness at RT. One microgram of trypsin (10 μL) was added to each sample, and the samples were incubated at 37 °C, 850 rpm, overnight in a ThermoMixer. The digestion was quenched by the addition of 10 μL 10% FA, and the samples were desalted using the EasyPep 96 Sample Prep Kit (Thermo Fisher Scientific).

### Sample Collection for Quantitative Benchmarking

For quantitative benchmarking, samples of paired fingerprick and venous blood were collected from nine healthy donors (Figure 1A). The fingerprick samples were allowed to dry for 24 h at RT before being stored at −20 °C, awaiting analysis. The venous blood (K_2_EDTA) samples were prepared into plasma, as described above, and stored at −80 °C, pending analysis. The samples were digested, as per the description below, in duplicates. Additionally, prepared plasma was loaded in a microsampling device and digested as described below without the addition of any internal standards.

### Sample Collection for Long-Term Stability Evaluation

To assess the long-term quantitative stability of the devices, venous blood was collected from a healthy donor and aspirated with microsampling devices in five duplicates (Figure 1A). The samples were stored for 1 day, 8 days, 21 days, 57 days, or 232 days at RT before being moved to −20 °C, where they were stored until day 239. All samples were subsequently prepared as outlined below and analyzed by LC–MS/MS.

### Sample Preparation for Quantitative Benchmarking and Long-Term Stability Evaluation

Dried samples were allowed to equilibrate at RT before the sample discs were loosened from the microsampling device by using tweezers. The discs were placed in a 2.0 mL microtiter plate and incubated in 400 μL of the established elution buffer (50 mM AmBic, 10 mM TCEP, 1% SDC) for 1 h in a ThermoMixer at 60 °C and 850 rpm. Meanwhile, plasma was diluted 40× in the elution buffer. Forty microliters of diluted plasma or dried sample eluate was spiked with 4.79 μL of a pretiterd mixture of qRePS (Table S1) and incubated for 30 min at 60 °C, 850 rpm in a ThermoMixer. Ten microliters of 200 mM CAA was added to a final concentration of 40 mM, and the samples were incubated in darkness for 30 min at RT. Trypsin was added to the samples (10 μL, 1 μg), and the samples were incubated in a ThermoMixer at 37 °C, 850 rpm overnight. The digestion was quenched by the addition of 10 μL of 10% FA. The samples were desalted using a positive pressure system (Biotage Extrahera LV200, Uppsala, Sweden) and the iST-PSI kit (PreOmics, Planegg, Germany) according to manufacturer instructions to facilitate a high-throughput workflow in a plate-centric manner.

### LC–MS/MS Analysis of Samples for Quantitative Benchmarking and Long-Term Stability

All samples from the nine donors and stability experiment were analyzed by loading approximately 10 μg of peptides onto a Thermo Vanquish NEO (Thermo Fisher Scientific) LC system coupled to a Thermo TSQ Altis (Thermo Fisher Scientific). The LC system was operated in microflow mode and equipped with a 5 mm trapping column (YMC-Triart C18, P/N TA12S03-E5H0 AU, YMC CO., Ltd., Kyoto, Japan), a 150 mm analytical column (YMC-Triart C18, P/N TA12SP9-15H0 AU, YMC CO., Ltd.) and a stainless-steel spray needle (PepSep emitter, P/N PSFSELJ20, Bruker Daltonics, Bremen, Germany). The mobile phase of the LC system consisted of solvent A (0.1% FA) and solvent B (acetonitrile and 0.1% FA). The samples were analyzed using the LC gradient specified in Table S3.

### Data Analysis

All data was processed in Skyline (v 24.1.0.199).^37^ A standardized Peptide Ratio Results report with additional data for the Library Dot Product and Dot Product Light to Heavy was exported for subsequent analysis. All downstream data analysis was performed using R (v 4.1.0).

### Normalization to Albumin

For each sample, the peptide ratios were normalized by a conversion factor established by dividing the absolute quantification of albumin in plasma by the absolute quantification of albumin in the corresponding microsampling device.

### Data Availability

All raw data and extracted ion chromatograms, as well as all exported reports used for the subsequent analysis, can be accessed at Panorama (https://panoramaweb.org/quantitative-benchmarking.url).^38^ Additionally, all raw files are made available through ProteomXchange (PXD056596).^39^

## Results

### Method Development for MS Analysis

The main focus of this study was to assess the quantitative performance of the microsampling device when it was subjected to LC–MS/MS analysis in a targeted manner. To ensure that the evaluation covered proteins of different concentrations in blood plasma, 26 protein targets (two standards targeting different regions of *LPA*) were selected, covering a quantitative range from 1300 pmol/μL to below 10 fmol/μL (Table S1).^40^

### Elution Optimization

Three different buffers and two different detergents were evaluated to assess which elution conditions resulted in the most efficient elution. All samples were processed by using a standardized workflow, as illustrated in Figure 1B. A volume of 40 μL, corresponding to 1 μL of neat plasma, was subjected to digestion. All samples were spiked with internal standards that were digested together with the samples. The elution efficiency was assessed based on the recovery of each target protein to ensure no bias toward any of the selected target proteins. The results remained consistent between samples prepared with SDC and RapiGest, and a good correlation was maintained between the plasma samples and microsampling samples in both instances. As SDC remained a more cost-effective option, it was selected for the final elution conditions.

Three buffers were compared (1× PBS, 50 mM AmBic, and 50 mM Tris) in the second round of the elution optimization to verify the findings from round one as well as assess Tris as an elution buffer. The results are visualized in Figure 2A, where all ratios to standards have been mean-normalized in a peptide-centric manner for all three conditions for visualization purposes. The results show a consistent improvement in sample recovery when AmBic was used as the elution buffer. To ensure that the linearity of the assay was maintained, all peptide ratios for the different conditions were plotted against the peptide ratios obtained in plasma. As can be seen in Figure 2B, the correlation between the plasma samples and the microsampled samples is maintained irrespective of which buffer is used (*R* ^2^ = 0.998). Due to consistent quantitative differences observed between replicates, attributed to differences in input material, all data were normalized to albumin (Figure S1). After normalization, a quantitative outlier can still be observed in the fibrinogen beta chain (*FGB*).

**Figure 2:**
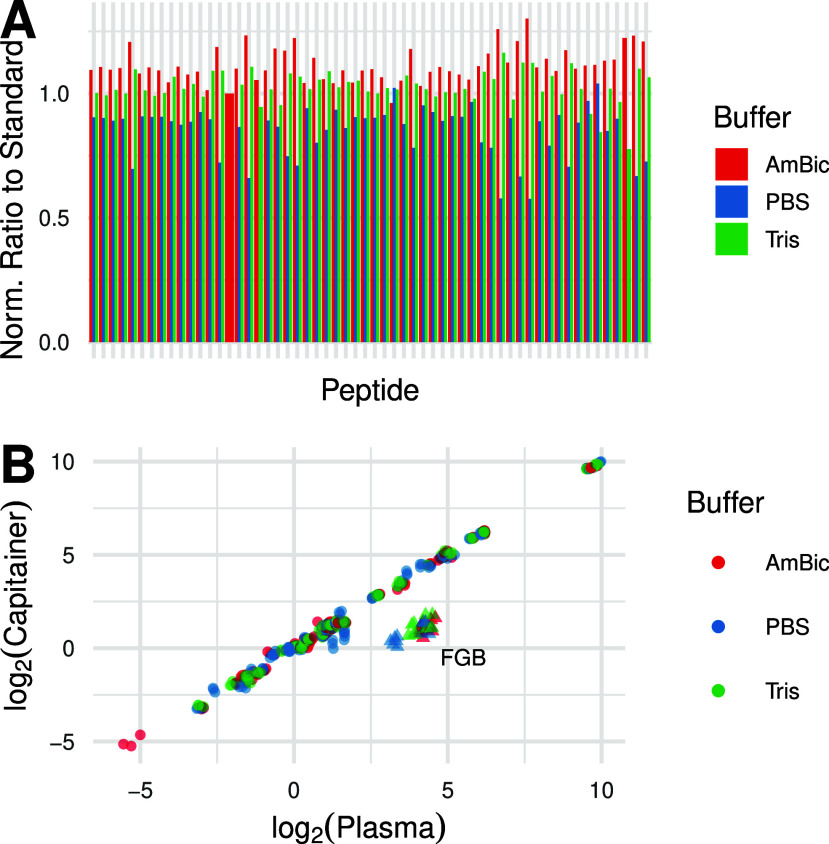
(A) The recovery from the elution evaluation is visualized for the three different buffers evaluated when combined with SDC. There is a higher recovery for the vast majority of the evaluated peptides when using AmBic. (B) Despite the differences in recovery between the three buffers, the quantification after normalization maintains a high correlation between the microsampling device and plasma, irrespective of the buffer. “Capitainer” on the *y*-axis corresponds to the ratio to standard observed in the microsampling devices, whereas “Plasma” on the *x*-axis refers to the ratio to standard observed in the neat plasma samples.

### Quantitative Benchmarking

To assess the reproducibility of the correlation observed in the elution optimization, paired plasma and microsampling samples were collected from nine healthy donors in duplicates. The samples were eluted using the protocol established in the previous experiments, and either 40 μL of eluate or 1 μL of neat plasma was spiked with internal standards. The samples were digested and analyzed by LC–MS/MS. To ensure that the quantitative comparison would not be directly impacted by data with low signal-to-noise ratio, all included peptides had to be quantified in all samples. This resulted in the exclusion of six protein targets. All microsampling samples were normalized to albumin.

As seen in the optimization of the elution protocol, the correlation between the microsampling samples and the plasma is observed across all healthy donors sampled and remains consistent (Figure 3A) with an *R* ^2^ of 0.99. We still observe an outlier cluster consisting of the peptides from *FGB*. The precision of the measurements remains stable, and the majority of peptides, in both the plasma samples and microsampling samples, have a CV below 10% (Figure 3B). All protein targets quantified have been visualized as boxplots for both neat plasma and the microsampling device and are presented in Figure S2. The median CV values were 1.6% and 4.2% for microsampled samples and neat plasma, respectively. Figure 3C and Figure 3D illustrate the molar difference observed between the plasma samples and the microsampling samples at a peptide and protein level in percent after normalization, using the absolute quantification in neat plasma as the reference value. It is noteworthy that the peptides with the most discernible difference observed are the peptides from the *FGB* cluster and the peptides with the lowest molar amount. However, the majority of the targets exhibit minimal difference between the two sampling strategies, with a median difference of −1.8% at a peptide level. Seventeen of the 20 quantifiable targets exhibited a difference below 20%, compared to neat plasma at a protein level, with a median difference of −1.6%.

**Figure 3:**
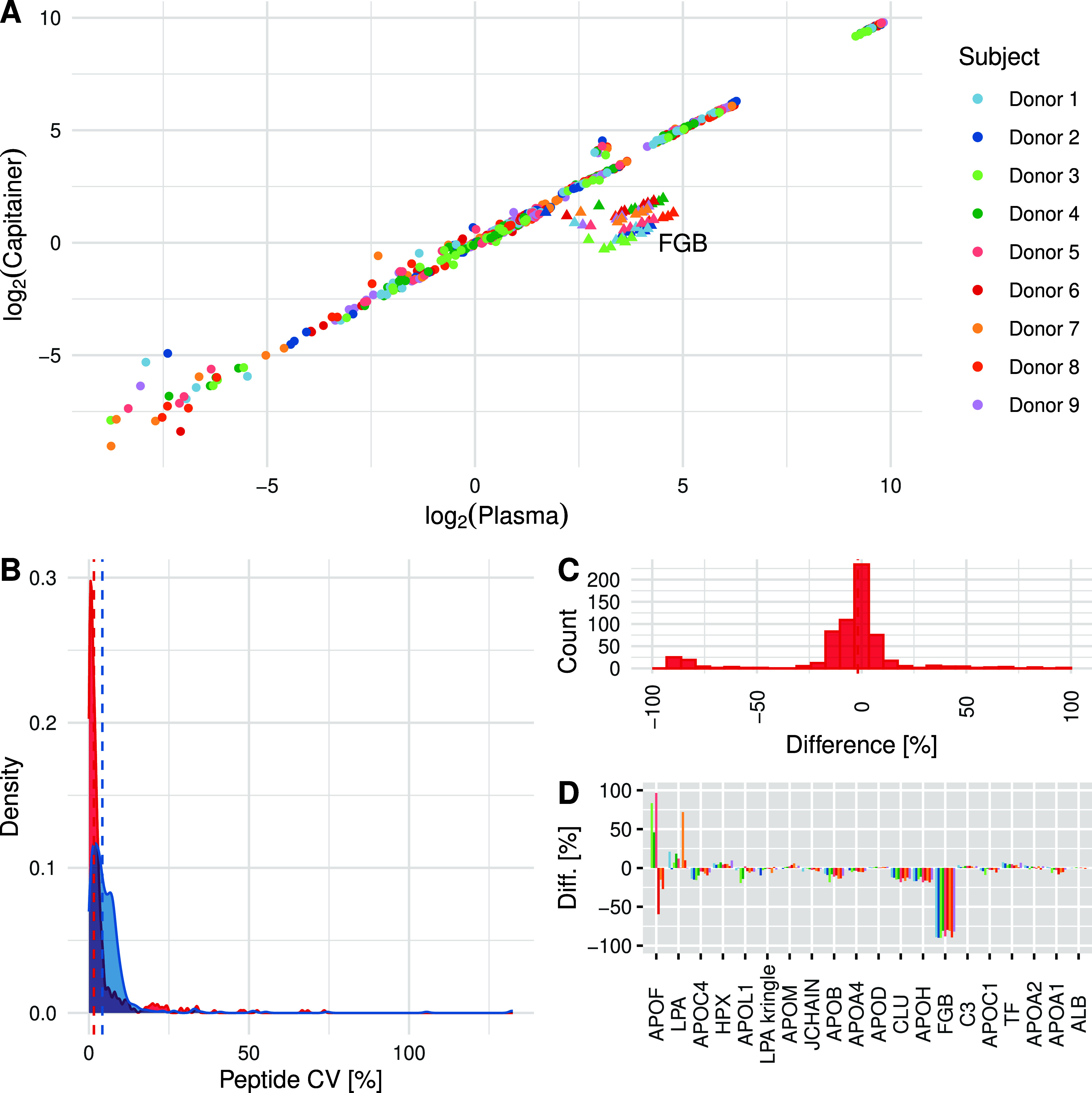
(A) A scatterplot of all peptides quantified in the nine healthy donors, showing a strong correlation. The peptides are plotted with the normalized log_2_(ratio to standard) from the microsampling device on the *y*-axis and the normalized log_2_(ratio to standard) from the plasma on the *x*-axis. (B) The precision of the measurements in both the microsampling device (red) and plasma (blue) is visualized in a density plot. The median CV is highlighted with a dashed line for each subset. (C) The quantitative difference between neat plasma and the microsampling device is small for the vast majority of the quantified peptides, with the highest histogram bin being centered above 0. (D) The quantitative difference at the protein level between the microsampling device and neat plasma is represented by a bar for each donor. The most significant difference is observed in the two least abundant proteins (APOF and LPA) and *FGB*.

To further explore the reason for the outlier cluster, we sought to assess whether this is caused by the quick preparation in an EDTA-free environment of the DPS compared to the conventional centrifugation-based workflow or if it is caused by the device itself. To do this assessment, the Capitainer Plasma card was loaded with plasma rather than blood. These samples were prepared as before but were analyzed with label-free SRM-based quantification. The data were normalized to albumin, which was assigned as a global standard in Skyline. The results can be seen in Figure 4, which shows that there is both a decrease and a higher variance in the *FGB* peptide levels of the plasma loaded in the microsampling device compared to the neat plasma (Figure 4A). The same quantitative offset observed for *FGB* in the capillary blood and the venous blood prepared in the microsampling device compared to neat plasma (Figure 2B and Figure 3A) is observed in the neat plasma processed through the device, while the other proteins still show a good quantitative correlation with the neat plasma sample (Figure 4B). This indicates that processing of the sample in the device specifically impacts *FGB*.

**Figure 4:**
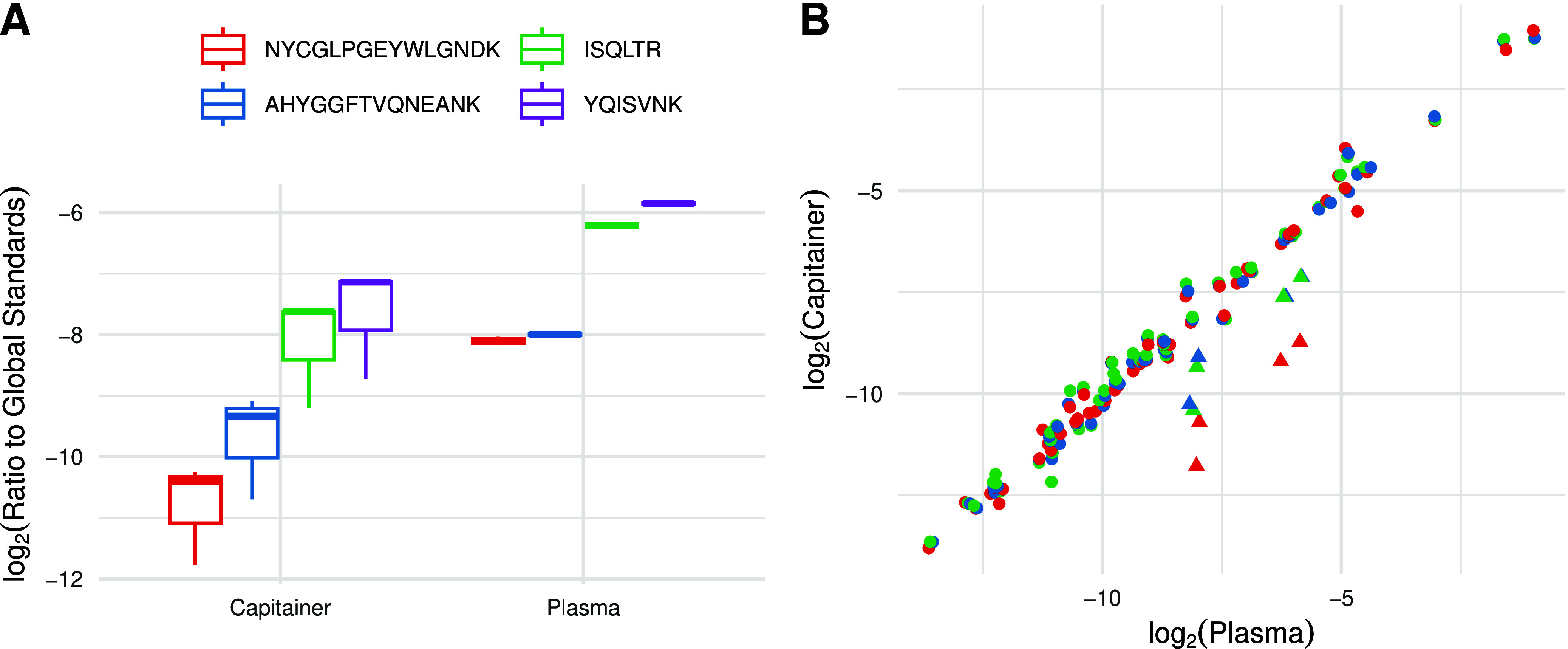
(A) By loading plasma onto the microsampling device, an increase in variance and a decrease in peptide amounts for *FGB* are observed. (B) Despite the impact of the microsampling device on *FGB*, the peptides originating from other proteins show a good quantitative correlation with neat plasma, highlighting that the observed behavior is specific for *FGB*. The log_2_(ratio to global standard) from the microsampling device is presented on the *y*-axis, and the log_2_(ratio to global standard) from the neat plasma is presented on the *x*-axis.

### Long-Term Stability Evaluation

To assess the long-term stability of the quantified proteins in the microsampling device while stored at RT, five replicate microsample cards were prepared from venous blood and moved to −20 °C at different times, spanning 232 days. Once all samples had been moved to storage at −20 °C, the samples were prepared for LC–MS/MS analysis, and all peptide quantities were normalized to the predetermined plasma level of albumin in the particular sample (940 pmol/μL). As it had been determined that the *FGB* levels could not be reliably quantified using the microsampling device, *FGB* was excluded from the analysis. For the remaining proteins, a reproducible quantification was obtained across the entire experiment, as visualized at a peptide level in Figure 5A. Figure 5A also highlights the dynamic range that is quantified in this experiment, stretching from roughly 1300 pmol/μL to 10 fmol/μL. It is worthwhile to note the discrepancy in the amount provided by the two peptides from *HPX*. The peptide YYCFQGNQFLR resides in a repeat sequence and, therefore, provides a higher molar amount, while SWPAVGNCSSALR spans two repeats and thus only appears once in the protein sequence. The quantitative stability at the protein level is illustrated in Figure 5B,C, showcasing high stability in most proteins. It is, however, noteworthy that *APOC1* and *APOB* appear to degrade over time, and the most impactful changes happen in the first 50 days. In Figures S3–S22, figures for all proteins at the peptide level can be observed.

**Figure 5:**
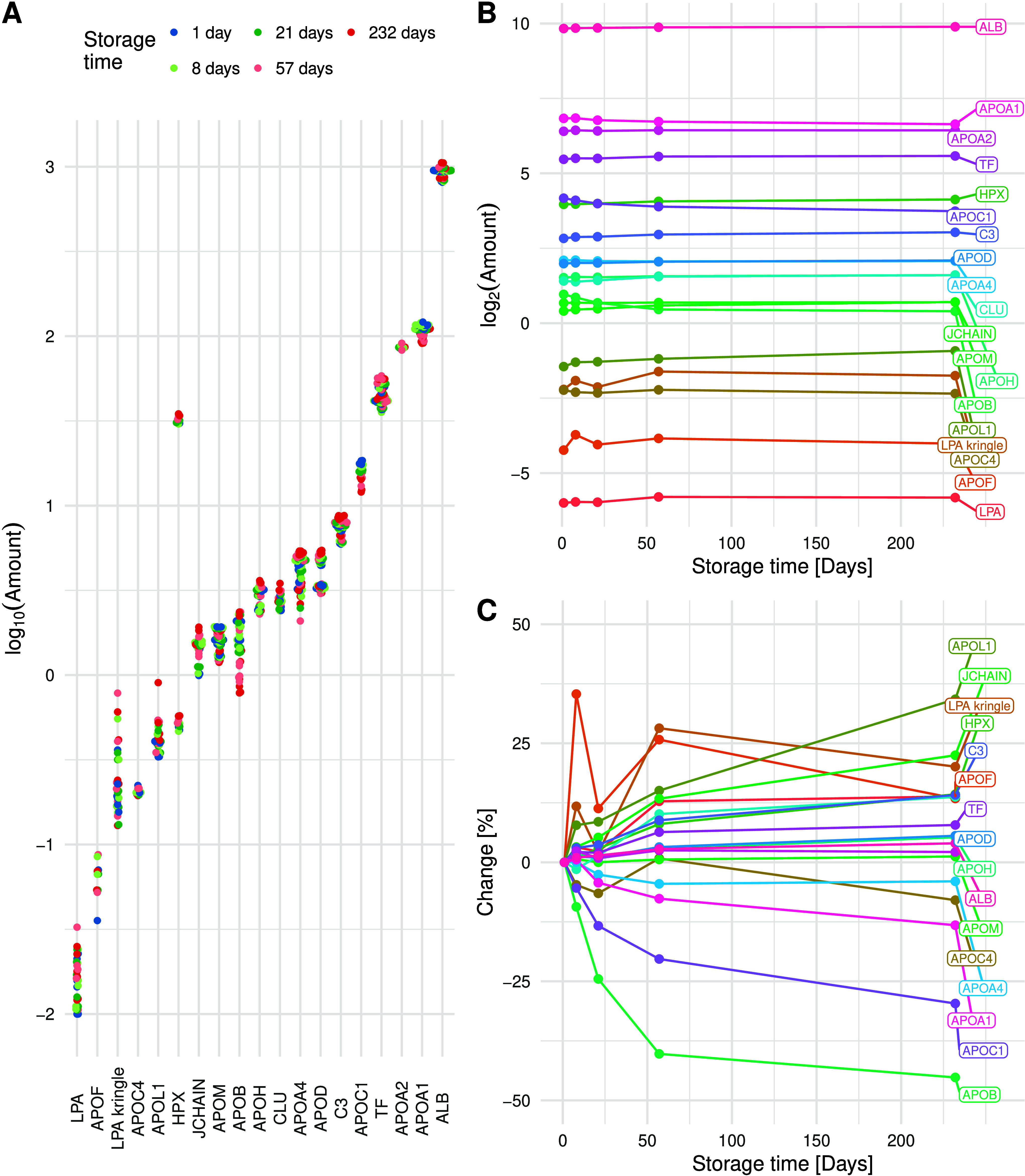
(A) The results obtained for all peptides across the long-term stability experiment were grouped by protein and arranged in the order of abundance. (B) The stability is illustrated at the protein level for the duration of the experiment. (C) The change from the initial quantities measured for each protein is represented as the percentage change compared to day 1. Due to the inconsistent quantification of *FGB*, it has been excluded from all three figures.

## Discussion

Many diseases or health states are multifactorial, and proteomics offers a tool to understand patterns of biological changes while accounting for the necessary biological complexity. By monitoring panels of proteins longitudinally, researchers can better elucidate disease states and progression mechanisms. This contextualizes the expression with regard to other proteins and allows for focusing on the change of a protein’s expression relative to an individual rather than the population.^41^ However, this requires a thorough evaluation of the sample format to ensure good reproducibility and that the targets in question remain informative in the new sample format.^6–9,19,20,30,32^

Here, we assessed a novel device that prepares a cell-free, volumetrically controlled dried plasma-like matrix directly from capillary blood collected through a finger prick. While the quantitative results from the microsampling device and neat plasma were strongly correlated (*R* ^2^ = 0.998), the data from the microsampling device had a larger variance compared to neat plasma (Figure S1). This can, however, easily be rectified by normalizing the data as the observed difference between replicates is consistent across all quantified proteins. There could be various reasons for the observed variance in input material such as differing protein retention during the filtration step within the device or variations in sample volume. Given the consistent differences observed between replicate samples across all quantified proteins except *FGB*, it appears more likely that the observed differences stem from volumetric imprecision between the devices. In order to address the inconsistency between replicates, we have decided to normalize all quantitative data to *ALB,* as it makes up roughly 50% of the total protein amount in the plasma proteome.^42^ Therefore, *ALB* acts as a good proxy for the total protein amount in the sample. Reassuringly, it can also be observed that the correlation between the microsampling device and the venous plasma is maintained no matter what elution buffer is used. However, the AmBic and SDC-based buffers are favored here on the basis of better recovery. It is, however, essential to acknowledge that, as a normalization method is used, the normalized values no longer represent an absolute quantification of the proteins in the original plasma samples, despite the initial quantification of the samples being on an absolute scale. This has to be considered when implementing the devices in a study. If the sampling is used for longitudinal collection of samples, where the trajectories of the quantified proteins are monitored, any potential drifts caused by concentration changes in *ALB* can be observed as increases/decreases in all other proteins and thereby still be observed, whereas when intended for use in a study with a single sample collection point, it might be preferable to avoid the normalization altogether and accept the decrease in precision to accommodate individuals with abnormal albumin levels, such as in the case of hypoalbuminemia.^43^ It is also important to note that any abnormal protein levels should already be observable in the non-normalized quantification data, which should be thoroughly assessed before normalization.

From the quantitative benchmarking experiment conducted in nine healthy donors, it is evident that the quantitative data obtained after normalization achieve a very good correlation (*R* ^2^ > 0.99) between the quantities obtained in plasma and a quantitative precision (median CV = 1.6%) that is slightly higher than the precision obtained in neat plasma (Figure 3B). This can most likely be attributed to the normalization, which accounts for the somewhat varying input material in the case of the microsampling samples. In contrast, this normalization is not performed on the neat plasma samples, where the precision of the pipettes will impact the pipetting of the initial sample. We observe minimal differences between the normalized quantities obtained from the microsampling devices and the absolute quantities obtained in neat plasma. This indicates that the sampling is robust, and the results are translatable between the microsampling device and neat plasma.

The results for *FGB* are outliers that differ drastically from the quantities obtained in neat plasma, and the peptide levels are consistently lower in the microsampling device (Figure 2B, Figure 3A, D). As the DPS separation occurs rapidly in the device through microfluidic filtration, while centrifugation for preparation of neat plasma takes at least 10 min, only accounting for the centrifugation step, this could be a reason for the observed discrepancy. To further elucidate this behavior, we wanted to see how the peptides from *FGB* behaved when plasma was loaded into the microsampling device. This experiment, however, revealed that the observed *FGB* peptides remain consistently lower, even when loading plasma in the microsampling device (Figure 4A,B). This indicates that the dysregulation is not caused by coagulation in the device but rather by some other mechanism. The variance observed in the peptides also increased after being processed through the microsampling device, indicating that the filtration step directly affects the peptides from *FGB*. As *FGB*, together with *FGA* and *FGG*, makes up fibrinogen, a possible explanation for the decrease in protein level during sample processing in the microsampling device is that the device does not allow fibrins to pass through the filter, while fibrinogen, which is a smaller molecule, can be allowed to pass through the filter. As this separation does not occur in traditional plasma preparation, the levels of the fibrinogen peptides will not be affected in conventional plasma.^44^ This, however, needs to be explored further to fully elucidate this artifact.

Microsampling has been proposed not only as a solution for improved sampling frequency and decreased patient burden but also as an improved way of storing samples. This is both from a space perspective and also as the dried format itself improves the stability of the sample.^14,45–47^ From our data, the stability appears to be relatively high across the 232-day-long experiment. It is noteworthy to bear in mind that these samples were stored in a box at RT without any additional desiccants. Still, we observed very stable protein levels across the entire experiment. This indicates that there are benefits to storing the samples in a dried format that can aid in preventing protein degradation compared to plasma.

Interestingly, two protein targets stand out in the data set, and these are *APOB* and *APOC1,* which decrease over time during the stability experiment. This indicates that some degradation or modification of the proteins takes place when stored at room temperature for an extended time, which directly impacts the peptides quantified. However, in the case of *APOB,* the peptides IEGNLIFP­NNYLPK and ESMLK appear more stable over the entire experiment, highlighting that the choice of which peptides are used for quantification can circumvent these problems for experiments where long-term storage at RT is required. In some instances, a decrease in peptide levels occurs in other proteins as well, despite appearing stable at a protein level, which can be seen in Figures S3–S22. As the standards used provide unmodified peptides upon digestion, this indicates a behavior similar to the case discussed above, where modifications or degradations of the amino acids in the quantified peptides would result in a decreased ratio to the standard over time. As this behavior directly impacts the quantification, it further highlights the importance of choosing quantitative peptides that remain stable under the storage conditions intended for the study. As has been seen in experiments from other groups, the effects impacting the stability of the proteins also appear to be the largest at the beginning of the experiment.^47^ This indicates that guidelines for storing the samples after sample collection have to be well-defined prior to the initiation of a study.

## Conclusion

We conducted a thorough quantitative evaluation and benchmarking of a novel microsampling device for readily preparing DPS from finger pricks. Our findings show that the samples prepared using the microsampling device showed high precision, with the majority of the peptides exhibiting a CV below 10% and a median CV of 1.6% in a cohort of nine healthy donors based on a biomarker panel consisting of 19 proteins (57 peptides). There is a noticeable variation in the amount sampled when using the microsampling device. Yet this variance is easily overcome by normalizing the peptide quantities to *ALB,* and the volumetric sampling still serves as a valuable way of ensuring that the spiked-in standards are added in an appropriate amount. Our data also show a high correlation between the quantitative results obtained in plasma and those obtained using the microsampling device, indicating that the quantitative results can be translated between the two sample types. However, certain targets, like *FGB*, are directly impacted by the sample collection method. Therefore, it will always be essential to validate the targets in a target-specific way to ensure that the sampling method does not impact the analytes of interest. The results also show that for quite a few of the targets, the dried format helps stabilize the targets and that the samples can even be stored for seven months at RT without drastically impacting the quantitative results. However, as with all sample collection, it is advised to ensure that a standardized method of handling the samples after collection is well established. Our findings suggest that the microsampling device for preparing DPS straight from capillary blood is very promising and has a high potential to drastically increase the samples that can be collected in a clinical trial without a significant patient burden and thereby provide a more detailed characterization of the effects of drugs evaluated in clinical trials at a patient-centric level.

## Supplementary Material



## Notes

Table S1 listing composition of the pretiterd standard mixture and the spike-in level for each standard per sample; Table S2 showing the transition list for the final method, including the corresponding collision energies; Table S3 listing LC-gradients used for the experiments; Figure S1 showing a comparison of normalized and non-normalized data from samples prepared with the microsampling device; Figure S2 showing two boxplots illustrating the precision of the quantification in both the microsampling device and in neat plasma for nine healthy donors; Figures S3–S22 showing separate figures for all target proteins quantified in the stability experiment showing the change in quantity over time at a peptide level (PDF)
