# Supplementary material for: Quantitative Assessment of a Novel Device Designed for Patient-centric Sampling of Dried Plasma Using Targeted Proteomics
Source: Anal Chem. 2025 Jun 18;97(25):12953–62. doi: 10.1021/acs.analchem.4c05455 (PMC12224170; doi:10.1021/acs.analchem.4c05455)
Supplement: Supplementary file 1 [file ac4c05455_si_001.pdf]

Supporting Information

Quantitative assessment of a novel device designed for patient-centric sampling of dried plasma using targeted proteomics

Andreas Hober[1]*, Marcus Henricsson[1], Tim Ruckh[2], Pia Davidsson[1], Benjamin Challis[3] and Tasso Miliotis[1]*

1. Translational Science & Experimental Medicine, Research and Early Development, Cardiovascular, Renal and Metabolism (CVRM), BioPharmaceuticals R&D, AstraZeneca, 431 83 Gothenburg, Sweden
2. R&D Digital Health, AstraZeneca, 431 83 Gothenburg, Sweden
3. Translational Science & Experimental Medicine, Research and Early Development, Cardiovascular, Renal and Metabolism (CVRM), BioPharmaceuticals R&D, AstraZeneca, Cambridge CB2 0AA, UK

Table of Contents

Table S1 - S2
Table S2 - S3
Table S3 - S20
Figure S1 - S21
Figure S2 - S22
Figure S3 - S23
Figure S4 - S24
Figure S5 - S25
Figure S6 - S26
Figure S7 - S27
Figure S8 - S28
Figure S9 - S29
Figure S10 - S30
Figure S11 - S31
Figure S12 - S32
Figure S13 - S33
Figure S14 - S34
Figure S15 - S35
Figure S16 - S36
Figure S17 - S37
Figure S18 - S38
Figure S19 - S39
Figure S20 - S40
Figure S21 - S41
Figure S22 - S42

Table S1

PrEST	Gene	Uniprot	Sequence	Pool conc. [uM]	Amount per sample [pmol]
HPRR2760276	ALB	P02768	EPERNECFLQHKDDNPNLPRLVRPEVDVMCTAFHDNEETFLKKYLYEIARRHPYFYAPELLFFAKRYKAAFTECCQAADKAACLLPKLDELRDEGKASSAKQRLKCAS	20.9	100
HPRR3450266	APOA1	P02647	LSPLGEEMRDRARAHVDALRTHLAPYSDELRQRLAARLEALKENGGARLAEYHAKATEHLSTLSEKAKPALEDLRQGLLPVLESFKVSFLSALEEYTKKLNTQ	2.03	9.74
HPRR4430020	APOA2	P02652	ALVRRQAKEPCVESLVSQYFQTVTDYGKDLMEKVKSPELQAEAKSY	2.20	10.5
HPRR260124	APOA4	P06727	LEGLTFQMKKNAEELKARISASAEELRQRLAPLAEDVRGNLRGNTEGLQKSLAELGGHLDQQVEEFRRRVEPYGENFNKALVQQMEQLRQKLGPHAGDVEGHLSFLEKDLRDKVNSFFSTFKEKESQDKTLSLP	0.0366	0.176
HPRR5000480	APOA5	Q6Q788	RRQLQEELEEVKARLQPYMAEAHELVGWNLEGLRQQLKPYTMDLMEQVALRVQELQEQLRVVGEDTKAQLLGGVDEAWALLQGLQSRVVHHT	0.0507	0.243
HPRR3720311	APOB	P04114	NFVASHIANILNSEELDIQDLKKLVKEALKESQLPTVMDFRKFSRNYQLYKSVSLPSLDPASAKIEGNLIFDPNNYLPKESMLKTTLTAFGFASADLIE	0.339	1.62
HPRR3730489	APOC1	P02654	PDVSSALDKLKEFGNTLEDKARELISRIKQSELSAKMREWFSETFQKVKE	0.301	1.44
HPRR2190004	APOC3	P02656	SEAEDASLLSFMQGYMKHATKTAKDALSSVQESQVAQQARGWVTDGFSSLKDYWSTVKDKFSEFWDLDPEVRPTSAVAA	0.0539	0.258
HPRR4130067	APOC4	P55056	QTYYDDHLRDLGPLTKAWFLESKDSLLKKTHSLCPRLVCGDKDQG	0.00752	0.036
HPRR2760373	APOD	P05090	GKCPNPPVQENFDVNKYLGRWYEIEKIPTTFENGRCIQANYSLMENGKIKVLNQELRADGTVNQIEGE	0.171	0.822
HPRR350023	APOF	Q13790	YGKQTNVLMHFPLSLESQTPSSDPLSCQFLHPKSLPGFSHMAPLPKFLVSLALRNALEEAGCQADVWALQLQLYRQGGVNATQVLIQHLRGLQKGRSTERNVSVEALASALQLLAREQQSTGRVGRSLPTEDCENEKEQA	0.00585	0.028
HPRR5000011	APOH	P02749	RTCPKPDDLPFSTVVPLKTFYEPGEEITYSCKPGYVSRGGMRKFICPLTGLWPINTLKCTPRVCPFAGILENGAVRYTTFEYPNTISFSCNTGFYLNGADSAKCTEEGKWSPELPVCAPIICPPPSIPTFATLRVYKPS	0.510	2.44
HPRR350088	APOL1	O14791	SNFLSLAGNTYQLTRGIGKDIRALRRARANLQSVPHASASRPRVTEPISAESGEQVERVNEPSILEMSRGVKLTDVAPVSFFLVLDVVYLVYESKHLHEGAKSETAEELKKVAQELEEKLNILNN	0.0884	0.423
HPRR3720433	APOL4	Q9BPW4	TSTDQLEALRDILRDITPNVLSFALDFDEATKMIANDVHTLRRSKATVGRPLIAWRYVPINVVETLRTRGAPTRIVRKVARNL	0.0207	0.099
HPRR3340379	APOM	O95445	KDGLCVPRKWIYHLTEGSTDLRTEGRPDMKTELFSSSCPGGIMLNETGQGYQRFLLYNRSPHPPEKCVEEFKSLTSCLDSKAFLLTPRNQ	0.208	0.995
HPRR280090	C3	P01024	KVRVELLHNPAFCSLATTKRRHQQTVTIPPKSSLSVPYVIVPLKTGLQEVEVKAAVYHHFISDGVRKSLKVVPEGIRMNKTVAVRTLDPERLGREGVQKEDIPPADLSDQVPDTESETRILLQGTPVAQMTEDAVDAERLKHL	1.90	9.08
HPRR3720003	CFH	P08603	CNELPPRRNTEILTGSWSDQTYPEGTQAIYKCRPGYRSLGNVIMVCRKGEWVALNPLRKCQKRPCGHPGDTPFGTFTLTGGNVFEYGVKA	0.215	1.03
HPRR4320626	CLU	P10909	KEDALNETRESETKLKELPGVCNETMMALWEECKPCLKQTCMKFYARVCRSGSGLVGRQLEEFLNQSSPFYFWMNGDRIDSLLENDRQQTHMLDVMQDH	0.0557	0.267
HPRR3761208	FGA	P02671	GHWTSESSVSGSTGQWHSESGSFRPDSPGSGNARPNNPDWGTFEEVSGNVSPGTRREYHTEKLVTSKGDKELRTGKEKVTSGSTTTTRRSCSKTVTKT	0.380	1.82
HPRR350021	FGB	P02675	VIQNRQDGSVDFGRKWDPYKQGFGNVATNTDGKNYCGLPGEYWLGNDKISQLTRMGPTELLIEMEDWKGDKVKAHYGGFTVQNEANKYQISVNKYRGTAGNALMDGASQLMGENRTMTIHNGMFFSTYDRDNDGWLTSDPRKQC	0.0681	0.326
HPRR4220446	FGG	P02679	GWWMNKCHAGHLNGVYYQGGTYSKASTPNGYDNGIIWATWKTRWYSMKKTTMKIIPFNRLTIGEGQQHHLGGAKQVRPEHPAETEYDSLYP	0.0212	0.102
HPRR4200150	HPX	P02790	ECHRGECQAEGVLFFQGDREWFWDLATGTMKERSWPAVGNCSSALRWLGRYYCFQGNQFLRFDPVRGEVPPRYPRDVRDYFMPCP	0.421	2.02
HPRR350060	JCHAIN	P01591	VDNKCKCARITSRIIRSSEDPNEDIVERNIRIIVPLNNRENISDPTSPLRTRFVYHLSDLCKKCDPTEVELDNQIVTATQSNICDEDSATETCYTYDRNKCYTAVVPLVYGGETKMVETA	0.573	2.74
HPRR5000605	LPA	P08519	SSYKVILGAHQEVNLESHVQEIEVSRLFLEPTQADIALLKLSRPAVITDKVMPACLPSPDYMVTARTECYITGWGETQGTFGTGLLKEAQLLVIENEVCNHYKYICAEHLARGTDSCQGDSGGPLVCFEKDKYILQG	0.0119	0.057
HPRR5000606	LPA kringle	P08519	APTEQRPGVQECYHGNGQSYRGTYSTTVTGRTCQAWSSMTPHSHSRTPEYYPNAGLIMNYCRNPDPVAAPYCYTRDPSVRWEYCNLTQCSDAEGTAVAPPTITPIPSLEAPSEQ	0.208	0.997
HPRR5000484	TF	P02787	KDGAGDVAFVKHSTIFENLANKADRDQYELLCLDNTRKPVDEYKDCHLAQVPSHTVVARSMGGKEDLIWELLNQAQEHFGKDKSKEFQLFSSPHGKDLLFKDSAHGFLKVPPRM	0.246	1.18

Table S2

Compound	Retention Time (min)	RT Window (min)	Precursor (m/z)	Product (m/z)	Collision Energy (V)	Min Dwell Time (ms)
C[+57.021464]AQESSVVR(+2)	6.54	2	518.248	360.134	17	8.587
C[+57.021464]AQESSVVR(+2)	6.54	2	518.248	547.32	17	8.587
C[+57.021464]AQESSVVR(+2)	6.54	2	518.248	676.362	17	8.587
C[+57.021464]AQESSVVR(+2)	6.54	2	518.248	804.421	17	8.587
C[+57.021464]AQESSVVR (heavy)(+2)	6.54	2	523.252	360.134	17	8.587
C[+57.021464]AQESSVVR (heavy)(+2)	6.54	2	523.252	557.328	17	8.587
C[+57.021464]AQESSVVR (heavy)(+2)	6.54	2	523.252	686.371	17	8.587
C[+57.021464]AQESSVVR (heavy)(+2)	6.54	2	523.252	814.429	17	8.587
SC[+57.021464]EVPTR(+2)	6.59	2	424.7	373.219	13.9	8.583
SC[+57.021464]EVPTR(+2)	6.59	2	424.7	377.113	13.9	8.583
SC[+57.021464]EVPTR(+2)	6.59	2	424.7	472.288	13.9	8.583
SC[+57.021464]EVPTR(+2)	6.59	2	424.7	601.33	13.9	8.583
SC[+57.021464]EVPTR (heavy)(+2)	6.59	2	429.704	377.113	13.9	8.583
SC[+57.021464]EVPTR (heavy)(+2)	6.59	2	429.704	383.228	13.9	8.583
SC[+57.021464]EVPTR (heavy)(+2)	6.59	2	429.704	482.296	13.9	8.583
SC[+57.021464]EVPTR (heavy)(+2)	6.59	2	429.704	611.339	13.9	8.583
TTC[+57.021464]WDGK(+2)	7.22	2	434.186	333.139	14.2	5.45
TTC[+57.021464]WDGK(+2)	7.22	2	434.186	505.241	14.2	5.45
TTC[+57.021464]WDGK(+2)	7.22	2	434.186	664.24	14.2	5.45
TTC[+57.021464]WDGK(+2)	7.22	2	434.186	665.271	14.2	5.45
TTC[+57.021464]WDGK (heavy)(+2)	7.22	2	438.194	337.146	14.2	5.45
TTC[+57.021464]WDGK (heavy)(+2)	7.22	2	438.194	513.255	14.2	5.45
TTC[+57.021464]WDGK (heavy)(+2)	7.22	2	438.194	664.24	14.2	5.45
TTC[+57.021464]WDGK (heavy)(+2)	7.22	2	438.194	673.285	14.2	5.45
YGVSDYHK (heavy)(+2)	7.34	2	488.734	657.308	15.9	5.448
YGVSDYHK (heavy)(+2)	7.34	2	488.734	756.377	15.9	5.448
YGVSDYHK (heavy)(+2)	7.34	2	488.734	813.398	15.9	5.448
AASGTQNNVLR(+2)	7.51	2	565.799	615.357	18.6	4.978
AASGTQNNVLR(+2)	7.51	2	565.799	743.416	18.6	4.978
AASGTQNNVLR(+2)	7.51	2	565.799	901.485	18.6	4.978
AASGTQNNVLR(+2)	7.51	2	565.799	988.517	18.6	4.978
AASGTQNNVLR (heavy)(+2)	7.51	2	570.803	625.366	18.6	4.978
AASGTQNNVLR (heavy)(+2)	7.51	2	570.803	753.424	18.6	4.978
AASGTQNNVLR (heavy)(+2)	7.51	2	570.803	911.493	18.6	4.978
AASGTQNNVLR (heavy)(+2)	7.51	2	570.803	998.525	18.6	4.978
GSHMASLAEAK (heavy)(+3)	7.55	2	370.521	213.626	10.7	4.978
GSHMASLAEAK (heavy)(+3)	7.55	2	370.521	426.244	10.7	4.978
GSHMASLAEAK (heavy)(+3)	7.55	2	370.521	571.229	10.7	4.978
TLYSSSPR(+2)	7.63	2	455.735	348.669	13.8	4.978
TLYSSSPR(+2)	7.63	2	455.735	378.202	14.9	4.978
TLYSSSPR(+2)	7.63	2	455.735	533.268	15.8	4.978
TLYSSSPR(+2)	7.63	2	455.735	696.331	13.8	4.978
TLYSSSPR (heavy)(+2)	7.63	2	460.739	353.673	13.8	4.978
TLYSSSPR (heavy)(+2)	7.63	2	460.739	378.202	14.9	4.978
TLYSSSPR (heavy)(+2)	7.63	2	460.739	543.276	15.8	4.978
TLYSSSPR (heavy)(+2)	7.63	2	460.739	706.339	13.8	4.978
NLINNAK (heavy)(+2)	7.65	2	397.734	340.207	12.9	4.978
NLINNAK (heavy)(+2)	7.65	2	397.734	341.218	12.9	4.978
NLINNAK (heavy)(+2)	7.65	2	397.734	454.25	12.9	4.978
VYVPQSR(+2)	7.94	2	424.734	487.262	13.9	4.191
VYVPQSR(+2)	7.94	2	424.734	586.331	13.9	4.191
VYVPQSR(+2)	7.94	2	424.734	749.394	13.9	4.191
VYVPQSR (heavy)(+2)	7.94	2	429.739	497.271	13.9	4.191
VYVPQSR (heavy)(+2)	7.94	2	429.739	596.339	13.9	4.191
VYVPQSR (heavy)(+2)	7.94	2	429.739	759.402	13.9	4.191
YRPSQDQGLPASR(+3)	8.07	2	492.251	215.624	14.2	4.135
YRPSQDQGLPASR(+3)	8.07	2	492.251	430.241	14.2	4.135
YRPSQDQGLPASR(+3)	8.07	2	492.251	600.346	14.2	4.135
YRPSQDQGLPASR (heavy)(+3)	8.07	2	498.923	220.628	14.2	4.135
YRPSQDQGLPASR (heavy)(+3)	8.07	2	498.923	440.249	14.2	4.135
YRPSQDQGLPASR (heavy)(+3)	8.07	2	498.923	610.355	14.2	4.135
LIQEAPKPEC[+57.021464]EK(+3)	8.1	2	481.25	479.737	13.9	4.135
LIQEAPKPEC[+57.021464]EK(+3)	8.1	2	481.25	544.258	13.9	4.135
LIQEAPKPEC[+57.021464]EK(+3)	8.1	2	481.25	608.287	13.9	4.135
LIQEAPKPEC[+57.021464]EK(+3)	8.1	2	481.25	664.829	13.9	4.135
LIQEAPKPEC[+57.021464]EK (heavy)(+3)	8.1	2	486.592	487.751	13.9	4.135
LIQEAPKPEC[+57.021464]EK (heavy)(+3)	8.1	2	486.592	552.272	13.9	4.135
LIQEAPKPEC[+57.021464]EK (heavy)(+3)	8.1	2	486.592	616.302	13.9	4.135
LIQEAPKPEC[+57.021464]EK (heavy)(+3)	8.1	2	486.592	672.844	13.9	4.135
SLEC[+57.021464]LHPGTK(+3)	8.32	2	381.193	402.235	14.8	3.815
SLEC[+57.021464]LHPGTK(+3)	8.32	2	381.193	406.708	12.8	3.815
SLEC[+57.021464]LHPGTK(+3)	8.32	2	381.193	471.229	10.8	3.815
SLEC[+57.021464]LHPGTK(+3)	8.32	2	381.193	652.378	14.8	3.815

SLEC[+57.021464]LHPGTK (heavy)(+3)	8.32	2	383.865	410.249	14.8	3.815
SLEC[+57.021464]LHPGTK (heavy)(+3)	8.32	2	383.865	410.715	12.8	3.815
SLEC[+57.021464]LHPGTK (heavy)(+3)	8.32	2	383.865	475.236	10.8	3.815
SLEC[+57.021464]LHPGTK (heavy)(+3)	8.32	2	383.865	660.392	14.8	3.815
SDIAPVAR(+2)	8.33	2	414.732	316.15	13.6	3.815
SDIAPVAR(+2)	8.33	2	414.732	442.277	13.6	3.815
SDIAPVAR(+2)	8.33	2	414.732	513.314	13.6	3.815
SDIAPVAR (heavy)(+2)	8.33	2	419.736	316.15	13.6	3.815
SDIAPVAR (heavy)(+2)	8.33	2	419.736	452.286	13.6	3.815
SDIAPVAR (heavy)(+2)	8.33	2	419.736	523.323	13.6	3.815
EGTMLNC[+57.021464]EC[+57.021464]K(+2)	8.33	2	621.251	596.217	20.4	3.815
EGTMLNC[+57.021464]EC[+57.021464]K(+2)	8.33	2	621.251	710.26	20.4	3.815
EGTMLNC[+57.021464]EC[+57.021464]K(+2)	8.33	2	621.251	823.344	20.4	3.815
EGTMLNC[+57.021464]EC[+57.021464]K(+2)	8.33	2	621.251	954.384	20.4	3.815
EGTMLNC[+57.021464]EC[+57.021464]K (heavy)(+2)	8.33	2	625.258	604.231	20.4	3.815
EGTMLNC[+57.021464]EC[+57.021464]K (heavy)(+2)	8.33	2	625.258	718.274	20.4	3.815
EGTMLNC[+57.021464]EC[+57.021464]K (heavy)(+2)	8.33	2	625.258	831.358	20.4	3.815
EGTMLNC[+57.021464]EC[+57.021464]K (heavy)(+2)	8.33	2	625.258	962.398	20.4	3.815
LYYGDDEK(+2)	8.35	2	501.724	391.182	16.4	3.815
LYYGDDEK(+2)	8.35	2	501.724	445.182	16.4	3.815
LYYGDDEK(+2)	8.35	2	501.724	563.231	16.4	3.815
LYYGDDEK(+2)	8.35	2	501.724	726.294	16.4	3.815
LYYGDDEK(+2)	8.35	2	501.724	889.357	16.4	3.815
LYYGDDEK (heavy)(+2)	8.35	2	505.731	399.197	16.4	3.815
LYYGDDEK (heavy)(+2)	8.35	2	505.731	449.189	16.4	3.815
LYYGDDEK (heavy)(+2)	8.35	2	505.731	571.245	16.4	3.815
LYYGDDEK (heavy)(+2)	8.35	2	505.731	734.308	16.4	3.815
LYYGDDEK (heavy)(+2)	8.35	2	505.731	897.372	16.4	3.815
GSEAINAPGDNPAK(+2)	8.56	2	670.825	345.14	21.1	3.815
GSEAINAPGDNPAK(+2)	8.56	2	670.825	698.347	19.1	3.815
GSEAINAPGDNPAK(+2)	8.56	2	670.825	769.384	21.1	3.815
GSEAINAPGDNPAK(+2)	8.56	2	670.825	883.427	19.1	3.815
GSEAINAPGDNPAK (heavy)(+2)	8.56	2	674.832	345.14	21.1	3.815
GSEAINAPGDNPAK (heavy)(+2)	8.56	2	674.832	706.361	19.1	3.815
GSEAINAPGDNPAK (heavy)(+2)	8.56	2	674.832	777.398	21.1	3.815
GSEAINAPGDNPAK (heavy)(+2)	8.56	2	674.832	891.441	19.1	3.815
ELDKYGVSDYHK (heavy)(+3)	8.79	2	490.579	329.17	14	3.535
ELDKYGVSDYHK (heavy)(+3)	8.79	2	490.579	614.302	14	3.535
ELDKYGVSDYHK (heavy)(+3)	8.79	2	490.579	657.333	14	3.535
AAIISAEGDSK(+2)	8.86	2	531.277	256.166	17.4	3.535
AAIISAEGDSK(+2)	8.86	2	531.277	460.24	17.4	3.535
AAIISAEGDSK(+2)	8.86	2	531.277	693.305	17.4	3.535
AAIISAEGDSK(+2)	8.86	2	531.277	806.389	17.4	3.535
AAIISAEGDSK (heavy)(+2)	8.86	2	535.284	256.166	17.4	3.535
AAIISAEGDSK (heavy)(+2)	8.86	2	535.284	464.247	17.4	3.535
AAIISAEGDSK (heavy)(+2)	8.86	2	535.284	701.319	17.4	3.535
AAIISAEGDSK (heavy)(+2)	8.86	2	535.284	814.403	17.4	3.535
VFQQVAQASK(+2)	8.86	2	553.303	430.235	18.2	3.535
VFQQVAQASK(+2)	8.86	2	553.303	603.346	18.2	3.535
VFQQVAQASK(+2)	8.86	2	553.303	731.405	18.2	3.535
VFQQVAQASK(+2)	8.86	2	553.303	859.463	18.2	3.535
VFQQVAQASK (heavy)(+2)	8.86	2	557.31	434.242	18.2	3.535
VFQQVAQASK (heavy)(+2)	8.86	2	557.31	611.36	18.2	3.535
VFQQVAQASK (heavy)(+2)	8.86	2	557.31	739.419	18.2	3.535
VFQQVAQASK (heavy)(+2)	8.86	2	557.31	867.477	18.2	3.535
VVPEGIR(+2)	8.99	2	385.231	286.164	12.6	3.535
VVPEGIR(+2)	8.99	2	385.231	345.224	12.6	3.535
VVPEGIR(+2)	8.99	2	385.231	474.267	12.6	3.535
VVPEGIR(+2)	8.99	2	385.231	571.32	12.6	3.535
VVPEGIR (heavy)(+2)	8.99	2	390.236	291.168	12.6	3.535
VVPEGIR (heavy)(+2)	8.99	2	390.236	355.233	12.6	3.535
VVPEGIR (heavy)(+2)	8.99	2	390.236	484.275	12.6	3.535
VVPEGIR (heavy)(+2)	8.99	2	390.236	581.328	12.6	3.535
VVEESELAR(+2)	9.01	2	516.272	328.187	16.9	3.535
VVEESELAR(+2)	9.01	2	516.272	575.315	16.9	3.535
VVEESELAR(+2)	9.01	2	516.272	704.357	16.9	3.535
VVEESELAR(+2)	9.01	2	516.272	833.4	16.9	3.535
VVEESELAR(+2)	9.01	2	516.272	932.468	16.9	3.535
VVEESELAR (heavy)(+2)	9.01	2	521.276	328.187	16.9	3.535
VVEESELAR (heavy)(+2)	9.01	2	521.276	585.323	16.9	3.535
VVEESELAR (heavy)(+2)	9.01	2	521.276	714.366	16.9	3.535
VVEESELAR (heavy)(+2)	9.01	2	521.276	843.408	16.9	3.535
VVEESELAR (heavy)(+2)	9.01	2	521.276	942.477	16.9	3.535
AGLQVYNK(+2)	9.11	2	446.747	242.15	14.6	3.535
AGLQVYNK(+2)	9.11	2	446.747	382.719	14.6	3.535
AGLQVYNK(+2)	9.11	2	446.747	523.287	14.6	3.535
AGLQVYNK(+2)	9.11	2	446.747	651.346	14.6	3.535

AGLQVYNK (heavy)(+2)	9.11	2	450.755	242.15	14.6	3.535
AGLQVYNK (heavy)(+2)	9.11	2	450.755	386.726	14.6	3.535
AGLQVYNK (heavy)(+2)	9.11	2	450.755	531.302	14.6	3.535
AGLQVYNK (heavy)(+2)	9.11	2	450.755	659.36	14.6	3.535
QLNEINYEDHK(+3)	9.21	2	468.224	399.199	13.5	3.535
QLNEINYEDHK(+3)	9.21	2	468.224	403.177	13.5	3.535
QLNEINYEDHK(+3)	9.21	2	468.224	528.241	13.5	3.535
QLNEINYEDHK(+3)	9.21	2	468.224	581.262	13.5	3.535
QLNEINYEDHK(+3)	9.21	2	468.224	691.305	13.5	3.535
QLNEINYEDHK (heavy)(+3)	9.21	2	470.896	407.184	13.5	3.535
QLNEINYEDHK (heavy)(+3)	9.21	2	470.896	407.213	13.5	3.535
QLNEINYEDHK (heavy)(+3)	9.21	2	470.896	536.255	13.5	3.535
QLNEINYEDHK (heavy)(+3)	9.21	2	470.896	585.269	13.5	3.535
QLNEINYEDHK (heavy)(+3)	9.21	2	470.896	699.319	13.5	3.535
LVGGDNLC[+57.021464]SGR(+2)	9.31	2	574.279	468.204	18.8	3.503
LVGGDNLC[+57.021464]SGR(+2)	9.31	2	574.279	479.203	21.8	3.503
LVGGDNLC[+57.021464]SGR(+2)	9.31	2	574.279	517.738	17.8	3.503
LVGGDNLC[+57.021464]SGR(+2)	9.31	2	574.279	935.4	19.8	3.503
LVGGDNLC[+57.021464]SGR (heavy)(+2)	9.31	2	579.283	473.208	18.8	3.503
LVGGDNLC[+57.021464]SGR (heavy)(+2)	9.31	2	579.283	489.211	21.8	3.503
LVGGDNLC[+57.021464]SGR (heavy)(+2)	9.31	2	579.283	522.742	17.8	3.503
LVGGDNLC[+57.021464]SGR (heavy)(+2)	9.31	2	579.283	945.408	19.8	3.503
TGDAVEFQC[+57.021464]K(+2)	9.46	2	577.76	274.103	23	3.259
TGDAVEFQC[+57.021464]K(+2)	9.46	2	577.76	345.14	20	3.259
TGDAVEFQC[+57.021464]K(+2)	9.46	2	577.76	582.27	20	3.259
TGDAVEFQC[+57.021464]K(+2)	9.46	2	577.76	810.381	19	3.259
TGDAVEFQC[+57.021464]K (heavy)(+2)	9.46	2	581.768	274.103	23	3.259
TGDAVEFQC[+57.021464]K (heavy)(+2)	9.46	2	581.768	345.14	20	3.259
TGDAVEFQC[+57.021464]K (heavy)(+2)	9.46	2	581.768	590.285	20	3.259
TGDAVEFQC[+57.021464]K (heavy)(+2)	9.46	2	581.768	818.396	19	3.259
GMAPALR(+2)	9.48	2	358.199	264.169	11.7	3.259
GMAPALR(+2)	9.48	2	358.199	456.293	11.7	3.259
GMAPALR(+2)	9.48	2	358.199	527.33	11.7	3.259
GMAPALR (heavy)(+2)	9.48	2	363.203	269.173	11.7	3.259
GMAPALR (heavy)(+2)	9.48	2	363.203	466.301	11.7	3.259
GMAPALR (heavy)(+2)	9.48	2	363.203	537.338	11.7	3.259
EFTEAVEAK(+2)	9.65	2	512.253	374.198	16.8	3.062
EFTEAVEAK(+2)	9.65	2	512.253	378.166	16.8	3.062
EFTEAVEAK(+2)	9.65	2	512.253	517.298	16.8	3.062
EFTEAVEAK(+2)	9.65	2	512.253	646.341	16.8	3.062
EFTEAVEAK(+2)	9.65	2	512.253	747.388	16.8	3.062
EFTEAVEAK (heavy)(+2)	9.65	2	516.26	378.166	16.8	3.062
EFTEAVEAK (heavy)(+2)	9.65	2	516.26	378.205	16.8	3.062
EFTEAVEAK (heavy)(+2)	9.65	2	516.26	525.312	16.8	3.062
EFTEAVEAK (heavy)(+2)	9.65	2	516.26	654.355	16.8	3.062
EFTEAVEAK (heavy)(+2)	9.65	2	516.26	755.403	16.8	3.062
ASSFLGEK(+2)	9.77	2	419.718	333.177	13.7	3.06
ASSFLGEK(+2)	9.77	2	419.718	593.329	13.7	3.06
ASSFLGEK(+2)	9.77	2	419.718	680.361	13.7	3.06
ASSFLGEK(+2)	9.77	2	419.718	767.393	13.7	3.06
ASSFLGEK (heavy)(+2)	9.77	2	423.725	341.191	13.7	3.06
ASSFLGEK (heavy)(+2)	9.77	2	423.725	601.344	13.7	3.06
ASSFLGEK (heavy)(+2)	9.77	2	423.725	688.376	13.7	3.06
ASSFLGEK (heavy)(+2)	9.77	2	423.725	775.408	13.7	3.06
FQASVATPR(+2)	9.77	2	488.764	444.257	15	3.06
FQASVATPR(+2)	9.77	2	488.764	543.325	17	3.06
FQASVATPR(+2)	9.77	2	488.764	630.357	17	3.06
FQASVATPR(+2)	9.77	2	488.764	701.394	16	3.06
FQASVATPR (heavy)(+2)	9.77	2	493.768	454.265	15	3.06
FQASVATPR (heavy)(+2)	9.77	2	493.768	553.333	17	3.06
FQASVATPR (heavy)(+2)	9.77	2	493.768	640.365	17	3.06
FQASVATPR (heavy)(+2)	9.77	2	493.768	711.402	16	3.06
SIELAEAK (heavy)(+2)	9.78	2	434.746	426.244	14.1	3.06
SIELAEAK (heavy)(+2)	9.78	2	434.746	539.328	13	3.06
SIELAEAK (heavy)(+2)	9.78	2	434.746	668.37	14.1	3.06
QLVC[+57.021464]PVTR(+2)	9.78	2	486.768	316.663	14.9	3.06
QLVC[+57.021464]PVTR(+2)	9.78	2	486.768	341.218	15.9	3.06
QLVC[+57.021464]PVTR(+2)	9.78	2	486.768	632.318	16.9	3.06
QLVC[+57.021464]PVTR(+2)	9.78	2	486.768	731.387	18.9	3.06
QLVC[+57.021464]PVTR (heavy)(+2)	9.78	2	491.772	321.667	14.9	3.06
QLVC[+57.021464]PVTR (heavy)(+2)	9.78	2	491.772	341.218	15.9	3.06
QLVC[+57.021464]PVTR (heavy)(+2)	9.78	2	491.772	642.327	16.9	3.06
QLVC[+57.021464]PVTR (heavy)(+2)	9.78	2	491.772	741.395	18.9	3.06
NNANGVDLNR(+2)	9.83	2	543.767	300.13	17.8	3.06
NNANGVDLNR(+2)	9.83	2	543.767	517.273	17.8	3.06
NNANGVDLNR(+2)	9.83	2	543.767	787.406	17.8	3.06
NNANGVDLNR(+2)	9.83	2	543.767	858.443	17.8	3.06

NNANGVDLNR (heavy)(+2)	9.83	2	548.772	300.13	17.8	3.06
NNANGVDLNR (heavy)(+2)	9.83	2	548.772	527.281	17.8	3.06
NNANGVDLNR (heavy)(+2)	9.83	2	548.772	797.414	17.8	3.06
NNANGVDLNR (heavy)(+2)	9.83	2	548.772	868.451	17.8	3.06
GSESGIFTNTK(+2)	10.04	2	570.78	463.251	18.7	3.06
GSESGIFTNTK(+2)	10.04	2	570.78	610.32	18.7	3.06
GSESGIFTNTK(+2)	10.04	2	570.78	780.425	18.7	3.06
GSESGIFTNTK(+2)	10.04	2	570.78	867.457	18.7	3.06
GSESGIFTNTK (heavy)(+2)	10.04	2	574.787	471.265	18.7	3.06
GSESGIFTNTK (heavy)(+2)	10.04	2	574.787	618.334	18.7	3.06
GSESGIFTNTK (heavy)(+2)	10.04	2	574.787	788.439	18.7	3.06
GSESGIFTNTK (heavy)(+2)	10.04	2	574.787	875.471	18.7	3.06
EDVVTNGIGR(+2)	10.2	2	530.275	344.145	17.4	3.06
EDVVTNGIGR(+2)	10.2	2	530.275	402.246	17.4	3.06
EDVVTNGIGR(+2)	10.2	2	530.275	617.337	17.4	3.06
EDVVTNGIGR(+2)	10.2	2	530.275	716.405	17.4	3.06
EDVVTNGIGR (heavy)(+2)	10.2	2	535.279	344.145	17.4	3.06
EDVVTNGIGR (heavy)(+2)	10.2	2	535.279	412.254	17.4	3.06
EDVVTNGIGR (heavy)(+2)	10.2	2	535.279	627.345	17.4	3.06
EDVVTNGIGR (heavy)(+2)	10.2	2	535.279	726.413	17.4	3.06
QSLQELPAGSR(+2)	10.27	2	593.314	487.262	19.5	3.06
QSLQELPAGSR(+2)	10.27	2	593.314	600.346	19.5	3.06
QSLQELPAGSR(+2)	10.27	2	593.314	729.389	19.5	3.06
QSLQELPAGSR(+2)	10.27	2	593.314	857.448	19.5	3.06
QSLQELPAGSR(+2)	10.27	2	593.314	867.457	19.5	3.06
QSLQELPAGSR (heavy)(+2)	10.27	2	598.318	497.271	19.5	3.06
QSLQELPAGSR (heavy)(+2)	10.27	2	598.318	610.355	19.5	3.06
QSLQELPAGSR (heavy)(+2)	10.27	2	598.318	739.397	19.5	3.06
QSLQELPAGSR (heavy)(+2)	10.27	2	598.318	867.456	19.5	3.06
QSLQELPAGSR (heavy)(+2)	10.27	2	598.318	867.457	19.5	3.06
FEHC[+57.021464]NFNDVTTR(+3)	10.3	2	513.893	377.214	14.8	3.06
FEHC[+57.021464]NFNDVTTR(+3)	10.3	2	513.893	476.283	14.8	3.06
FEHC[+57.021464]NFNDVTTR(+3)	10.3	2	513.893	532.698	14.8	3.06
FEHC[+57.021464]NFNDVTTR(+3)	10.3	2	513.893	591.31	14.8	3.06
FEHC[+57.021464]NFNDVTTR (heavy)(+3)	10.3	2	517.229	387.223	14.8	3.06
FEHC[+57.021464]NFNDVTTR (heavy)(+3)	10.3	2	517.229	486.291	14.8	3.06
FEHC[+57.021464]NFNDVTTR (heavy)(+3)	10.3	2	517.229	532.698	14.8	3.06
FEHC[+57.021464]NFNDVTTR (heavy)(+3)	10.3	2	517.229	601.318	14.8	3.06
AFC[+57.021464]NYDGGDC[+57.021464]C[+57.021464]TSTVK(+2)	10.45	2	927.858	818.805	30.5	2.499
AFC[+57.021464]NYDGGDC[+57.021464]C[+57.021464]TSTVK(+2)	10.45	2	927.858	1084.44	30.5	2.499
AFC[+57.021464]NYDGGDC[+57.021464]C[+57.021464]TSTVK(+2)	10.45	2	927.858	1199.467	30.5	2.499
AFC[+57.021464]NYDGGDC[+57.021464]C[+57.021464]TSTVK(+2)	10.45	2	927.858	1362.53	30.5	2.499
AFC[+57.021464]NYDGGDC[+57.021464]C[+57.021464]TSTVK (heavy)(+2)	10.45	2	931.865	822.813	30.5	2.499
AFC[+57.021464]NYDGGDC[+57.021464]C[+57.021464]TSTVK (heavy)(+2)	10.45	2	931.865	1092.454	30.5	2.499
AFC[+57.021464]NYDGGDC[+57.021464]C[+57.021464]TSTVK (heavy)(+2)	10.45	2	931.865	1207.481	30.5	2.499
AFC[+57.021464]NYDGGDC[+57.021464]C[+57.021464]TSTVK (heavy)(+2)	10.45	2	931.865	1370.544	30.5	2.499
QTHQPPAPNSLIR(+3)	10.49	2	486.931	350.211	14.1	2.499
QTHQPPAPNSLIR(+3)	10.49	2	486.931	602.362	14.1	2.499
QTHQPPAPNSLIR(+3)	10.49	2	486.931	760.374	14.1	2.499
QTHQPPAPNSLIR (heavy)(+3)	10.49	2	490.268	355.215	14.1	2.499
QTHQPPAPNSLIR (heavy)(+3)	10.49	2	490.268	612.37	14.1	2.499
QTHQPPAPNSLIR (heavy)(+3)	10.49	2	490.268	760.374	14.1	2.499
TDAPDLPEENQAR(+2)	10.49	2	728.339	584.783	23.9	2.499
TDAPDLPEENQAR(+2)	10.49	2	728.339	620.302	23.9	2.499
TDAPDLPEENQAR(+2)	10.49	2	728.339	843.396	23.9	2.499
TDAPDLPEENQAR(+2)	10.49	2	728.339	1168.559	23.9	2.499
TDAPDLPEENQAR (heavy)(+2)	10.49	2	733.343	589.787	23.9	2.499
TDAPDLPEENQAR (heavy)(+2)	10.49	2	733.343	625.306	23.9	2.499
TDAPDLPEENQAR (heavy)(+2)	10.49	2	733.343	853.404	23.9	2.499
TDAPDLPEENQAR (heavy)(+2)	10.49	2	733.343	1178.568	23.9	2.499
VLVNEQGHYDAVTGK(+3)	10.54	2	543.945	602.783	15.7	2.499
VLVNEQGHYDAVTGK(+3)	10.54	2	543.945	663.328	15.7	2.499
VLVNEQGHYDAVTGK(+3)	10.54	2	543.945	709.339	15.7	2.499
VLVNEQGHYDAVTGK(+3)	10.54	2	543.945	765.881	15.7	2.499
VLVNEQGHYDAVTGK (heavy)(+3)	10.54	2	546.617	606.79	15.7	2.499
VLVNEQGHYDAVTGK (heavy)(+3)	10.54	2	546.617	663.328	15.7	2.499
VLVNEQGHYDAVTGK (heavy)(+3)	10.54	2	546.617	713.346	15.7	2.499
VLVNEQGHYDAVTGK (heavy)(+3)	10.54	2	546.617	769.888	15.7	2.499
DLQAQVVESAKK (heavy)(+3)	10.56	2	444.588	488.294	10.5	2.499
DLQAQVVESAKK (heavy)(+3)	10.56	2	444.588	578.348	14.5	2.499
DLQAQVVESAKK (heavy)(+3)	10.56	2	444.588	677.416	16.5	2.499
NVIAGIR(+2)	10.7	2	371.732	265.176	12.1	2.331
NVIAGIR(+2)	10.7	2	371.732	327.203	12.1	2.331
NVIAGIR(+2)	10.7	2	371.732	345.224	12.1	2.331

NVIAGIR(+2)	10.7	2	371.732	416.262	12.1	2.331
NVIAGIR(+2)	10.7	2	371.732	529.346	12.1	2.331
NVIAGIR (heavy)(+2)	10.7	2	376.736	270.181	12.1	2.331
NVIAGIR (heavy)(+2)	10.7	2	376.736	327.203	12.1	2.331
NVIAGIR (heavy)(+2)	10.7	2	376.736	355.233	12.1	2.331
NVIAGIR (heavy)(+2)	10.7	2	376.736	426.27	12.1	2.331
NVIAGIR (heavy)(+2)	10.7	2	376.736	539.354	12.1	2.331
SLGFC[+57.021464]DTTNK(+2)	10.72	2	571.76	258.145	19.8	2.331
SLGFC[+57.021464]DTTNK(+2)	10.72	2	571.76	471.703	15.8	2.331
SLGFC[+57.021464]DTTNK(+2)	10.72	2	571.76	738.309	17.8	2.331
SLGFC[+57.021464]DTTNK(+2)	10.72	2	571.76	942.399	17.8	2.331
SLGFC[+57.021464]DTTNK (heavy)(+2)	10.72	2	575.768	258.145	19.8	2.331
SLGFC[+57.021464]DTTNK (heavy)(+2)	10.72	2	575.768	475.71	15.8	2.331
SLGFC[+57.021464]DTTNK (heavy)(+2)	10.72	2	575.768	746.323	17.8	2.331
SLGFC[+57.021464]DTTNK (heavy)(+2)	10.72	2	575.768	950.413	17.8	2.331
MTDQVNYQAMK(+2)	10.88	2	664.802	754.355	21.8	2.274
MTDQVNYQAMK(+2)	10.88	2	664.802	853.424	21.8	2.274
MTDQVNYQAMK(+2)	10.88	2	664.802	981.482	21.8	2.274
MTDQVNYQAMK(+2)	10.88	2	664.802	1096.509	21.8	2.274
MTDQVNYQAMK (heavy)(+2)	10.88	2	668.809	762.369	21.8	2.274
MTDQVNYQAMK (heavy)(+2)	10.88	2	668.809	861.438	21.8	2.274
MTDQVNYQAMK (heavy)(+2)	10.88	2	668.809	989.496	21.8	2.274
MTDQVNYQAMK (heavy)(+2)	10.88	2	668.809	1104.523	21.8	2.274
GFVVAGPSR(+2)	11	2	445.248	304.166	14.6	2.274
GFVVAGPSR(+2)	11	2	445.248	416.225	14.6	2.274
GFVVAGPSR(+2)	11	2	445.248	487.262	14.6	2.274
GFVVAGPSR(+2)	11	2	445.248	586.331	14.6	2.274
GFVVAGPSR(+2)	11	2	445.248	685.399	14.6	2.274
GFVVAGPSR (heavy)(+2)	11	2	450.252	304.166	14.6	2.274
GFVVAGPSR (heavy)(+2)	11	2	450.252	426.233	14.6	2.274
GFVVAGPSR (heavy)(+2)	11	2	450.252	497.271	14.6	2.274
GFVVAGPSR (heavy)(+2)	11	2	450.252	596.339	14.6	2.274
GFVVAGPSR (heavy)(+2)	11	2	450.252	695.407	14.6	2.274
VSEADSSNADWVTK(+2)	11.1	2	754.846	920.447	24.8	2.22
VSEADSSNADWVTK(+2)	11.1	2	754.846	1007.479	24.8	2.22
VSEADSSNADWVTK(+2)	11.1	2	754.846	1122.506	24.8	2.22
VSEADSSNADWVTK(+2)	11.1	2	754.846	1193.543	24.8	2.22
VSEADSSNADWVTK (heavy)(+2)	11.1	2	758.853	928.461	24.8	2.22
VSEADSSNADWVTK (heavy)(+2)	11.1	2	758.853	1015.493	24.8	2.22
VSEADSSNADWVTK (heavy)(+2)	11.1	2	758.853	1130.52	24.8	2.22
VSEADSSNADWVTK (heavy)(+2)	11.1	2	758.853	1201.558	24.8	2.22
ESDTSYVSLK(+2)	11.17	2	564.774	347.229	15.5	2.22
ESDTSYVSLK(+2)	11.17	2	564.774	446.297	16.5	2.22
ESDTSYVSLK(+2)	11.17	2	564.774	609.361	16.5	2.22
ESDTSYVSLK(+2)	11.17	2	564.774	696.393	16.5	2.22
ESDTSYVSLK (heavy)(+2)	11.17	2	568.781	355.243	15.5	2.22
ESDTSYVSLK (heavy)(+2)	11.17	2	568.781	454.312	16.5	2.22
ESDTSYVSLK (heavy)(+2)	11.17	2	568.781	617.375	16.5	2.22
ESDTSYVSLK (heavy)(+2)	11.17	2	568.781	704.407	16.5	2.22
GQYC[+57.021464]YELDEK(+2)	11.17	2	652.776	560.237	20.5	2.22
GQYC[+57.021464]YELDEK(+2)	11.17	2	652.776	796.372	22.5	2.22
GQYC[+57.021464]YELDEK(+2)	11.17	2	652.776	956.403	18.5	2.22
GQYC[+57.021464]YELDEK(+2)	11.17	2	652.776	1119.466	20.5	2.22
GQYC[+57.021464]YELDEK (heavy)(+2)	11.17	2	656.783	564.244	20.5	2.22
GQYC[+57.021464]YELDEK (heavy)(+2)	11.17	2	656.783	804.387	22.5	2.22
GQYC[+57.021464]YELDEK (heavy)(+2)	11.17	2	656.783	964.417	18.5	2.22
GQYC[+57.021464]YELDEK (heavy)(+2)	11.17	2	656.783	1127.481	20.5	2.22
YDVENC[+57.021464]LANK(+2)	11.28	2	613.279	719.35	20.1	2.22
YDVENC[+57.021464]LANK(+2)	11.28	2	613.279	848.393	20.1	2.22
YDVENC[+57.021464]LANK(+2)	11.28	2	613.279	947.461	20.1	2.22
YDVENC[+57.021464]LANK (heavy)(+2)	11.28	2	617.286	727.365	20.1	2.22
YDVENC[+57.021464]LANK (heavy)(+2)	11.28	2	617.286	856.407	20.1	2.22
YDVENC[+57.021464]LANK (heavy)(+2)	11.28	2	617.286	955.476	20.1	2.22
GQVVPALNEK(+2)	11.3	2	527.798	285.156	16.3	2.22
GQVVPALNEK(+2)	11.3	2	527.798	384.224	17.3	2.22
GQVVPALNEK(+2)	11.3	2	527.798	671.372	16.3	2.22
GQVVPALNEK(+2)	11.3	2	527.798	770.441	16.3	2.22
GQVVPALNEK (heavy)(+2)	11.3	2	531.805	285.156	16.3	2.22
GQVVPALNEK (heavy)(+2)	11.3	2	531.805	384.224	17.3	2.22
GQVVPALNEK (heavy)(+2)	11.3	2	531.805	679.386	16.3	2.22
GQVVPALNEK (heavy)(+2)	11.3	2	531.805	778.455	16.3	2.22
VLATLC[+57.021464]GQESTDTER(+2)	11.37	2	840.398	1022.439	28.8	2.08
VLATLC[+57.021464]GQESTDTER(+2)	11.37	2	840.398	1182.469	28.8	2.08
VLATLC[+57.021464]GQESTDTER(+2)	11.37	2	840.398	1295.553	30.8	2.08
VLATLC[+57.021464]GQESTDTER(+2)	11.37	2	840.398	1396.601	28.8	2.08
VLATLC[+57.021464]GQESTDTER (heavy)(+2)	11.37	2	845.403	1032.447	28.8	2.08
VLATLC[+57.021464]GQESTDTER (heavy)(+2)	11.37	2	845.403	1192.477	28.8	2.08

VLATLC[+57.021464]GQESTDTER (heavy)(+2)	11.37	2	845.403	1305.561	30.8	2.08
VLATLC[+57.021464]GQESTDTER (heavy)(+2)	11.37	2	845.403	1406.609	28.8	2.08
YGVSDYYK (heavy)(+2)	11.38	2	501.736	320.16	13.2	2.08
YGVSDYYK (heavy)(+2)	11.38	2	501.736	596.281	15.2	2.08
YGVSDYYK (heavy)(+2)	11.38	2	501.736	683.313	15.2	2.08
LTC[+57.021464]LQNLK(+2)	11.39	2	495.276	388.21	16.2	2.08
LTC[+57.021464]LQNLK(+2)	11.39	2	495.276	438.734	16.2	2.08
LTC[+57.021464]LQNLK(+2)	11.39	2	495.276	615.382	16.2	2.08
LTC[+57.021464]LQNLK(+2)	11.39	2	495.276	775.413	16.2	2.08
LTC[+57.021464]LQNLK (heavy)(+2)	11.39	2	499.283	392.217	16.2	2.08
LTC[+57.021464]LQNLK (heavy)(+2)	11.39	2	499.283	442.741	16.2	2.08
LTC[+57.021464]LQNLK (heavy)(+2)	11.39	2	499.283	623.397	16.2	2.08
LTC[+57.021464]LQNLK (heavy)(+2)	11.39	2	499.283	783.427	16.2	2.08
ATVMFEC[+57.021464]DK(+2)	11.46	2	550.741	464.699	18.1	2.08
ATVMFEC[+57.021464]DK(+2)	11.46	2	550.741	698.281	18.1	2.08
ATVMFEC[+57.021464]DK(+2)	11.46	2	550.741	829.322	18.1	2.08
ATVMFEC[+57.021464]DK(+2)	11.46	2	550.741	928.39	18.1	2.08
ATVMFEC[+57.021464]DK (heavy)(+2)	11.46	2	554.748	468.706	18.1	2.08
ATVMFEC[+57.021464]DK (heavy)(+2)	11.46	2	554.748	706.296	18.1	2.08
ATVMFEC[+57.021464]DK (heavy)(+2)	11.46	2	554.748	837.336	18.1	2.08
ATVMFEC[+57.021464]DK (heavy)(+2)	11.46	2	554.748	936.404	18.1	2.08
DQVLVSC[+57.021464]DTGYK(+2)	11.49	2	692.824	343.161	21.8	2.08
DQVLVSC[+57.021464]DTGYK(+2)	11.49	2	692.824	830.335	19.8	2.08
DQVLVSC[+57.021464]DTGYK(+2)	11.49	2	692.824	929.403	19.8	2.08
DQVLVSC[+57.021464]DTGYK(+2)	11.49	2	692.824	1042.487	21.8	2.08
DQVLVSC[+57.021464]DTGYK (heavy)(+2)	11.49	2	696.831	343.161	21.8	2.08
DQVLVSC[+57.021464]DTGYK (heavy)(+2)	11.49	2	696.831	838.349	19.8	2.08
DQVLVSC[+57.021464]DTGYK (heavy)(+2)	11.49	2	696.831	937.418	19.8	2.08
DQVLVSC[+57.021464]DTGYK (heavy)(+2)	11.49	2	696.831	1050.502	21.8	2.08
YVVISQGLDKPR(+3)	11.58	2	458.929	450.749	13.3	2.08
YVVISQGLDKPR(+3)	11.58	2	458.929	507.291	13.3	2.08
YVVISQGLDKPR(+3)	11.58	2	458.929	556.825	13.3	2.08
YVVISQGLDKPR(+3)	11.58	2	458.929	606.359	13.3	2.08
YVVISQGLDKPR (heavy)(+3)	11.58	2	464.936	459.76	13.3	2.08
YVVISQGLDKPR (heavy)(+3)	11.58	2	464.936	516.302	13.3	2.08
YVVISQGLDKPR (heavy)(+3)	11.58	2	464.936	565.836	13.3	2.08
YVVISQGLDKPR (heavy)(+3)	11.58	2	464.936	615.37	13.3	2.08
LLEVPEGR(+2)	11.74	2	456.761	356.218	15	2.08
LLEVPEGR(+2)	11.74	2	456.761	458.236	15	2.08
LLEVPEGR(+2)	11.74	2	456.761	557.304	15	2.08
LLEVPEGR(+2)	11.74	2	456.761	686.347	15	2.08
LLEVPEGR(+2)	11.74	2	456.761	799.431	15	2.08
LLEVPEGR (heavy)(+2)	11.74	2	461.765	356.218	15	2.08
LLEVPEGR (heavy)(+2)	11.74	2	461.765	468.244	15	2.08
LLEVPEGR (heavy)(+2)	11.74	2	461.765	567.312	15	2.08
LLEVPEGR (heavy)(+2)	11.74	2	461.765	696.355	15	2.08
LLEVPEGR (heavy)(+2)	11.74	2	461.765	809.439	15	2.08
DLQAQVVESAK (heavy)(+2)	11.82	2	598.324	484.269	19.5	2.047
DLQAQVVESAK (heavy)(+2)	11.82	2	598.324	768.434	16.5	2.047
DLQAQVVESAK (heavy)(+2)	11.82	2	598.324	839.471	18.5	2.047
ELDKYGVSDYYK (heavy)(+3)	11.89	2	499.247	342.16	14.3	2.047
ELDKYGVSDYYK (heavy)(+3)	11.89	2	499.247	627.305	14.3	2.047
ELDKYGVSDYYK (heavy)(+3)	11.89	2	499.247	683.313	14.3	2.047
HEALSPFYSER(+3)	11.9	2	445.882	391.194	12.9	2.047
HEALSPFYSER(+3)	11.9	2	445.882	538.262	12.9	2.047
HEALSPFYSER(+3)	11.9	2	445.882	554.257	12.9	2.047
HEALSPFYSER(+3)	11.9	2	445.882	782.383	12.9	2.047
HEALSPFYSER (heavy)(+3)	11.9	2	449.218	401.202	12.9	2.047
HEALSPFYSER (heavy)(+3)	11.9	2	449.218	538.262	12.9	2.047
HEALSPFYSER (heavy)(+3)	11.9	2	449.218	564.265	12.9	2.047
HEALSPFYSER (heavy)(+3)	11.9	2	449.218	782.383	12.9	2.047
VHVIFNYK(+2)	11.99	2	510.287	336.203	16.7	2.009
VHVIFNYK(+2)	11.99	2	510.287	460.753	16.7	2.009
VHVIFNYK(+2)	11.99	2	510.287	684.372	16.7	2.009
VHVIFNYK(+2)	11.99	2	510.287	783.44	16.7	2.009
VHVIFNYK (heavy)(+2)	11.99	2	514.294	336.203	16.7	2.009
VHVIFNYK (heavy)(+2)	11.99	2	514.294	464.76	16.7	2.009
VHVIFNYK (heavy)(+2)	11.99	2	514.294	692.386	16.7	2.009
VHVIFNYK (heavy)(+2)	11.99	2	514.294	791.454	16.7	2.009
IAWESPQGQVSR(+2)	12.14	2	679.346	771.411	22.3	2.009
IAWESPQGQVSR(+2)	12.14	2	679.346	858.443	22.3	2.009
IAWESPQGQVSR(+2)	12.14	2	679.346	997.474	22.3	2.009
IAWESPQGQVSR(+2)	12.14	2	679.346	1183.574	22.3	2.009
IAWESPQGQVSR (heavy)(+2)	12.14	2	684.35	781.419	22.3	2.009
IAWESPQGQVSR (heavy)(+2)	12.14	2	684.35	868.451	22.3	2.009
IAWESPQGQVSR (heavy)(+2)	12.14	2	684.35	997.474	22.3	2.009
IAWESPQGQVSR (heavy)(+2)	12.14	2	684.35	1183.574	22.3	2.009

DGWSAQPTC[+57.021464]IK(+2)	12.15	2	631.795	359.135	20.7	2.009
DGWSAQPTC[+57.021464]IK(+2)	12.15	2	631.795	446.167	20.7	2.009
DGWSAQPTC[+57.021464]IK(+2)	12.15	2	631.795	817.424	20.7	2.009
DGWSAQPTC[+57.021464]IK(+2)	12.15	2	631.795	904.456	20.7	2.009
DGWSAQPTC[+57.021464]IK (heavy)(+2)	12.15	2	635.802	359.135	20.7	2.009
DGWSAQPTC[+57.021464]IK (heavy)(+2)	12.15	2	635.802	446.167	20.7	2.009
DGWSAQPTC[+57.021464]IK (heavy)(+2)	12.15	2	635.802	825.438	20.7	2.009
DGWSAQPTC[+57.021464]IK (heavy)(+2)	12.15	2	635.802	912.47	20.7	2.009
AGPPGPNGAPGEPQPC[+57.021464]LTGPR(+3)	12.27	2	676.33	400.708	19.4	1.914
AGPPGPNGAPGEPQPC[+57.021464]LTGPR(+3)	12.27	2	676.33	513.263	19.4	1.914
AGPPGPNGAPGEPQPC[+57.021464]LTGPR(+3)	12.27	2	676.33	800.408	19.4	1.914
AGPPGPNGAPGEPQPC[+57.021464]LTGPR(+3)	12.27	2	676.33	1002.464	19.4	1.914
AGPPGPNGAPGEPQPC[+57.021464]LTGPR (heavy)(+3)	12.27	2	679.666	405.712	19.4	1.914
AGPPGPNGAPGEPQPC[+57.021464]LTGPR (heavy)(+3)	12.27	2	679.666	518.268	19.4	1.914
AGPPGPNGAPGEPQPC[+57.021464]LTGPR (heavy)(+3)	12.27	2	679.666	810.417	19.4	1.914
AGPPGPNGAPGEPQPC[+57.021464]LTGPR (heavy)(+3)	12.27	2	679.666	1002.464	19.4	1.914
TINVPLR(+2)	12.28	2	406.753	329.182	13.3	1.914
TINVPLR(+2)	12.28	2	406.753	484.324	14.1	1.914
TINVPLR(+2)	12.28	2	406.753	598.367	14.1	1.914
TINVPLR (heavy)(+2)	12.28	2	411.757	329.182	13.3	1.914
TINVPLR (heavy)(+2)	12.28	2	411.757	494.332	14.1	1.914
TINVPLR (heavy)(+2)	12.28	2	411.757	608.375	14.1	1.914
EHLNQVLLEGER(+3)	12.29	2	479.587	361.185	13.9	1.914
EHLNQVLLEGER(+3)	12.29	2	479.587	490.226	13.9	1.914
EHLNQVLLEGER(+3)	12.29	2	479.587	603.31	13.9	1.914
EHLNQVLLEGER(+3)	12.29	2	479.587	716.394	13.9	1.914
EHLNQVLLEGER (heavy)(+3)	12.29	2	482.924	361.185	13.9	1.914
EHLNQVLLEGER (heavy)(+3)	12.29	2	482.924	500.234	13.9	1.914
EHLNQVLLEGER (heavy)(+3)	12.29	2	482.924	613.318	13.9	1.914
EHLNQVLLEGER (heavy)(+3)	12.29	2	482.924	726.402	13.9	1.914
FDHVITNMNNNYEPR(+3)	12.3	2	621.952	401.214	13.9	1.914
FDHVITNMNNNYEPR(+3)	12.3	2	621.952	564.278	17.9	1.914
FDHVITNMNNNYEPR(+3)	12.3	2	621.952	678.321	13.9	1.914
FDHVITNMNNNYEPR(+3)	12.3	2	621.952	683.314	13.9	1.914
FDHVITNMNNNYEPR(+3)	12.3	2	621.952	906.406	17.9	1.914
FDHVITNMNNNYEPR (heavy)(+3)	12.3	2	625.289	411.223	13.9	1.914
FDHVITNMNNNYEPR (heavy)(+3)	12.3	2	625.289	574.286	17.9	1.914
FDHVITNMNNNYEPR (heavy)(+3)	12.3	2	625.289	688.319	13.9	1.914
FDHVITNMNNNYEPR (heavy)(+3)	12.3	2	625.289	688.329	13.9	1.914
FDHVITNMNNNYEPR (heavy)(+3)	12.3	2	625.289	916.415	17.9	1.914
LPVAPLR(+2)	12.31	2	383.252	326.711	12.5	1.914
LPVAPLR(+2)	12.31	2	383.252	456.293	12.5	1.914
LPVAPLR(+2)	12.31	2	383.252	555.361	12.5	1.914
LPVAPLR(+2)	12.31	2	383.252	652.414	12.5	1.914
LPVAPLR (heavy)(+2)	12.31	2	388.256	331.715	12.5	1.914
LPVAPLR (heavy)(+2)	12.31	2	388.256	466.301	12.5	1.914
LPVAPLR (heavy)(+2)	12.31	2	388.256	565.37	12.5	1.914
LPVAPLR (heavy)(+2)	12.31	2	388.256	662.422	12.5	1.914
YEFLNGR(+2)	12.37	2	449.724	303.672	14.7	1.914
YEFLNGR(+2)	12.37	2	449.724	346.183	14.7	1.914
YEFLNGR(+2)	12.37	2	449.724	459.267	14.7	1.914
YEFLNGR(+2)	12.37	2	449.724	606.336	14.7	1.914
YEFLNGR (heavy)(+2)	12.37	2	454.728	308.676	14.7	1.914
YEFLNGR (heavy)(+2)	12.37	2	454.728	356.192	14.7	1.914
YEFLNGR (heavy)(+2)	12.37	2	454.728	469.276	14.7	1.914
YEFLNGR (heavy)(+2)	12.37	2	454.728	616.344	14.7	1.914
VLGPFC[+57.021464]GEK(+2)	12.38	2	503.754	397.679	15.4	1.914
VLGPFC[+57.021464]GEK(+2)	12.38	2	503.754	454.221	16.5	1.914
VLGPFC[+57.021464]GEK(+2)	12.38	2	503.754	737.329	17.4	1.914
VLGPFC[+57.021464]GEK(+2)	12.38	2	503.754	794.35	15.4	1.914
VLGPFC[+57.021464]GEK(+2)	12.38	2	503.754	907.434	17.4	1.914
VLGPFC[+57.021464]GEK (heavy)(+2)	12.38	2	507.762	401.686	15.4	1.914
VLGPFC[+57.021464]GEK (heavy)(+2)	12.38	2	507.762	458.228	16.5	1.914
VLGPFC[+57.021464]GEK (heavy)(+2)	12.38	2	507.762	745.343	17.4	1.914
VLGPFC[+57.021464]GEK (heavy)(+2)	12.38	2	507.762	802.364	15.4	1.914
VLGPFC[+57.021464]GEK (heavy)(+2)	12.38	2	507.762	915.448	17.4	1.914
MALEVYK(+2)	12.39	2	427.228	316.169	14	1.914
MALEVYK(+2)	12.39	2	427.228	538.287	14	1.914
MALEVYK(+2)	12.39	2	427.228	651.371	14	1.914
MALEVYK (heavy)(+2)	12.39	2	431.235	316.169	14	1.914
MALEVYK (heavy)(+2)	12.39	2	431.235	546.301	14	1.914
MALEVYK (heavy)(+2)	12.39	2	431.235	659.385	14	1.914
VGYVSGWGR(+2)	12.53	2	490.751	320.16	16.1	1.914
VGYVSGWGR(+2)	12.53	2	490.751	475.241	16.1	1.914
VGYVSGWGR(+2)	12.53	2	490.751	562.273	16.1	1.914
VGYVSGWGR(+2)	12.53	2	490.751	661.342	16.1	1.914
VGYVSGWGR (heavy)(+2)	12.53	2	495.755	320.16	16.1	1.914

VGYVSGWGR (heavy)(+2)	12.53	2	495.755	485.249	16.1	1.914
VGYVSGWGR (heavy)(+2)	12.53	2	495.755	572.281	16.1	1.914
VGYVSGWGR (heavy)(+2)	12.53	2	495.755	671.35	16.1	1.914
GMC[+57.021464]TSPPLIK(+2)	12.64	2	552.283	458.252	18.1	1.914
GMC[+57.021464]TSPPLIK(+2)	12.64	2	552.283	567.386	18.1	1.914
GMC[+57.021464]TSPPLIK(+2)	12.64	2	552.283	755.466	18.1	1.914
GMC[+57.021464]TSPPLIK(+2)	12.64	2	552.283	915.497	18.1	1.914
GMC[+57.021464]TSPPLIK (heavy)(+2)	12.64	2	556.29	462.259	18.1	1.914
GMC[+57.021464]TSPPLIK (heavy)(+2)	12.64	2	556.29	575.401	18.1	1.914
GMC[+57.021464]TSPPLIK (heavy)(+2)	12.64	2	556.29	763.48	18.1	1.914
GMC[+57.021464]TSPPLIK (heavy)(+2)	12.64	2	556.29	923.511	18.1	1.914
GTWSGPAPLC[+57.021464]K(+2)	12.65	2	587.289	685.37	19.3	1.914
GTWSGPAPLC[+57.021464]K(+2)	12.65	2	587.289	742.392	19.3	1.914
GTWSGPAPLC[+57.021464]K(+2)	12.65	2	587.289	829.424	19.3	1.914
GTWSGPAPLC[+57.021464]K (heavy)(+2)	12.65	2	591.296	693.384	19.3	1.914
GTWSGPAPLC[+57.021464]K (heavy)(+2)	12.65	2	591.296	750.406	19.3	1.914
GTWSGPAPLC[+57.021464]K (heavy)(+2)	12.65	2	591.296	837.438	19.3	1.914
EAQLPVIENK(+2)	12.66	2	570.816	329.146	18.7	1.914
EAQLPVIENK(+2)	12.66	2	570.816	442.23	18.7	1.914
EAQLPVIENK(+2)	12.66	2	570.816	699.404	18.7	1.914
EAQLPVIENK(+2)	12.66	2	570.816	812.488	18.7	1.914
EAQLPVIENK (heavy)(+2)	12.66	2	574.823	329.146	18.7	1.914
EAQLPVIENK (heavy)(+2)	12.66	2	574.823	442.23	18.7	1.914
EAQLPVIENK (heavy)(+2)	12.66	2	574.823	707.418	18.7	1.914
EAQLPVIENK (heavy)(+2)	12.66	2	574.823	820.502	18.7	1.914
SIDVAC[+57.021464]HPGYALPK(+3)	12.68	2	509.925	316.15	14.7	1.914
SIDVAC[+57.021464]HPGYALPK(+3)	12.68	2	509.925	557.279	14.7	1.914
SIDVAC[+57.021464]HPGYALPK(+3)	12.68	2	509.925	606.813	14.7	1.914
SIDVAC[+57.021464]HPGYALPK(+3)	12.68	2	509.925	664.327	14.7	1.914
SIDVAC[+57.021464]HPGYALPK (heavy)(+3)	12.68	2	512.597	316.15	14.7	1.914
SIDVAC[+57.021464]HPGYALPK (heavy)(+3)	12.68	2	512.597	561.286	14.7	1.914
SIDVAC[+57.021464]HPGYALPK (heavy)(+3)	12.68	2	512.597	610.82	14.7	1.914
SIDVAC[+57.021464]HPGYALPK (heavy)(+3)	12.68	2	512.597	668.334	14.7	1.914
VFC[+57.021464]QPWQR(+2)	12.68	2	560.771	437.703	15.4	1.914
VFC[+57.021464]QPWQR(+2)	12.68	2	560.771	586.31	21.4	1.914
VFC[+57.021464]QPWQR(+2)	12.68	2	560.771	714.368	17.4	1.914
VFC[+57.021464]QPWQR(+2)	12.68	2	560.771	874.399	19.4	1.914
VFC[+57.021464]QPWQR (heavy)(+2)	12.68	2	565.775	442.707	15.4	1.914
VFC[+57.021464]QPWQR (heavy)(+2)	12.68	2	565.775	596.318	21.4	1.914
VFC[+57.021464]QPWQR (heavy)(+2)	12.68	2	565.775	724.376	17.4	1.914
VFC[+57.021464]QPWQR (heavy)(+2)	12.68	2	565.775	884.407	19.4	1.914
C[+57.021464]YSGFTLK(+2)	12.71	2	488.233	411.133	16	1.914
C[+57.021464]YSGFTLK(+2)	12.71	2	488.233	565.334	16	1.914
C[+57.021464]YSGFTLK(+2)	12.71	2	488.233	652.366	16	1.914
C[+57.021464]YSGFTLK (heavy)(+2)	12.71	2	492.24	411.133	16	1.914
C[+57.021464]YSGFTLK (heavy)(+2)	12.71	2	492.24	573.349	16	1.914
C[+57.021464]YSGFTLK (heavy)(+2)	12.71	2	492.24	660.381	16	1.914
LLDSLPSDTR(+2)	12.85	2	558.798	429.234	17.3	1.914
LLDSLPSDTR(+2)	12.85	2	558.798	575.278	17.3	1.914
LLDSLPSDTR(+2)	12.85	2	558.798	775.394	21.3	1.914
LLDSLPSDTR(+2)	12.85	2	558.798	890.421	17.3	1.914
LLDSLPSDTR (heavy)(+2)	12.85	2	563.802	429.234	17.3	1.914
LLDSLPSDTR (heavy)(+2)	12.85	2	563.802	585.287	17.3	1.914
LLDSLPSDTR (heavy)(+2)	12.85	2	563.802	785.403	21.3	1.914
LLDSLPSDTR (heavy)(+2)	12.85	2	563.802	900.43	17.3	1.914
FQWVDGSR(+2)	12.86	2	497.74	319.172	16.3	1.914
FQWVDGSR(+2)	12.86	2	497.74	462.214	16.3	1.914
FQWVDGSR(+2)	12.86	2	497.74	533.268	16.3	1.914
FQWVDGSR(+2)	12.86	2	497.74	719.347	16.3	1.914
FQWVDGSR (heavy)(+2)	12.86	2	502.744	329.181	16.3	1.914
FQWVDGSR (heavy)(+2)	12.86	2	502.744	462.214	16.3	1.914
FQWVDGSR (heavy)(+2)	12.86	2	502.744	543.276	16.3	1.914
FQWVDGSR (heavy)(+2)	12.86	2	502.744	729.355	16.3	1.914
GDEVWLR(+2)	12.97	2	437.724	474.282	14.3	1.914
GDEVWLR(+2)	12.97	2	437.724	573.351	14.3	1.914
GDEVWLR(+2)	12.97	2	437.724	702.393	14.3	1.914
GDEVWLR (heavy)(+2)	12.97	2	442.728	484.291	14.3	1.914
GDEVWLR (heavy)(+2)	12.97	2	442.728	583.359	14.3	1.914
GDEVWLR (heavy)(+2)	12.97	2	442.728	712.402	14.3	1.914
INYTVPQSGTFK(+2)	13.04	2	677.853	764.394	22.3	1.914
INYTVPQSGTFK(+2)	13.04	2	677.853	863.462	22.3	1.914
INYTVPQSGTFK(+2)	13.04	2	677.853	964.51	22.3	1.914
INYTVPQSGTFK(+2)	13.04	2	677.853	1127.573	22.3	1.914
INYTVPQSGTFK (heavy)(+2)	13.04	2	681.86	772.408	22.3	1.914
INYTVPQSGTFK (heavy)(+2)	13.04	2	681.86	871.476	22.3	1.914
INYTVPQSGTFK (heavy)(+2)	13.04	2	681.86	972.524	22.3	1.914
INYTVPQSGTFK (heavy)(+2)	13.04	2	681.86	1135.587	22.3	1.914

EATLQDC[+57.021464]PSGPWGK(+2)	13.08	2	773.353	728.373	24.6	1.914
EATLQDC[+57.021464]PSGPWGK(+2)	13.08	2	773.353	888.403	28.6	1.914
EATLQDC[+57.021464]PSGPWGK(+2)	13.08	2	773.353	1131.489	24.6	1.914
EATLQDC[+57.021464]PSGPWGK(+2)	13.08	2	773.353	1244.573	25.4	1.914
EATLQDC[+57.021464]PSGPWGK (heavy)(+2)	13.08	2	777.36	736.387	24.6	1.914
EATLQDC[+57.021464]PSGPWGK (heavy)(+2)	13.08	2	777.36	896.417	28.6	1.914
EATLQDC[+57.021464]PSGPWGK (heavy)(+2)	13.08	2	777.36	1139.503	24.6	1.914
EATLQDC[+57.021464]PSGPWGK (heavy)(+2)	13.08	2	777.36	1252.587	25.4	1.914
IGESIELTC[+57.021464]PK(+2)	13.17	2	623.821	505.244	20.5	1.914
IGESIELTC[+57.021464]PK(+2)	13.17	2	623.821	618.328	20.5	1.914
IGESIELTC[+57.021464]PK(+2)	13.17	2	623.821	747.371	20.5	1.914
IGESIELTC[+57.021464]PK(+2)	13.17	2	623.821	947.487	20.5	1.914
IGESIELTC[+57.021464]PK(+2)	13.17	2	623.821	1133.551	20.5	1.914
IGESIELTC[+57.021464]PK (heavy)(+2)	13.17	2	627.828	513.258	20.5	1.914
IGESIELTC[+57.021464]PK (heavy)(+2)	13.17	2	627.828	626.342	20.5	1.914
IGESIELTC[+57.021464]PK (heavy)(+2)	13.17	2	627.828	755.385	20.5	1.914
IGESIELTC[+57.021464]PK (heavy)(+2)	13.17	2	627.828	955.501	20.5	1.914
IGESIELTC[+57.021464]PK (heavy)(+2)	13.17	2	627.828	1141.565	20.5	1.914
SLSQQIENIR(+2)	13.27	2	594.322	494.265	19.5	1.914
SLSQQIENIR(+2)	13.27	2	594.322	644.373	19.5	1.914
SLSQQIENIR(+2)	13.27	2	594.322	772.431	19.5	1.914
SLSQQIENIR(+2)	13.27	2	594.322	987.522	19.5	1.914
SLSQQIENIR (heavy)(+2)	13.27	2	599.326	499.269	19.5	1.914
SLSQQIENIR (heavy)(+2)	13.27	2	599.326	654.381	19.5	1.914
SLSQQIENIR (heavy)(+2)	13.27	2	599.326	782.439	19.5	1.914
SLSQQIENIR (heavy)(+2)	13.27	2	599.326	997.53	19.5	1.914
TEAPTTMTYPLK(+2)	13.3	2	676.841	302.135	22.2	1.914
TEAPTTMTYPLK(+2)	13.3	2	676.841	526.278	22.2	1.914
TEAPTTMTYPLK(+2)	13.3	2	676.841	561.797	22.2	1.914
TEAPTTMTYPLK(+2)	13.3	2	676.841	1051.549	22.2	1.914
TEAPTTMTYPLK (heavy)(+2)	13.3	2	680.849	302.135	22.2	1.914
TEAPTTMTYPLK (heavy)(+2)	13.3	2	680.849	530.285	22.2	1.914
TEAPTTMTYPLK (heavy)(+2)	13.3	2	680.849	565.804	22.2	1.914
TEAPTTMTYPLK (heavy)(+2)	13.3	2	680.849	1059.563	22.2	1.914
EVSFQSTGESEWK(+2)	13.3	2	757.343	923.411	24.9	1.914
EVSFQSTGESEWK(+2)	13.3	2	757.343	1051.469	24.9	1.914
EVSFQSTGESEWK(+2)	13.3	2	757.343	1198.537	24.9	1.914
EVSFQSTGESEWK(+2)	13.3	2	757.343	1285.57	24.9	1.914
EVSFQSTGESEWK (heavy)(+2)	13.3	2	761.351	931.425	24.9	1.914
EVSFQSTGESEWK (heavy)(+2)	13.3	2	761.351	1059.483	24.9	1.914
EVSFQSTGESEWK (heavy)(+2)	13.3	2	761.351	1206.552	24.9	1.914
EVSFQSTGESEWK (heavy)(+2)	13.3	2	761.351	1293.584	24.9	1.914
DLEVDTTLK(+2)	13.33	2	517.274	358.161	17	1.914
DLEVDTTLK(+2)	13.33	2	517.274	462.292	17	1.914
DLEVDTTLK(+2)	13.33	2	517.274	577.319	17	1.914
DLEVDTTLK(+2)	13.33	2	517.274	676.388	17	1.914
DLEVDTTLK(+2)	13.33	2	517.274	805.43	17	1.914
DLEVDTTLK (heavy)(+2)	13.33	2	521.281	358.161	17	1.914
DLEVDTTLK (heavy)(+2)	13.33	2	521.281	470.306	17	1.914
DLEVDTTLK (heavy)(+2)	13.33	2	521.281	585.333	17	1.914
DLEVDTTLK (heavy)(+2)	13.33	2	521.281	684.402	17	1.914
DLEVDTTLK (heavy)(+2)	13.33	2	521.281	813.444	17	1.914
GILNEIK(+2)	13.35	2	393.739	284.197	12.9	1.914
GILNEIK(+2)	13.35	2	393.739	503.282	21.7	1.914
GILNEIK(+2)	13.35	2	393.739	616.366	13.7	1.914
GILNEIK (heavy)(+2)	13.35	2	397.746	284.197	12.9	1.914
GILNEIK (heavy)(+2)	13.35	2	397.746	511.297	21.7	1.914
GILNEIK (heavy)(+2)	13.35	2	397.746	624.381	13.7	1.914
LFLEPTR(+2)	13.41	2	438.252	373.219	14.3	1.914
LFLEPTR(+2)	13.41	2	438.252	502.262	14.3	1.914
LFLEPTR(+2)	13.41	2	438.252	615.346	14.3	1.914
LFLEPTR(+2)	13.41	2	438.252	762.414	14.3	1.914
LFLEPTR (heavy)(+2)	13.41	2	443.257	383.228	14.3	1.914
LFLEPTR (heavy)(+2)	13.41	2	443.257	512.27	14.3	1.914
LFLEPTR (heavy)(+2)	13.41	2	443.257	625.354	14.3	1.914
LFLEPTR (heavy)(+2)	13.41	2	443.257	772.423	14.3	1.914
LASPGFPGEYANDQER(+2)	13.49	2	875.905	573.303	28.8	1.914
LASPGFPGEYANDQER(+2)	13.49	2	875.905	740.329	28.8	1.914
LASPGFPGEYANDQER(+2)	13.49	2	875.905	783.845	28.8	1.914
LASPGFPGEYANDQER(+2)	13.49	2	875.905	1178.507	28.8	1.914
LASPGFPGEYANDQER (heavy)(+2)	13.49	2	880.909	573.303	28.8	1.914
LASPGFPGEYANDQER (heavy)(+2)	13.49	2	880.909	745.333	28.8	1.914
LASPGFPGEYANDQER (heavy)(+2)	13.49	2	880.909	788.849	28.8	1.914
LASPGFPGEYANDQER (heavy)(+2)	13.49	2	880.909	1188.516	28.8	1.914
VMQHQYQVSNLGQR(+3)	13.53	2	563.282	360.199	16.2	1.914
VMQHQYQVSNLGQR(+3)	13.53	2	563.282	458.211	16.2	1.914
VMQHQYQVSNLGQR(+3)	13.53	2	563.282	674.358	16.2	1.914

VMQHQYQVSNLGQR(+3)	13.53	2	563.282	773.426	16.2	1.914
VMQHQYQVSNLGQR (heavy)(+3)	13.53	2	566.618	370.207	16.2	1.914
VMQHQYQVSNLGQR (heavy)(+3)	13.53	2	566.618	458.211	16.2	1.914
VMQHQYQVSNLGQR (heavy)(+3)	13.53	2	566.618	684.366	16.2	1.914
VMQHQYQVSNLGQR (heavy)(+3)	13.53	2	566.618	783.435	16.2	1.914
AMISSPPFR(+2)	13.57	2	503.262	316.169	16.5	1.914
AMISSPPFR(+2)	13.57	2	503.262	516.293	13.4	1.914
AMISSPPFR(+2)	13.57	2	503.262	690.357	15.4	1.914
AMISSPPFR(+2)	13.57	2	503.262	803.441	15.4	1.914
AMISSPPFR (heavy)(+2)	13.57	2	508.267	316.169	16.5	1.914
AMISSPPFR (heavy)(+2)	13.57	2	508.267	526.301	13.4	1.914
AMISSPPFR (heavy)(+2)	13.57	2	508.267	700.365	15.4	1.914
AMISSPPFR (heavy)(+2)	13.57	2	508.267	813.449	15.4	1.914
AFVFPK(+2)	13.63	2	354.707	318.181	11.6	1.914
AFVFPK(+2)	13.63	2	354.707	391.234	11.6	1.914
AFVFPK(+2)	13.63	2	354.707	490.302	11.6	1.914
AFVFPK(+2)	13.63	2	354.707	637.371	11.6	1.914
AFVFPK (heavy)(+2)	13.63	2	358.714	318.181	11.6	1.914
AFVFPK (heavy)(+2)	13.63	2	358.714	399.248	11.6	1.914
AFVFPK (heavy)(+2)	13.63	2	358.714	498.317	11.6	1.914
AFVFPK (heavy)(+2)	13.63	2	358.714	645.385	11.6	1.914
IAFSATR(+2)	13.76	2	383.216	326.674	12.5	1.914
IAFSATR(+2)	13.76	2	383.216	652.341	12.5	1.914
IAFSATR (heavy)(+2)	13.76	2	388.22	331.678	12.5	1.914
IAFSATR (heavy)(+2)	13.76	2	388.22	662.35	12.5	1.914
ARISEATDGLSDFLK (heavy)(+3)	13.76	2	547.625	530.306	15.6	1.914
ARISEATDGLSDFLK (heavy)(+3)	13.76	2	547.625	567.312	15.6	1.914
ARISEATDGLSDFLK (heavy)(+3)	13.76	2	547.625	739.397	15.6	1.914
FNAVLTNPQGDYDTSTGK(+2)	13.78	2	964.455	333.156	31.8	1.914
FNAVLTNPQGDYDTSTGK(+2)	13.78	2	964.455	1168.512	31.8	1.914
FNAVLTNPQGDYDTSTGK(+2)	13.78	2	964.455	1383.602	31.8	1.914
FNAVLTNPQGDYDTSTGK(+2)	13.78	2	964.455	1496.686	31.8	1.914
FNAVLTNPQGDYDTSTGK (heavy)(+2)	13.78	2	968.462	333.156	31.8	1.914
FNAVLTNPQGDYDTSTGK (heavy)(+2)	13.78	2	968.462	1176.526	31.8	1.914
FNAVLTNPQGDYDTSTGK (heavy)(+2)	13.78	2	968.462	1391.616	31.8	1.914
FNAVLTNPQGDYDTSTGK (heavy)(+2)	13.78	2	968.462	1504.701	31.8	1.914
ALLAFQESK(+2)	13.93	2	503.782	411.721	13.5	1.914
ALLAFQESK(+2)	13.93	2	503.782	638.314	16.5	1.914
ALLAFQESK(+2)	13.93	2	503.782	709.352	16.5	1.914
ALLAFQESK(+2)	13.93	2	503.782	822.436	15.5	1.914
ALLAFQESK (heavy)(+2)	13.93	2	507.789	415.729	13.5	1.914
ALLAFQESK (heavy)(+2)	13.93	2	507.789	646.329	16.5	1.914
ALLAFQESK (heavy)(+2)	13.93	2	507.789	717.366	16.5	1.914
ALLAFQESK (heavy)(+2)	13.93	2	507.789	830.45	15.5	1.914
IVQLIQDTR(+2)	13.93	2	543.319	341.218	17.8	1.914
IVQLIQDTR(+2)	13.93	2	543.319	519.252	17.8	1.914
IVQLIQDTR(+2)	13.93	2	543.319	632.336	17.8	1.914
IVQLIQDTR(+2)	13.93	2	543.319	745.42	17.8	1.914
IVQLIQDTR(+2)	13.93	2	543.319	873.479	17.8	1.914
IVQLIQDTR (heavy)(+2)	13.93	2	548.323	341.218	17.8	1.914
IVQLIQDTR (heavy)(+2)	13.93	2	548.323	529.26	17.8	1.914
IVQLIQDTR (heavy)(+2)	13.93	2	548.323	642.344	17.8	1.914
IVQLIQDTR (heavy)(+2)	13.93	2	548.323	755.429	17.8	1.914
IVQLIQDTR (heavy)(+2)	13.93	2	548.323	883.487	17.8	1.914
GNLC[+57.021464]VNLMR(+2)	14	2	538.77	453.238	14.6	1.914
GNLC[+57.021464]VNLMR(+2)	14	2	538.77	632.355	16.6	1.914
GNLC[+57.021464]VNLMR(+2)	14	2	538.77	792.385	18.6	1.914
GNLC[+57.021464]VNLMR(+2)	14	2	538.77	905.47	17.7	1.914
GNLC[+57.021464]VNLMR (heavy)(+2)	14	2	543.774	458.243	14.6	1.914
GNLC[+57.021464]VNLMR (heavy)(+2)	14	2	543.774	642.363	16.6	1.914
GNLC[+57.021464]VNLMR (heavy)(+2)	14	2	543.774	802.394	18.6	1.914
GNLC[+57.021464]VNLMR (heavy)(+2)	14	2	543.774	915.478	17.7	1.914
LGPISADSTTAPLEK(+2)	14.18	2	750.401	486.292	24.7	1.914
LGPISADSTTAPLEK(+2)	14.18	2	750.401	665.348	24.7	1.914
LGPISADSTTAPLEK(+2)	14.18	2	750.401	1032.521	24.7	1.914
LGPISADSTTAPLEK(+2)	14.18	2	750.401	1119.553	24.7	1.914
LGPISADSTTAPLEK (heavy)(+2)	14.18	2	754.408	494.306	24.7	1.914
LGPISADSTTAPLEK (heavy)(+2)	14.18	2	754.408	669.356	24.7	1.914
LGPISADSTTAPLEK (heavy)(+2)	14.18	2	754.408	1040.535	24.7	1.914
LGPISADSTTAPLEK (heavy)(+2)	14.18	2	754.408	1127.567	24.7	1.914
ITHYNYLILSK(+3)	14.22	2	455.59	460.313	13.2	1.914
ITHYNYLILSK(+3)	14.22	2	455.59	573.397	13.2	1.914
ITHYNYLILSK(+3)	14.22	2	455.59	626.34	13.2	1.914
ITHYNYLILSK(+3)	14.22	2	455.59	792.368	14.2	1.914
ITHYNYLILSK (heavy)(+3)	14.22	2	458.262	468.327	13.2	1.914
ITHYNYLILSK (heavy)(+3)	14.22	2	458.262	581.411	13.2	1.914
ITHYNYLILSK (heavy)(+3)	14.22	2	458.262	630.347	13.2	1.914

ITHYNYLILSK (heavy)(+3)	14.22	2	458.262	792.368	14.2	1.914
AQTTVTC[+57.021464]MENGWSPTPR(+2)	14.25	2	968.438	470.272	31.9	1.914
AQTTVTC[+57.021464]MENGWSPTPR(+2)	14.25	2	968.438	557.304	31.9	1.914
AQTTVTC[+57.021464]MENGWSPTPR(+2)	14.25	2	968.438	1334.562	31.9	1.914
AQTTVTC[+57.021464]MENGWSPTPR(+2)	14.25	2	968.438	1435.609	31.9	1.914
AQTTVTC[+57.021464]MENGWSPTPR (heavy)(+2)	14.25	2	973.442	480.28	31.9	1.914
AQTTVTC[+57.021464]MENGWSPTPR (heavy)(+2)	14.25	2	973.442	567.312	31.9	1.914
AQTTVTC[+57.021464]MENGWSPTPR (heavy)(+2)	14.25	2	973.442	1344.57	31.9	1.914
AQTTVTC[+57.021464]MENGWSPTPR (heavy)(+2)	14.25	2	973.442	1445.618	31.9	1.914
LC[+57.021464]TPLLPK(+2)	14.36	2	471.278	414.736	15.4	2.284
LC[+57.021464]TPLLPK(+2)	14.36	2	471.278	567.386	15.4	2.284
LC[+57.021464]TPLLPK(+2)	14.36	2	471.278	668.434	15.4	2.284
LC[+57.021464]TPLLPK(+2)	14.36	2	471.278	828.465	15.4	2.284
LC[+57.021464]TPLLPK (heavy)(+2)	14.36	2	475.285	418.743	15.4	2.284
LC[+57.021464]TPLLPK (heavy)(+2)	14.36	2	475.285	575.401	15.4	2.284
LC[+57.021464]TPLLPK (heavy)(+2)	14.36	2	475.285	676.448	15.4	2.284
LC[+57.021464]TPLLPK (heavy)(+2)	14.36	2	475.285	836.479	15.4	2.284
VLMSVLNDNSR(+2)	14.38	2	624.324	605.264	20.5	2.391
VLMSVLNDNSR(+2)	14.38	2	624.324	718.348	20.5	2.391
VLMSVLNDNSR(+2)	14.38	2	624.324	904.448	20.5	2.391
VLMSVLNDNSR(+2)	14.38	2	624.324	1035.489	20.5	2.391
VLMSVLNDNSR (heavy)(+2)	14.38	2	629.328	615.272	20.5	2.391
VLMSVLNDNSR (heavy)(+2)	14.38	2	629.328	728.356	20.5	2.391
VLMSVLNDNSR (heavy)(+2)	14.38	2	629.328	914.457	20.5	2.391
VLMSVLNDNSR (heavy)(+2)	14.38	2	629.328	1045.497	20.5	2.391
VDLSFSPSQSLPASHAHLR(+3)	14.52	2	683.688	700.873	19.6	2.391
VDLSFSPSQSLPASHAHLR(+3)	14.52	2	683.688	744.389	19.6	2.391
VDLSFSPSQSLPASHAHLR(+3)	14.52	2	683.688	861.44	19.6	2.391
VDLSFSPSQSLPASHAHLR(+3)	14.52	2	683.688	888.48	19.6	2.391
VDLSFSPSQSLPASHAHLR (heavy)(+3)	14.52	2	687.024	705.877	19.6	2.391
VDLSFSPSQSLPASHAHLR (heavy)(+3)	14.52	2	687.024	749.393	19.6	2.391
VDLSFSPSQSLPASHAHLR (heavy)(+3)	14.52	2	687.024	866.444	19.6	2.391
VDLSFSPSQSLPASHAHLR (heavy)(+3)	14.52	2	687.024	898.488	19.6	2.391
YGLVTYATYPK(+2)	14.85	2	638.334	334.176	21	3.275
YGLVTYATYPK(+2)	14.85	2	638.334	742.377	21	3.275
YGLVTYATYPK(+2)	14.85	2	638.334	843.425	21	3.275
YGLVTYATYPK(+2)	14.85	2	638.334	942.493	21	3.275
YGLVTYATYPK (heavy)(+2)	14.85	2	642.341	334.176	21	3.275
YGLVTYATYPK (heavy)(+2)	14.85	2	642.341	750.391	21	3.275
YGLVTYATYPK (heavy)(+2)	14.85	2	642.341	851.439	21	3.275
YGLVTYATYPK (heavy)(+2)	14.85	2	642.341	950.507	21	3.275
TSFPEDTVITYK(+2)	15.04	2	700.85	533.277	23	3.275
TSFPEDTVITYK(+2)	15.04	2	700.85	606.811	23	3.275
TSFPEDTVITYK(+2)	15.04	2	700.85	1065.546	23	3.275
TSFPEDTVITYK (heavy)(+2)	15.04	2	704.857	537.284	23	3.275
TSFPEDTVITYK (heavy)(+2)	15.04	2	704.857	610.818	23	3.275
TSFPEDTVITYK (heavy)(+2)	15.04	2	704.857	1073.56	23	3.275
FQSVFTVTR(+2)	15.1	2	542.792	363.166	17.8	3.275
FQSVFTVTR(+2)	15.1	2	542.792	476.283	17.8	3.275
FQSVFTVTR(+2)	15.1	2	542.792	623.351	17.8	3.275
FQSVFTVTR(+2)	15.1	2	542.792	722.42	17.8	3.275
FQSVFTVTR (heavy)(+2)	15.1	2	547.797	363.166	17.8	3.275
FQSVFTVTR (heavy)(+2)	15.1	2	547.797	486.291	17.8	3.275
FQSVFTVTR (heavy)(+2)	15.1	2	547.797	633.359	17.8	3.275
FQSVFTVTR (heavy)(+2)	15.1	2	547.797	732.428	17.8	3.275
TQTNASDFPIGTSLK(+2)	15.15	2	790.401	715.435	26	3.275
TQTNASDFPIGTSLK(+2)	15.15	2	790.401	1064.562	26	3.275
TQTNASDFPIGTSLK(+2)	15.15	2	790.401	1249.642	26	3.275
TQTNASDFPIGTSLK(+2)	15.15	2	790.401	1350.69	26	3.275
TQTNASDFPIGTSLK (heavy)(+2)	15.15	2	794.408	723.449	26	3.275
TQTNASDFPIGTSLK (heavy)(+2)	15.15	2	794.408	1072.576	26	3.275
TQTNASDFPIGTSLK (heavy)(+2)	15.15	2	794.408	1257.656	26	3.275
TQTNASDFPIGTSLK (heavy)(+2)	15.15	2	794.408	1358.704	26	3.275
IDNISLTVNDVR(+2)	15.16	2	679.867	343.161	22.3	3.275
IDNISLTVNDVR(+2)	15.16	2	679.867	703.373	22.3	3.275
IDNISLTVNDVR(+2)	15.16	2	679.867	816.457	22.3	3.275
IDNISLTVNDVR(+2)	15.16	2	679.867	903.489	22.3	3.275
IDNISLTVNDVR (heavy)(+2)	15.16	2	684.871	343.161	22.3	3.275
IDNISLTVNDVR (heavy)(+2)	15.16	2	684.871	713.382	22.3	3.275
IDNISLTVNDVR (heavy)(+2)	15.16	2	684.871	826.466	22.3	3.275
IDNISLTVNDVR (heavy)(+2)	15.16	2	684.871	913.498	22.3	3.275
TGDIVEFVC[+57.021464]K(+2)	15.2	2	584.289	387.187	16.2	3.317
TGDIVEFVC[+57.021464]K(+2)	15.2	2	584.289	553.28	17.2	3.317
TGDIVEFVC[+57.021464]K(+2)	15.2	2	584.289	682.323	18.2	3.317
TGDIVEFVC[+57.021464]K(+2)	15.2	2	584.289	781.391	18.2	3.317
TGDIVEFVC[+57.021464]K (heavy)(+2)	15.2	2	588.296	387.187	16.2	3.317
TGDIVEFVC[+57.021464]K (heavy)(+2)	15.2	2	588.296	561.294	17.2	3.317

TGDIVEFVC[+57.021464]K (heavy)(+2)	15.2	2	588.296	690.337	18.2	3.317
TGDIVEFVC[+57.021464]K (heavy)(+2)	15.2	2	588.296	789.405	18.2	3.317
VDLVDFEGNHQFAK(+3)	15.41	2	540.266	596.773	15.6	3.455
VDLVDFEGNHQFAK(+3)	15.41	2	540.266	646.307	15.6	3.455
VDLVDFEGNHQFAK(+3)	15.41	2	540.266	702.849	15.6	3.455
VDLVDFEGNHQFAK(+3)	15.41	2	540.266	760.362	15.6	3.455
VDLVDFEGNHQFAK (heavy)(+3)	15.41	2	542.938	600.78	15.6	3.455
VDLVDFEGNHQFAK (heavy)(+3)	15.41	2	542.938	650.314	15.6	3.455
VDLVDFEGNHQFAK (heavy)(+3)	15.41	2	542.938	706.856	15.6	3.455
VDLVDFEGNHQFAK (heavy)(+3)	15.41	2	542.938	764.37	15.6	3.455
TNFDNDIALVR(+2)	15.42	2	639.327	458.309	21	3.455
TNFDNDIALVR(+2)	15.42	2	639.327	571.393	21	3.455
TNFDNDIALVR(+2)	15.42	2	639.327	915.489	21	3.455
TNFDNDIALVR(+2)	15.42	2	639.327	1062.558	21	3.455
TNFDNDIALVR (heavy)(+2)	15.42	2	644.331	468.317	21	3.455
TNFDNDIALVR (heavy)(+2)	15.42	2	644.331	581.401	21	3.455
TNFDNDIALVR (heavy)(+2)	15.42	2	644.331	925.498	21	3.455
TNFDNDIALVR (heavy)(+2)	15.42	2	644.331	1072.566	21	3.455
VPPPSDAPLPFDR(+2)	15.5	2	704.366	606.306	23.2	3.455
VPPPSDAPLPFDR(+2)	15.5	2	704.366	654.833	23.2	3.455
VPPPSDAPLPFDR(+2)	15.5	2	704.366	1114.553	23.2	3.455
VPPPSDAPLPFDR(+2)	15.5	2	704.366	1211.606	23.2	3.455
VPPPSDAPLPFDR (heavy)(+2)	15.5	2	709.371	611.311	23.2	3.455
VPPPSDAPLPFDR (heavy)(+2)	15.5	2	709.371	659.837	23.2	3.455
VPPPSDAPLPFDR (heavy)(+2)	15.5	2	709.371	1124.561	23.2	3.455
VPPPSDAPLPFDR (heavy)(+2)	15.5	2	709.371	1221.614	23.2	3.455
ADNTWDPEIPVC[+57.021464]EK(+2)	15.51	2	837.377	703.268	27.6	3.455
ADNTWDPEIPVC[+57.021464]EK(+2)	15.51	2	837.377	971.487	27.6	3.455
ADNTWDPEIPVC[+57.021464]EK(+2)	15.51	2	837.377	1086.514	27.6	3.455
ADNTWDPEIPVC[+57.021464]EK (heavy)(+2)	15.51	2	841.384	703.268	27.6	3.455
ADNTWDPEIPVC[+57.021464]EK (heavy)(+2)	15.51	2	841.384	979.501	27.6	3.455
ADNTWDPEIPVC[+57.021464]EK (heavy)(+2)	15.51	2	841.384	1094.528	27.6	3.455
DIPHWLNPTR(+2)	15.52	2	624.827	462.246	20.5	3.455
DIPHWLNPTR(+2)	15.52	2	624.827	510.772	20.5	3.455
DIPHWLNPTR(+2)	15.52	2	624.827	600.346	20.5	3.455
DIPHWLNPTR(+2)	15.52	2	624.827	786.426	20.5	3.455
DIPHWLNPTR(+2)	15.52	2	624.827	1020.537	20.5	3.455
DIPHWLNPTR (heavy)(+2)	15.52	2	629.831	467.25	20.5	3.455
DIPHWLNPTR (heavy)(+2)	15.52	2	629.831	515.776	20.5	3.455
DIPHWLNPTR (heavy)(+2)	15.52	2	629.831	610.355	20.5	3.455
DIPHWLNPTR (heavy)(+2)	15.52	2	629.831	796.434	20.5	3.455
DIPHWLNPTR (heavy)(+2)	15.52	2	629.831	1030.546	20.5	3.455
GSQWSDIEEFC[+57.021464]NR(+2)	15.52	2	814.343	854.346	26.8	3.455
GSQWSDIEEFC[+57.021464]NR(+2)	15.52	2	814.343	1082.457	26.8	3.455
GSQWSDIEEFC[+57.021464]NR(+2)	15.52	2	814.343	1179.495	26.8	3.455
GSQWSDIEEFC[+57.021464]NR(+2)	15.52	2	814.343	1355.568	26.8	3.455
GSQWSDIEEFC[+57.021464]NR (heavy)(+2)	15.52	2	819.348	864.354	26.8	3.455
GSQWSDIEEFC[+57.021464]NR (heavy)(+2)	15.52	2	819.348	1092.465	26.8	3.455
GSQWSDIEEFC[+57.021464]NR (heavy)(+2)	15.52	2	819.348	1179.495	26.8	3.455
GSQWSDIEEFC[+57.021464]NR (heavy)(+2)	15.52	2	819.348	1365.577	26.8	3.455
LPSHSDFLAELR(+3)	15.53	2	462.245	417.246	13.4	3.455
LPSHSDFLAELR(+3)	15.53	2	462.245	544.28	13.4	3.455
LPSHSDFLAELR(+3)	15.53	2	462.245	549.267	13.4	3.455
LPSHSDFLAELR(+3)	15.53	2	462.245	636.323	13.4	3.455
LPSHSDFLAELR(+3)	15.53	2	462.245	784.362	13.4	3.455
LPSHSDFLAELR (heavy)(+3)	15.53	2	465.581	427.254	13.4	3.455
LPSHSDFLAELR (heavy)(+3)	15.53	2	465.581	549.267	13.4	3.455
LPSHSDFLAELR (heavy)(+3)	15.53	2	465.581	549.284	13.4	3.455
LPSHSDFLAELR (heavy)(+3)	15.53	2	465.581	641.327	13.4	3.455
LPSHSDFLAELR (heavy)(+3)	15.53	2	465.581	784.362	13.4	3.455
FSAEFDFR(+2)	16.02	2	509.735	437.214	16.7	3.613
FSAEFDFR(+2)	16.02	2	509.735	584.283	15.6	3.613
FSAEFDFR(+2)	16.02	2	509.735	784.362	15.6	3.613
FSAEFDFR(+2)	16.02	2	509.735	871.394	16.7	3.613
FSAEFDFR (heavy)(+2)	16.02	2	514.739	447.223	16.7	3.613
FSAEFDFR (heavy)(+2)	16.02	2	514.739	594.291	15.6	3.613
FSAEFDFR (heavy)(+2)	16.02	2	514.739	794.371	15.6	3.613
FSAEFDFR (heavy)(+2)	16.02	2	514.739	881.403	16.7	3.613
SPVGVQPILNEHTFC[+57.021464]AGMSK(+3)	16.16	2	724.69	284.16	20.8	3.613
SPVGVQPILNEHTFC[+57.021464]AGMSK(+3)	16.16	2	724.69	802.882	20.8	3.613
SPVGVQPILNEHTFC[+57.021464]AGMSK(+3)	16.16	2	724.69	944.956	20.8	3.613
SPVGVQPILNEHTFC[+57.021464]AGMSK(+3)	16.16	2	724.69	994.49	20.8	3.613
SPVGVQPILNEHTFC[+57.021464]AGMSK (heavy)(+3)	16.16	2	727.362	284.16	20.8	3.613
SPVGVQPILNEHTFC[+57.021464]AGMSK (heavy)(+3)	16.16	2	727.362	806.889	20.8	3.613
SPVGVQPILNEHTFC[+57.021464]AGMSK (heavy)(+3)	16.16	2	727.362	948.963	20.8	3.613
SPVGVQPILNEHTFC[+57.021464]AGMSK (heavy)(+3)	16.16	2	727.362	998.497	20.8	3.613
SC[+57.021464]DIPVFMNAR(+2)	16.2	2	655.305	363.097	21.5	3.613

SC[+57.021464]DIPVFMNAR(+2)	16.2	2	655.305	476.181	21.5	3.613
SC[+57.021464]DIPVFMNAR(+2)	16.2	2	655.305	834.429	21.5	3.613
SC[+57.021464]DIPVFMNAR(+2)	16.2	2	655.305	1062.54	21.5	3.613
SC[+57.021464]DIPVFMNAR (heavy)(+2)	16.2	2	660.309	363.097	21.5	3.613
SC[+57.021464]DIPVFMNAR (heavy)(+2)	16.2	2	660.309	476.181	21.5	3.613
SC[+57.021464]DIPVFMNAR (heavy)(+2)	16.2	2	660.309	844.437	21.5	3.613
SC[+57.021464]DIPVFMNAR (heavy)(+2)	16.2	2	660.309	1072.548	21.5	3.613
YVMLPVADQDQC[+57.021464]IR(+2)	16.2	2	854.413	394.18	28.1	3.613
YVMLPVADQDQC[+57.021464]IR(+2)	16.2	2	854.413	601.285	28.1	3.613
YVMLPVADQDQC[+57.021464]IR(+2)	16.2	2	854.413	1005.442	28.1	3.613
YVMLPVADQDQC[+57.021464]IR(+2)	16.2	2	854.413	1201.563	28.1	3.613
YVMLPVADQDQC[+57.021464]IR (heavy)(+2)	16.2	2	859.417	394.18	28.1	3.613
YVMLPVADQDQC[+57.021464]IR (heavy)(+2)	16.2	2	859.417	606.289	28.1	3.613
YVMLPVADQDQC[+57.021464]IR (heavy)(+2)	16.2	2	859.417	1015.45	28.1	3.613
YVMLPVADQDQC[+57.021464]IR (heavy)(+2)	16.2	2	859.417	1211.571	28.1	3.613
VYLPWSR(+2)	16.21	2	460.753	273.145	18	3.613
VYLPWSR(+2)	16.21	2	460.753	545.283	14	3.613
VYLPWSR(+2)	16.21	2	460.753	658.367	14	3.613
VYLPWSR(+2)	16.21	2	460.753	821.43	15.1	3.613
VYLPWSR (heavy)(+2)	16.21	2	465.757	278.149	18	3.613
VYLPWSR (heavy)(+2)	16.21	2	465.757	555.291	14	3.613
VYLPWSR (heavy)(+2)	16.21	2	465.757	668.375	14	3.613
VYLPWSR (heavy)(+2)	16.21	2	465.757	831.439	15.1	3.613
WPEPVFGR(+2)	16.21	2	494.255	401.216	16.2	3.613
WPEPVFGR(+2)	16.21	2	494.255	575.33	16.2	3.613
WPEPVFGR(+2)	16.21	2	494.255	704.373	16.2	3.613
WPEPVFGR(+2)	16.21	2	494.255	801.425	16.2	3.613
WPEPVFGR (heavy)(+2)	16.21	2	499.26	406.22	16.2	3.613
WPEPVFGR (heavy)(+2)	16.21	2	499.26	585.338	16.2	3.613
WPEPVFGR (heavy)(+2)	16.21	2	499.26	714.381	16.2	3.613
WPEPVFGR (heavy)(+2)	16.21	2	499.26	811.434	16.2	3.613
GFSWVGGGEDTPYSNWHK(+3)	16.43	2	675.302	724.315	19.4	3.613
GFSWVGGGEDTPYSNWHK(+3)	16.43	2	675.302	777.828	19.4	3.613
GFSWVGGGEDTPYSNWHK(+3)	16.43	2	675.302	910.405	19.4	3.613
GFSWVGGGEDTPYSNWHK (heavy)(+3)	16.43	2	677.974	728.323	19.4	3.613
GFSWVGGGEDTPYSNWHK (heavy)(+3)	16.43	2	677.974	777.828	19.4	3.613
GFSWVGGGEDTPYSNWHK (heavy)(+3)	16.43	2	677.974	914.412	19.4	3.613
TVEGVKDLQAQVVESAK (heavy)(+3)	16.5	2	606.337	541.307	17.3	3.613
TVEGVKDLQAQVVESAK (heavy)(+3)	16.5	2	606.337	744.424	17.3	3.613
TVEGVKDLQAQVVESAK (heavy)(+3)	16.5	2	606.337	808.945	17.3	3.613
DPTFIPAPIQAK(+2)	16.68	2	649.361	314.135	26.4	3.613
DPTFIPAPIQAK(+2)	16.68	2	649.361	461.203	22.4	3.613
DPTFIPAPIQAK(+2)	16.68	2	649.361	574.287	18.4	3.613
DPTFIPAPIQAK(+2)	16.68	2	649.361	724.435	18.4	3.613
DPTFIPAPIQAK (heavy)(+2)	16.68	2	653.368	314.135	26.4	3.613
DPTFIPAPIQAK (heavy)(+2)	16.68	2	653.368	461.203	22.4	3.613
DPTFIPAPIQAK (heavy)(+2)	16.68	2	653.368	574.287	18.4	3.613
DPTFIPAPIQAK (heavy)(+2)	16.68	2	653.368	732.449	18.4	3.613
VLHGDLIDFVC[+57.021464]K(+3)	16.82	2	472.583	406.212	13.7	3.613
VLHGDLIDFVC[+57.021464]K(+3)	16.82	2	472.583	553.28	13.5	3.613
VLHGDLIDFVC[+57.021464]K(+3)	16.82	2	472.583	635.351	17.5	3.613
VLHGDLIDFVC[+57.021464]K(+3)	16.82	2	472.583	668.307	11.5	3.613
VLHGDLIDFVC[+57.021464]K (heavy)(+3)	16.82	2	475.254	414.226	13.7	3.613
VLHGDLIDFVC[+57.021464]K (heavy)(+3)	16.82	2	475.254	561.294	13.5	3.613
VLHGDLIDFVC[+57.021464]K (heavy)(+3)	16.82	2	475.254	635.351	17.5	3.613
VLHGDLIDFVC[+57.021464]K (heavy)(+3)	16.82	2	475.254	676.321	11.5	3.613
FYYIVQEEK(+2)	16.85	2	609.805	474.202	20	3.613
FYYIVQEEK(+2)	16.85	2	609.805	632.325	20	3.613
FYYIVQEEK(+2)	16.85	2	609.805	745.409	20	3.613
FYYIVQEEK(+2)	16.85	2	609.805	908.472	20	3.613
FYYIVQEEK (heavy)(+2)	16.85	2	613.812	474.202	20	3.613
FYYIVQEEK (heavy)(+2)	16.85	2	613.812	640.339	20	3.613
FYYIVQEEK (heavy)(+2)	16.85	2	613.812	753.423	20	3.613
FYYIVQEEK (heavy)(+2)	16.85	2	613.812	916.487	20	3.613
VFIGINDLEK(+2)	16.94	2	574.321	451.253	15.8	3.613
VFIGINDLEK(+2)	16.94	2	574.321	731.393	17.8	3.613
VFIGINDLEK(+2)	16.94	2	574.321	788.415	17.8	3.613
VFIGINDLEK(+2)	16.94	2	574.321	901.499	17.8	3.613
VFIGINDLEK (heavy)(+2)	16.94	2	578.328	455.26	15.8	3.613
VFIGINDLEK (heavy)(+2)	16.94	2	578.328	739.408	17.8	3.613
VFIGINDLEK (heavy)(+2)	16.94	2	578.328	796.429	17.8	3.613
VFIGINDLEK (heavy)(+2)	16.94	2	578.328	909.513	17.8	3.613
RPDSLQHVLLPVLDR(+3)	17.01	2	586.672	417.714	22.8	3.613
RPDSLQHVLLPVLDR(+3)	17.01	2	586.672	467.249	18.8	3.613
RPDSLQHVLLPVLDR(+3)	17.01	2	586.672	523.791	16.9	3.613
RPDSLQHVLLPVLDR(+3)	17.01	2	586.672	599.351	20.8	3.613
RPDSLQHVLLPVLDR(+3)	17.01	2	586.672	825.519	20.8	3.613

RPDSLQHVLLPVLDR (heavy)(+3)	17.01	2	593.344	422.719	22.8	3.613
RPDSLQHVLLPVLDR (heavy)(+3)	17.01	2	593.344	472.253	18.8	3.613
RPDSLQHVLLPVLDR (heavy)(+3)	17.01	2	593.344	528.795	16.9	3.613
RPDSLQHVLLPVLDR (heavy)(+3)	17.01	2	593.344	609.359	20.8	3.613
RPDSLQHVLLPVLDR (heavy)(+3)	17.01	2	593.344	835.528	20.8	3.613
NDFTWFK(+2)	17.03	2	479.226	377.146	15.7	3.613
NDFTWFK(+2)	17.03	2	479.226	581.308	15.7	3.613
NDFTWFK(+2)	17.03	2	479.226	728.377	15.7	3.613
NDFTWFK(+2)	17.03	2	479.226	843.404	15.7	3.613
NDFTWFK (heavy)(+2)	17.03	2	483.233	377.146	15.7	3.613
NDFTWFK (heavy)(+2)	17.03	2	483.233	589.322	15.7	3.613
NDFTWFK (heavy)(+2)	17.03	2	483.233	736.391	15.7	3.613
NDFTWFK (heavy)(+2)	17.03	2	483.233	851.418	15.7	3.613
FWIGLQR(+2)	17.13	2	460.261	386.727	15.1	3.767
FWIGLQR(+2)	17.13	2	460.261	473.283	15.1	3.767
FWIGLQR(+2)	17.13	2	460.261	586.367	15.1	3.767
FWIGLQR(+2)	17.13	2	460.261	772.446	15.1	3.767
FWIGLQR (heavy)(+2)	17.13	2	465.265	391.731	15.1	3.767
FWIGLQR (heavy)(+2)	17.13	2	465.265	483.291	15.1	3.767
FWIGLQR (heavy)(+2)	17.13	2	465.265	596.375	15.1	3.767
FWIGLQR (heavy)(+2)	17.13	2	465.265	782.455	15.1	3.767
LAVTTHGLPC[+57.021464]LAWASAQAK(+3)	17.55	2	665.687	575.315	19.1	4.178
LAVTTHGLPC[+57.021464]LAWASAQAK(+3)	17.55	2	665.687	761.394	19.1	4.178
LAVTTHGLPC[+57.021464]LAWASAQAK(+3)	17.55	2	665.687	793.457	19.1	4.178
LAVTTHGLPC[+57.021464]LAWASAQAK(+3)	17.55	2	665.687	832.431	19.1	4.178
LAVTTHGLPC[+57.021464]LAWASAQAK (heavy)(+3)	17.55	2	668.358	583.329	19.1	4.178
LAVTTHGLPC[+57.021464]LAWASAQAK (heavy)(+3)	17.55	2	668.358	769.408	19.1	4.178
LAVTTHGLPC[+57.021464]LAWASAQAK (heavy)(+3)	17.55	2	668.358	793.457	19.1	4.178
LAVTTHGLPC[+57.021464]LAWASAQAK (heavy)(+3)	17.55	2	668.358	840.445	19.1	4.178
RPWNVASLIYETK(+3)	17.61	2	526.287	540.266	15.2	4.178
RPWNVASLIYETK(+3)	17.61	2	526.287	653.35	15.2	4.178
RPWNVASLIYETK(+3)	17.61	2	526.287	653.352	15.2	4.178
RPWNVASLIYETK(+3)	17.61	2	526.287	853.467	15.2	4.178
RPWNVASLIYETK (heavy)(+3)	17.61	2	532.295	548.281	15.2	4.178
RPWNVASLIYETK (heavy)(+3)	17.61	2	532.295	661.365	15.2	4.178
RPWNVASLIYETK (heavy)(+3)	17.61	2	532.295	663.36	15.2	4.178
RPWNVASLIYETK (heavy)(+3)	17.61	2	532.295	861.481	15.2	4.178
ALLVGEHLNIIVTPK(+3)	17.67	2	539.662	298.213	15.6	4.178
ALLVGEHLNIIVTPK(+3)	17.67	2	539.662	610.854	15.6	4.178
ALLVGEHLNIIVTPK(+3)	17.67	2	539.662	660.388	15.6	4.178
ALLVGEHLNIIVTPK(+3)	17.67	2	539.662	716.93	15.6	4.178
ALLVGEHLNIIVTPK (heavy)(+3)	17.67	2	542.334	298.213	15.6	4.178
ALLVGEHLNIIVTPK (heavy)(+3)	17.67	2	542.334	614.861	15.6	4.178
ALLVGEHLNIIVTPK (heavy)(+3)	17.67	2	542.334	664.395	15.6	4.178
ALLVGEHLNIIVTPK (heavy)(+3)	17.67	2	542.334	720.937	15.6	4.178
NTC[+57.021464]NHDEDTWVEC[+57.021464]EDPFDLR(+3)	17.67	2	851.341	647.351	30.6	4.178
NTC[+57.021464]NHDEDTWVEC[+57.021464]EDPFDLR(+3)	17.67	2	851.341	762.378	24.4	4.178
NTC[+57.021464]NHDEDTWVEC[+57.021464]EDPFDLR(+3)	17.67	2	851.341	1051.451	26.6	4.178
NTC[+57.021464]NHDEDTWVEC[+57.021464]EDPFDLR(+3)	17.67	2	851.341	1180.494	24.6	4.178
NTC[+57.021464]NHDEDTWVEC[+57.021464]EDPFDLR (heavy)(+3)	17.67	2	854.677	657.359	30.6	4.178
NTC[+57.021464]NHDEDTWVEC[+57.021464]EDPFDLR (heavy)(+3)	17.67	2	854.677	772.386	24.4	4.178
NTC[+57.021464]NHDEDTWVEC[+57.021464]EDPFDLR (heavy)(+3)	17.67	2	854.677	1061.46	26.6	4.178
NTC[+57.021464]NHDEDTWVEC[+57.021464]EDPFDLR (heavy)(+3)	17.67	2	854.677	1190.502	24.6	4.178
GLAFTDVDVDSIK(+2)	17.72	2	690.356	242.15	22.7	4.178
GLAFTDVDVDSIK(+2)	17.72	2	690.356	890.447	22.7	4.178
GLAFTDVDVDSIK(+2)	17.72	2	690.356	991.494	22.7	4.178
GLAFTDVDVDSIK(+2)	17.72	2	690.356	1138.563	22.7	4.178
GLAFTDVDVDSIK (heavy)(+2)	17.72	2	694.363	242.15	22.7	4.178
GLAFTDVDVDSIK (heavy)(+2)	17.72	2	694.363	898.461	22.7	4.178
GLAFTDVDVDSIK (heavy)(+2)	17.72	2	694.363	999.508	22.7	4.178
GLAFTDVDVDSIK (heavy)(+2)	17.72	2	694.363	1146.577	22.7	4.178
AEATTLHVAPQGTAMAVSTFR(+3)	17.74	2	720.368	510.267	20.7	4.178
AEATTLHVAPQGTAMAVSTFR(+3)	17.74	2	720.368	609.335	20.7	4.178
AEATTLHVAPQGTAMAVSTFR(+3)	17.74	2	720.368	680.373	20.7	4.178
AEATTLHVAPQGTAMAVSTFR(+3)	17.74	2	720.368	894.468	20.7	4.178
AEATTLHVAPQGTAMAVSTFR (heavy)(+3)	17.74	2	723.704	520.275	20.7	4.178
AEATTLHVAPQGTAMAVSTFR (heavy)(+3)	17.74	2	723.704	619.344	20.7	4.178
AEATTLHVAPQGTAMAVSTFR (heavy)(+3)	17.74	2	723.704	690.381	20.7	4.178
AEATTLHVAPQGTAMAVSTFR (heavy)(+3)	17.74	2	723.704	894.468	20.7	4.178
TNLESILSYPK(+2)	17.83	2	632.842	329.182	18.8	4.178
TNLESILSYPK(+2)	17.83	2	632.842	607.345	18.8	4.178
TNLESILSYPK(+2)	17.83	2	632.842	807.461	20.8	4.178
TNLESILSYPK(+2)	17.83	2	632.842	1049.588	18.8	4.178
TNLESILSYPK (heavy)(+2)	17.83	2	636.849	329.182	18.8	4.178

TNLESILSYPK (heavy)(+2)	17.83	2	636.849	615.359	18.8	4.178
TNLESILSYPK (heavy)(+2)	17.83	2	636.849	815.475	20.8	4.178
TNLESILSYPK (heavy)(+2)	17.83	2	636.849	1057.602	18.8	4.178
GVTSVSQIFHSPDLAIR(+3)	17.93	2	609.663	692.373	13.5	4.178
GVTSVSQIFHSPDLAIR(+3)	17.93	2	609.663	741.907	17.5	4.178
GVTSVSQIFHSPDLAIR(+3)	17.93	2	609.663	785.423	17.5	4.178
GVTSVSQIFHSPDLAIR(+3)	17.93	2	609.663	835.947	15.5	4.178
GVTSVSQIFHSPDLAIR (heavy)(+3)	17.93	2	612.999	697.377	13.5	4.178
GVTSVSQIFHSPDLAIR (heavy)(+3)	17.93	2	612.999	746.911	17.5	4.178
GVTSVSQIFHSPDLAIR (heavy)(+3)	17.93	2	612.999	790.427	17.5	4.178
GVTSVSQIFHSPDLAIR (heavy)(+3)	17.93	2	612.999	840.951	15.5	4.178
ISEATDGLSDFLK (heavy)(+2)	17.94	2	702.36	902.471	23	4.178
ISEATDGLSDFLK (heavy)(+2)	17.94	2	702.36	1003.519	22	4.178
ISEATDGLSDFLK (heavy)(+2)	17.94	2	702.36	1074.556	22	4.178
FIINDWVK(+2)	18.3	2	517.787	432.261	17	5.728
FIINDWVK(+2)	18.3	2	517.787	661.33	17	5.728
FIINDWVK(+2)	18.3	2	517.787	774.414	17	5.728
FIINDWVK(+2)	18.3	2	517.787	887.499	17	5.728
FIINDWVK (heavy)(+2)	18.3	2	521.794	440.275	17	5.728
FIINDWVK (heavy)(+2)	18.3	2	521.794	669.345	17	5.728
FIINDWVK (heavy)(+2)	18.3	2	521.794	782.429	17	5.728
FIINDWVK (heavy)(+2)	18.3	2	521.794	895.513	17	5.728
LSLEIEQLELQR(+2)	18.79	2	735.911	443.25	24.2	5.866
LSLEIEQLELQR(+2)	18.79	2	735.911	545.304	24.2	5.866
LSLEIEQLELQR(+2)	18.79	2	735.911	786.447	24.2	5.866
LSLEIEQLELQR(+2)	18.79	2	735.911	915.489	24.2	5.866
LSLEIEQLELQR(+2)	18.79	2	735.911	1028.573	24.2	5.866
LSLEIEQLELQR (heavy)(+2)	18.79	2	740.915	443.25	24.2	5.866
LSLEIEQLELQR (heavy)(+2)	18.79	2	740.915	555.312	24.2	5.866
LSLEIEQLELQR (heavy)(+2)	18.79	2	740.915	796.455	24.2	5.866
LSLEIEQLELQR (heavy)(+2)	18.79	2	740.915	925.498	24.2	5.866
LSLEIEQLELQR (heavy)(+2)	18.79	2	740.915	1038.582	24.2	5.866
HQDFNSAVQLVENFC[+57.021464]R(+3)	18.87	2	655.307	725.304	18.8	5.866
HQDFNSAVQLVENFC[+57.021464]R(+3)	18.87	2	655.307	800.332	18.8	5.866
HQDFNSAVQLVENFC[+57.021464]R(+3)	18.87	2	655.307	824.372	18.8	5.866
HQDFNSAVQLVENFC[+57.021464]R(+3)	18.87	2	655.307	937.456	18.8	5.866
HQDFNSAVQLVENFC[+57.021464]R (heavy)(+3)	18.87	2	658.643	735.312	18.8	5.866
HQDFNSAVQLVENFC[+57.021464]R (heavy)(+3)	18.87	2	658.643	800.332	18.8	5.866
HQDFNSAVQLVENFC[+57.021464]R (heavy)(+3)	18.87	2	658.643	834.38	18.8	5.866
HQDFNSAVQLVENFC[+57.021464]R (heavy)(+3)	18.87	2	658.643	947.464	18.8	5.866
WLTFSLGK(+2)	18.98	2	476.268	404.25	15.6	5.866
WLTFSLGK(+2)	18.98	2	476.268	551.319	15.6	5.866
WLTFSLGK(+2)	18.98	2	476.268	652.366	15.6	5.866
WLTFSLGK(+2)	18.98	2	476.268	765.451	15.6	5.866
WLTFSLGK (heavy)(+2)	18.98	2	480.275	412.265	15.6	5.866
WLTFSLGK (heavy)(+2)	18.98	2	480.275	559.333	15.6	5.866
WLTFSLGK (heavy)(+2)	18.98	2	480.275	660.381	15.6	5.866
WLTFSLGK (heavy)(+2)	18.98	2	480.275	773.465	15.6	5.866
VDLVDFEDNYQFAK(+2)	19.1	2	851.901	328.187	28	5.866
VDLVDFEDNYQFAK(+2)	19.1	2	851.901	1161.521	28	5.866
VDLVDFEDNYQFAK(+2)	19.1	2	851.901	1276.548	28	5.866
VDLVDFEDNYQFAK (heavy)(+2)	19.1	2	855.908	328.187	28	5.866
VDLVDFEDNYQFAK (heavy)(+2)	19.1	2	855.908	1169.535	28	5.866
VDLVDFEDNYQFAK (heavy)(+2)	19.1	2	855.908	1284.562	28	5.866
TLVVNPWLTQVR(+2)	19.18	2	713.414	314.207	23.5	5.866
TLVVNPWLTQVR(+2)	19.18	2	713.414	899.51	23.5	5.866
TLVVNPWLTQVR(+2)	19.18	2	713.414	1013.553	23.5	5.866
TLVVNPWLTQVR(+2)	19.18	2	713.414	1112.621	23.5	5.866
TLVVNPWLTQVR (heavy)(+2)	19.18	2	718.418	314.207	23.5	5.866
TLVVNPWLTQVR (heavy)(+2)	19.18	2	718.418	909.518	23.5	5.866
TLVVNPWLTQVR (heavy)(+2)	19.18	2	718.418	1023.561	23.5	5.866
TLVVNPWLTQVR (heavy)(+2)	19.18	2	718.418	1122.629	23.5	5.866
SLQTFSQAWFTC[+57.021464]R(+2)	19.3	2	816.385	583.266	26.9	5.866
SLQTFSQAWFTC[+57.021464]R(+2)	19.3	2	816.385	769.345	26.9	5.866
SLQTFSQAWFTC[+57.021464]R(+2)	19.3	2	816.385	1055.473	26.9	5.866
SLQTFSQAWFTC[+57.021464]R(+2)	19.3	2	816.385	1303.589	26.9	5.866
SLQTFSQAWFTC[+57.021464]R (heavy)(+2)	19.3	2	821.389	593.274	26.9	5.866
SLQTFSQAWFTC[+57.021464]R (heavy)(+2)	19.3	2	821.389	779.353	26.9	5.866
SLQTFSQAWFTC[+57.021464]R (heavy)(+2)	19.3	2	821.389	1065.481	26.9	5.866
SLQTFSQAWFTC[+57.021464]R (heavy)(+2)	19.3	2	821.389	1313.597	26.9	5.866
SGQQAC[+57.021464]EGVGSFLLYSVHEGIR(+3)	19.34	2	798.721	739.394	22.9	5.866
SGQQAC[+57.021464]EGVGSFLLYSVHEGIR(+3)	19.34	2	798.721	817.438	22.9	5.866
SGQQAC[+57.021464]EGVGSFLLYSVHEGIR(+3)	19.34	2	798.721	961.975	22.9	5.866
SGQQAC[+57.021464]EGVGSFLLYSVHEGIR(+3)	19.34	2	798.721	997.494	22.9	5.866
SGQQAC[+57.021464]EGVGSFLLYSVHEGIR (heavy)(+3)	19.34	2	802.057	744.398	22.9	5.866
SGQQAC[+57.021464]EGVGSFLLYSVHEGIR (heavy)(+3)	19.34	2	802.057	822.443	22.9	5.866
SGQQAC[+57.021464]EGVGSFLLYSVHEGIR (heavy)(+3)	19.34	2	802.057	966.979	22.9	5.866

SGQQAC[+57.021464]EGVGSFLLYSVHEGIR (heavy)(+3)	19.34	2	802.057	1002.498	22.9	5.866
C[+57.021464]GILGHC[+57.021464]QAPDHFLFAK(+3)	19.39	2	657.65	331.143	18.9	5.866
C[+57.021464]GILGHC[+57.021464]QAPDHFLFAK(+3)	19.39	2	657.65	764.362	18.9	5.866
C[+57.021464]GILGHC[+57.021464]QAPDHFLFAK(+3)	19.39	2	657.65	820.904	18.9	5.866
C[+57.021464]GILGHC[+57.021464]QAPDHFLFAK(+3)	19.39	2	657.65	974.509	18.9	5.866
C[+57.021464]GILGHC[+57.021464]QAPDHFLFAK (heavy)(+3)	19.39	2	660.321	331.143	18.9	5.866
C[+57.021464]GILGHC[+57.021464]QAPDHFLFAK (heavy)(+3)	19.39	2	660.321	768.369	18.9	5.866
C[+57.021464]GILGHC[+57.021464]QAPDHFLFAK (heavy)(+3)	19.39	2	660.321	824.911	18.9	5.866
C[+57.021464]GILGHC[+57.021464]QAPDHFLFAK (heavy)(+3)	19.39	2	660.321	982.524	18.9	5.866
LGEFWLGNDNIHALTAQGTSELR(+3)	20.08	2	848.094	898.948	24.3	10.284
LGEFWLGNDNIHALTAQGTSELR(+3)	20.08	2	848.094	955.49	24.3	10.284
LGEFWLGNDNIHALTAQGTSELR(+3)	20.08	2	848.094	1048.53	24.3	10.284
LGEFWLGNDNIHALTAQGTSELR (heavy)(+3)	20.08	2	851.43	903.952	24.3	10.284
LGEFWLGNDNIHALTAQGTSELR (heavy)(+3)	20.08	2	851.43	960.494	24.3	10.284
LGEFWLGNDNIHALTAQGTSELR (heavy)(+3)	20.08	2	851.43	1053.534	24.3	10.284
FTQVTPTSLSAQWTPPNVQLTGYR(+3)	20.29	2	898.129	572.809	25.7	10.284
FTQVTPTSLSAQWTPPNVQLTGYR(+3)	20.29	2	898.129	577.298	25.7	10.284
FTQVTPTSLSAQWTPPNVQLTGYR(+3)	20.29	2	898.129	1144.611	25.7	10.284
FTQVTPTSLSAQWTPPNVQLTGYR(+3)	20.29	2	898.129	1245.659	25.7	10.284
FTQVTPTSLSAQWTPPNVQLTGYR (heavy)(+3)	20.29	2	901.465	577.298	25.7	10.284
FTQVTPTSLSAQWTPPNVQLTGYR (heavy)(+3)	20.29	2	901.465	577.813	25.7	10.284
FTQVTPTSLSAQWTPPNVQLTGYR (heavy)(+3)	20.29	2	901.465	1154.619	25.7	10.284
FTQVTPTSLSAQWTPPNVQLTGYR (heavy)(+3)	20.29	2	901.465	1255.667	25.7	10.284
ILLQGTPVAQMTEDAVDAER(+3)	20.3	2	719.7	490.226	15.7	10.555
ILLQGTPVAQMTEDAVDAER(+3)	20.3	2	719.7	904.401	19.7	10.555
ILLQGTPVAQMTEDAVDAER(+3)	20.3	2	719.7	1005.448	20.7	10.555
ILLQGTPVAQMTEDAVDAER (heavy)(+3)	20.3	2	723.036	500.234	15.7	10.555
ILLQGTPVAQMTEDAVDAER (heavy)(+3)	20.3	2	723.036	914.409	19.7	10.555
ILLQGTPVAQMTEDAVDAER (heavy)(+3)	20.3	2	723.036	1015.457	20.7	10.555
VEEQEPELTSTPNFVVEVIK(+3)	20.83	2	763.061	522.808	21.9	10.559
VEEQEPELTSTPNFVVEVIK(+3)	20.83	2	763.061	587.376	21.9	10.559
VEEQEPELTSTPNFVVEVIK(+3)	20.83	2	763.061	841.357	21.9	10.559
VEEQEPELTSTPNFVVEVIK(+3)	20.83	2	763.061	1044.609	21.9	10.559
VEEQEPELTSTPNFVVEVIK (heavy)(+3)	20.83	2	765.733	526.815	21.9	10.559
VEEQEPELTSTPNFVVEVIK (heavy)(+3)	20.83	2	765.733	595.39	21.9	10.559
VEEQEPELTSTPNFVVEVIK (heavy)(+3)	20.83	2	765.733	841.357	21.9	10.559
VEEQEPELTSTPNFVVEVIK (heavy)(+3)	20.83	2	765.733	1052.623	21.9	10.559
SAGFDFSFLPQPPQEK(+2)	20.97	2	897.938	598.32	29.6	10.559
SAGFDFSFLPQPPQEK(+2)	20.97	2	897.938	823.431	29.6	10.559
SAGFDFSFLPQPPQEK(+2)	20.97	2	897.938	936.515	29.6	10.559
SAGFDFSFLPQPPQEK(+2)	20.97	2	897.938	1083.583	29.6	10.559
SAGFDFSFLPQPPQEK(+2)	20.97	2	897.938	1170.615	29.6	10.559
SAGFDFSFLPQPPQEK (heavy)(+2)	20.97	2	901.945	606.334	29.6	10.559
SAGFDFSFLPQPPQEK (heavy)(+2)	20.97	2	901.945	831.445	29.6	10.559
SAGFDFSFLPQPPQEK (heavy)(+2)	20.97	2	901.945	944.529	29.6	10.559
SAGFDFSFLPQPPQEK (heavy)(+2)	20.97	2	901.945	1091.598	29.6	10.559
SAGFDFSFLPQPPQEK (heavy)(+2)	20.97	2	901.945	1178.63	29.6	10.559
ASSIIDELFQDR(+2)	21.33	2	697.351	359.193	22.9	11.5
ASSIIDELFQDR(+2)	21.33	2	697.351	565.273	19.9	11.5
ASSIIDELFQDR(+2)	21.33	2	697.351	922.426	22.9	11.5
ASSIIDELFQDR(+2)	21.33	2	697.351	1035.511	22.9	11.5
ASSIIDELFQDR (heavy)(+2)	21.33	2	702.355	359.193	22.9	11.5
ASSIIDELFQDR (heavy)(+2)	21.33	2	702.355	575.281	19.9	11.5
ASSIIDELFQDR (heavy)(+2)	21.33	2	702.355	932.435	22.9	11.5
ASSIIDELFQDR (heavy)(+2)	21.33	2	702.355	1045.519	22.9	11.5
LLIYAVLPTGDVIGDSAK(+2)	21.64	2	923.022	673.428	30.4	11.5
LLIYAVLPTGDVIGDSAK(+2)	21.64	2	923.022	1059.532	30.4	11.5
LLIYAVLPTGDVIGDSAK(+2)	21.64	2	923.022	1172.616	30.4	11.5
LLIYAVLPTGDVIGDSAK(+2)	21.64	2	923.022	1271.684	30.4	11.5
LLIYAVLPTGDVIGDSAK (heavy)(+2)	21.64	2	927.029	673.428	30.4	11.5
LLIYAVLPTGDVIGDSAK (heavy)(+2)	21.64	2	927.029	1067.546	30.4	11.5
LLIYAVLPTGDVIGDSAK (heavy)(+2)	21.64	2	927.029	1180.63	30.4	11.5
LLIYAVLPTGDVIGDSAK (heavy)(+2)	21.64	2	927.029	1279.698	30.4	11.5
GSSTWLTAFVLK(+2)	21.66	2	655.361	577.371	21.5	11.5
GSSTWLTAFVLK(+2)	21.66	2	655.361	678.418	21.5	11.5
GSSTWLTAFVLK(+2)	21.66	2	655.361	791.503	21.5	11.5
GSSTWLTAFVLK(+2)	21.66	2	655.361	977.582	21.5	11.5
GSSTWLTAFVLK (heavy)(+2)	21.66	2	659.368	585.385	21.5	11.5
GSSTWLTAFVLK (heavy)(+2)	21.66	2	659.368	686.433	21.5	11.5
GSSTWLTAFVLK (heavy)(+2)	21.66	2	659.368	799.517	21.5	11.5
GSSTWLTAFVLK (heavy)(+2)	21.66	2	659.368	985.596	21.5	11.5
IEFPILEDSSELQLK(+2)	21.88	2	880.969	686.372	29	11.5
IEFPILEDSSELQLK(+2)	21.88	2	880.969	759.906	29	11.5
IEFPILEDSSELQLK(+2)	21.88	2	880.969	1161.6	29	11.5
IEFPILEDSSELQLK(+2)	21.88	2	880.969	1371.737	29	11.5
IEFPILEDSSELQLK (heavy)(+2)	21.88	2	884.976	690.379	29	11.5
IEFPILEDSSELQLK (heavy)(+2)	21.88	2	884.976	763.913	29	11.5

IEFPILEDSSELQLK (heavy)(+2)	21.88	2	884.976	1169.614	29	11.5
IEFPILEDSSELQLK (heavy)(+2)	21.88	2	884.976	1379.751	29	11.5
DVWGIEGPIDAAFTR(+2)	21.98	2	823.912	458.203	26.3	11.5
DVWGIEGPIDAAFTR(+2)	21.98	2	823.912	716.865	24.3	11.5
DVWGIEGPIDAAFTR(+2)	21.98	2	823.912	947.495	24.3	11.5
DVWGIEGPIDAAFTR(+2)	21.98	2	823.912	1076.537	24.3	11.5
DVWGIEGPIDAAFTR (heavy)(+2)	21.98	2	828.916	458.203	26.3	11.5
DVWGIEGPIDAAFTR (heavy)(+2)	21.98	2	828.916	721.869	24.3	11.5
DVWGIEGPIDAAFTR (heavy)(+2)	21.98	2	828.916	957.503	24.3	11.5
DVWGIEGPIDAAFTR (heavy)(+2)	21.98	2	828.916	1086.545	24.3	11.5
DSSTWLTAFVLK(+2)	23.56	2	684.363	678.418	22.5	20.403
DSSTWLTAFVLK(+2)	23.56	2	684.363	977.582	22.5	20.403
DSSTWLTAFVLK(+2)	23.56	2	684.363	1078.63	22.5	20.403
DSSTWLTAFVLK (heavy)(+2)	23.56	2	688.371	686.433	22.5	20.403
DSSTWLTAFVLK (heavy)(+2)	23.56	2	688.371	985.596	22.5	20.403
DSSTWLTAFVLK (heavy)(+2)	23.56	2	688.371	1086.644	22.5	20.403
DTMVEDLVVLVR(+2)	23.59	2	694.876	348.122	22.8	20.403
DTMVEDLVVLVR(+2)	23.59	2	694.876	813.519	22.8	20.403
DTMVEDLVVLVR(+2)	23.59	2	694.876	942.562	22.8	20.403
DTMVEDLVVLVR(+2)	23.59	2	694.876	1041.63	22.8	20.403
DTMVEDLVVLVR (heavy)(+2)	23.59	2	699.88	348.122	22.8	20.403
DTMVEDLVVLVR (heavy)(+2)	23.59	2	699.88	823.528	22.8	20.403
DTMVEDLVVLVR (heavy)(+2)	23.59	2	699.88	952.57	22.8	20.403
DTMVEDLVVLVR (heavy)(+2)	23.59	2	699.88	1051.639	22.8	20.403
VAQVIIPSTYVPGTTNHDIALLR(+3)	25.13	2	826.795	299.171	27.9	31.992
VAQVIIPSTYVPGTTNHDIALLR(+3)	25.13	2	826.795	398.24	23.7	31.992
VAQVIIPSTYVPGTTNHDIALLR(+3)	25.13	2	826.795	927.989	19.9	31.992
VAQVIIPSTYVPGTTNHDIALLR(+3)	25.13	2	826.795	984.531	19.9	31.992
VAQVIIPSTYVPGTTNHDIALLR (heavy)(+3)	25.13	2	830.131	299.171	27.9	31.992
VAQVIIPSTYVPGTTNHDIALLR (heavy)(+3)	25.13	2	830.131	398.24	23.7	31.992
VAQVIIPSTYVPGTTNHDIALLR (heavy)(+3)	25.13	2	830.131	932.993	19.9	31.992
VAQVIIPSTYVPGTTNHDIALLR (heavy)(+3)	25.13	2	830.131	989.535	19.9	31.992
TFPGFFSPMLGEFVSETESR(+3)	25.54	2	755.689	621.284	21.7	31.992
TFPGFFSPMLGEFVSETESR(+3)	25.54	2	755.689	708.316	21.7	31.992
TFPGFFSPMLGEFVSETESR(+3)	25.54	2	755.689	807.384	21.7	31.992
TFPGFFSPMLGEFVSETESR(+3)	25.54	2	755.689	1008.972	21.7	31.992
TFPGFFSPMLGEFVSETESR (heavy)(+3)	25.54	2	759.025	631.292	21.7	31.992
TFPGFFSPMLGEFVSETESR (heavy)(+3)	25.54	2	759.025	718.324	21.7	31.992
TFPGFFSPMLGEFVSETESR (heavy)(+3)	25.54	2	759.025	817.393	21.7	31.992
TFPGFFSPMLGEFVSETESR (heavy)(+3)	25.54	2	759.025	1013.976	21.7	31.992
TRPVLWVGVSMQFIPAEIPR(+3)	25.86	2	766.092	341.698	22	37.184
TRPVLWVGVSMQFIPAEIPR(+3)	25.86	2	766.092	682.388	22	37.184
TRPVLWVGVSMQFIPAEIPR(+3)	25.86	2	766.092	751.403	22	37.184
TRPVLWVGVSMQFIPAEIPR(+3)	25.86	2	766.092	942.541	22	37.184
TRPVLWVGVSMQFIPAEIPR (heavy)(+3)	25.86	2	772.764	346.702	22	37.184
TRPVLWVGVSMQFIPAEIPR (heavy)(+3)	25.86	2	772.764	692.397	22	37.184
TRPVLWVGVSMQFIPAEIPR (heavy)(+3)	25.86	2	772.764	756.407	22	37.184
TRPVLWVGVSMQFIPAEIPR (heavy)(+3)	25.86	2	772.764	952.549	22	37.184
GYLFWTEWGQYPR(+2)	27.22	2	851.904	334.166	28	37.184
GYLFWTEWGQYPR(+2)	27.22	2	851.904	334.176	28	37.184
GYLFWTEWGQYPR(+2)	27.22	2	851.904	806.394	28	37.184
GYLFWTEWGQYPR(+2)	27.22	2	851.904	1036.485	28	37.184
GYLFWTEWGQYPR(+2)	27.22	2	851.904	1222.564	28	37.184
GYLFWTEWGQYPR (heavy)(+2)	27.22	2	856.908	334.166	28	37.184
GYLFWTEWGQYPR (heavy)(+2)	27.22	2	856.908	334.176	28	37.184
GYLFWTEWGQYPR (heavy)(+2)	27.22	2	856.908	816.403	28	37.184
GYLFWTEWGQYPR (heavy)(+2)	27.22	2	856.908	1046.493	28	37.184
GYLFWTEWGQYPR (heavy)(+2)	27.22	2	856.908	1232.572	28	37.184

Table S3

LC-gradient used for peptide screening

Time	Flow [ml/min]	%B	Curve
0	0.3	1	5
0.5	0.3	1	5
10	0.3	30	5
10.1	0.3	95	5
10.5	0.3	95	5
11	0.3	1	5
11.5	0.3	95	5
12	0.3	95	5
12.5	0.3	1	5
13	0.3	95	5
13.5	0.3	95	5
13.6	0.3	1	5
15	Stop Run		

LC-gradient used for elution evaluation

Time	Flow [ml/min]	%B	Curve
0	0.3	1	5
0.5	0.3	1	5
30	0.3	30	5
30.1	0.3	95	5
30.5	0.3	95	5
31	0.3	1	5
31.5	0.3	95	5
32	0.3	95	5
32.5	0.3	1	5
33	0.3	95	5
33.5	0.3	95	5
33.6	0.3	1	5
35	Stop Run		

LC-gradient used for quantitative benchmarking and long-term stability

Time	Duration	Flow [ul/min]	%B	Volume	No. Col.Vol.
0	0	6	2	0	0
30	30	6	30	180	25.34
31	1	6	80	6	0.84
31	Column Wash				
31.1	0.1	10	95	0.8	0.11
32.1	1	10	95	10	1.41
32.2	0.1	10	5	1	0.14
32.3	0.1	10	5	1	0.14
32.4	0.1	10	95	1	0.14
33.4	1	10	95	10	1.41
33.4	Stop Run				
33.4	Column Equilibration				

Figure S1

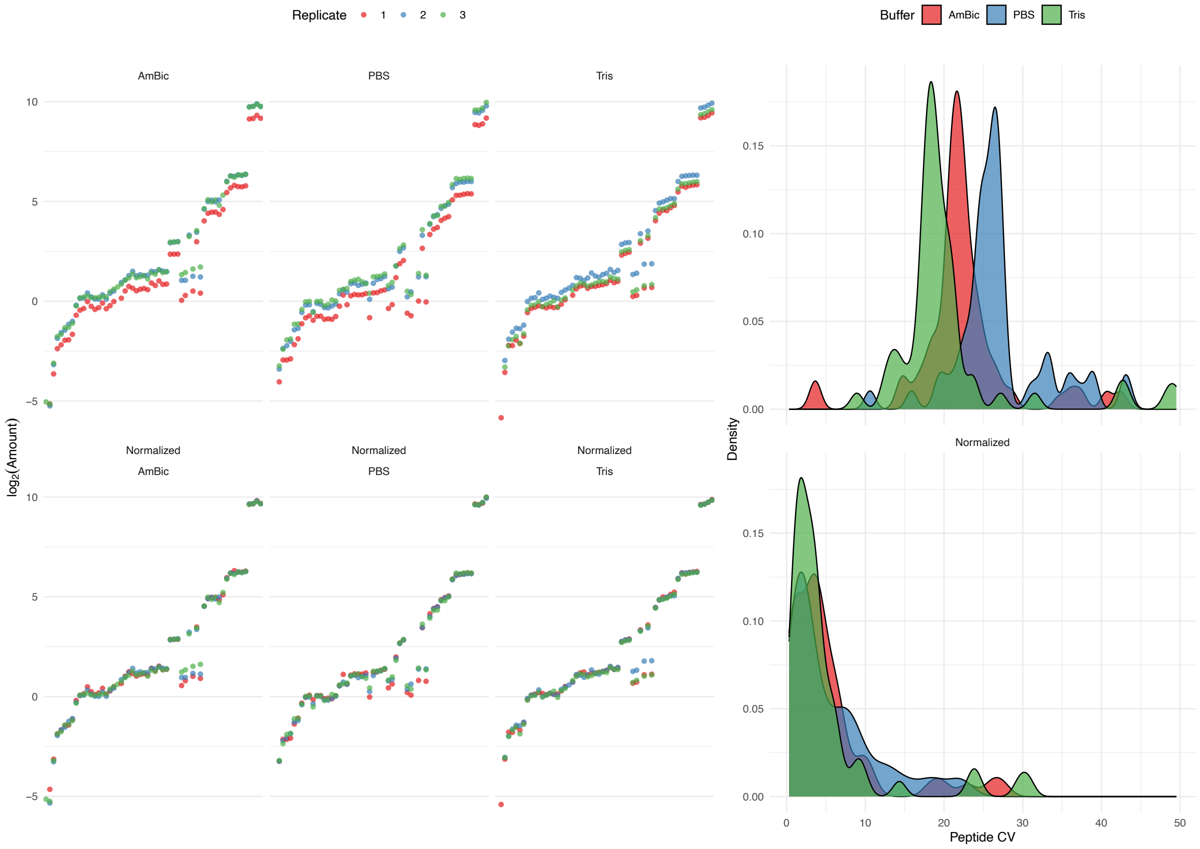

The figures compare normalized and non-normalized data obtained from samples prepared with the microsampling device. The data visualized in the upper panes is not normalized, whereas the data visualized in the lower panes is the corresponding data after normalization.

Figure S2

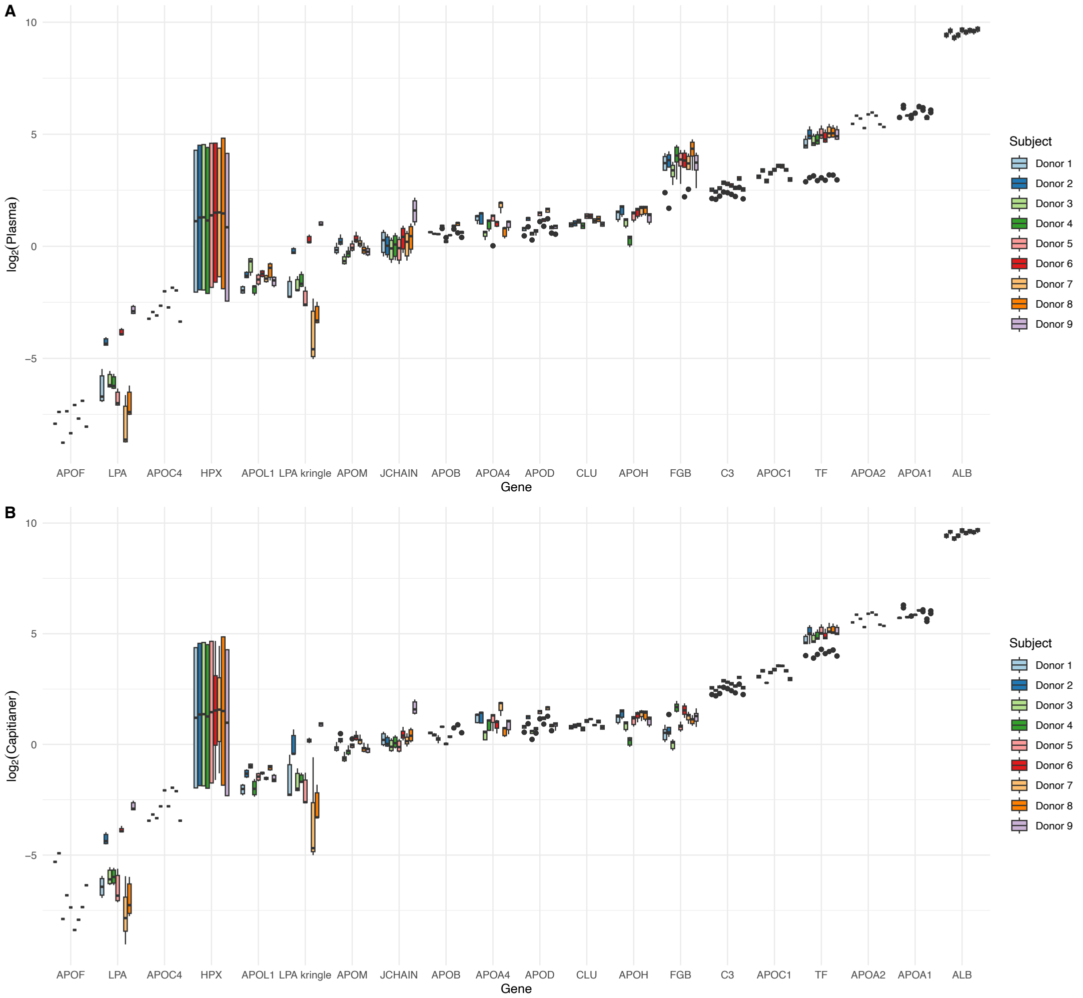

A. *A boxplot illustrating the precision of the quantification in neat plasma for nine healthy donors.* ***B.*** *A boxplot illustrating the precision of the quantification in the microsampling device for the same nine healthy donors.*

Figure S3

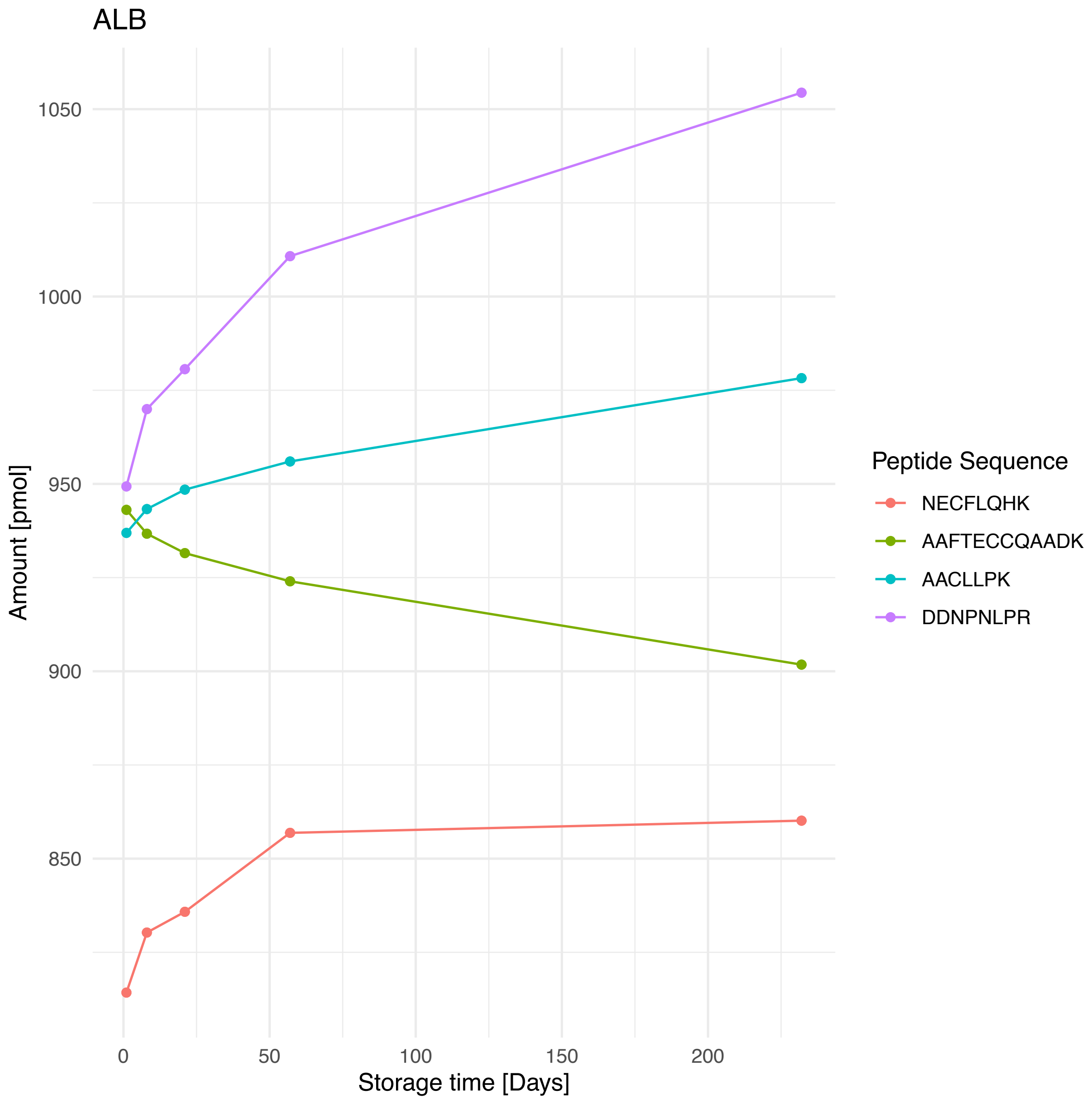
ALB
1050
1000
950
900
850
Amount [pmol]
0
50
100
150
200
Storage time [Days]
Peptide Sequence
NECFLQHK
AAFTECCQAADK
AACLLPK
DDNPNLPR

Figure S4

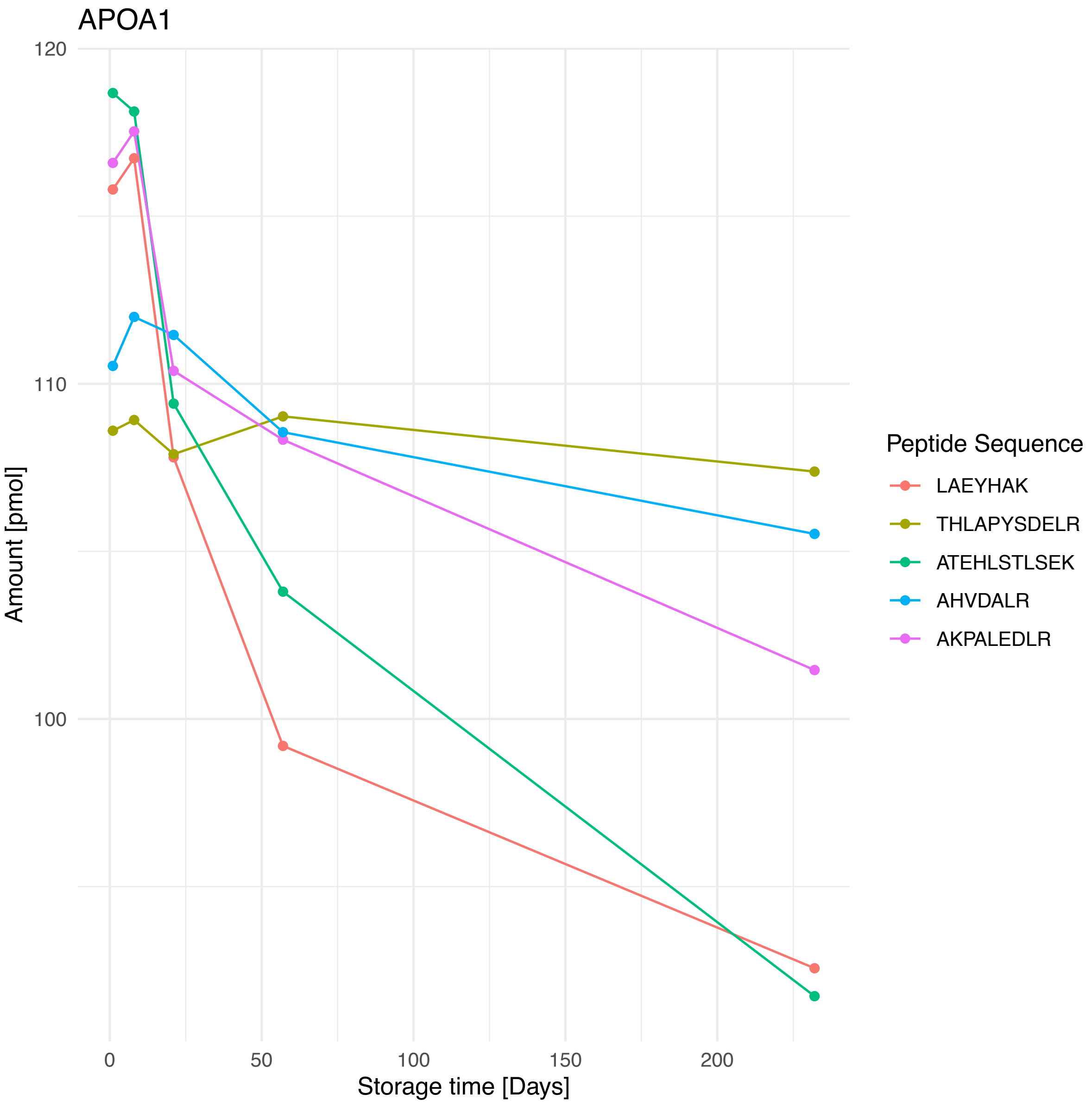
APOA1
120
110
100
Amount [pmol]
0
50
100
150
200
Storage time [Days]
Peptide Sequence
LAEYHAK
THLAPYSDELR
ATEHLSTLSEK
AHVDALR
AKPALEDLR

Figure S5

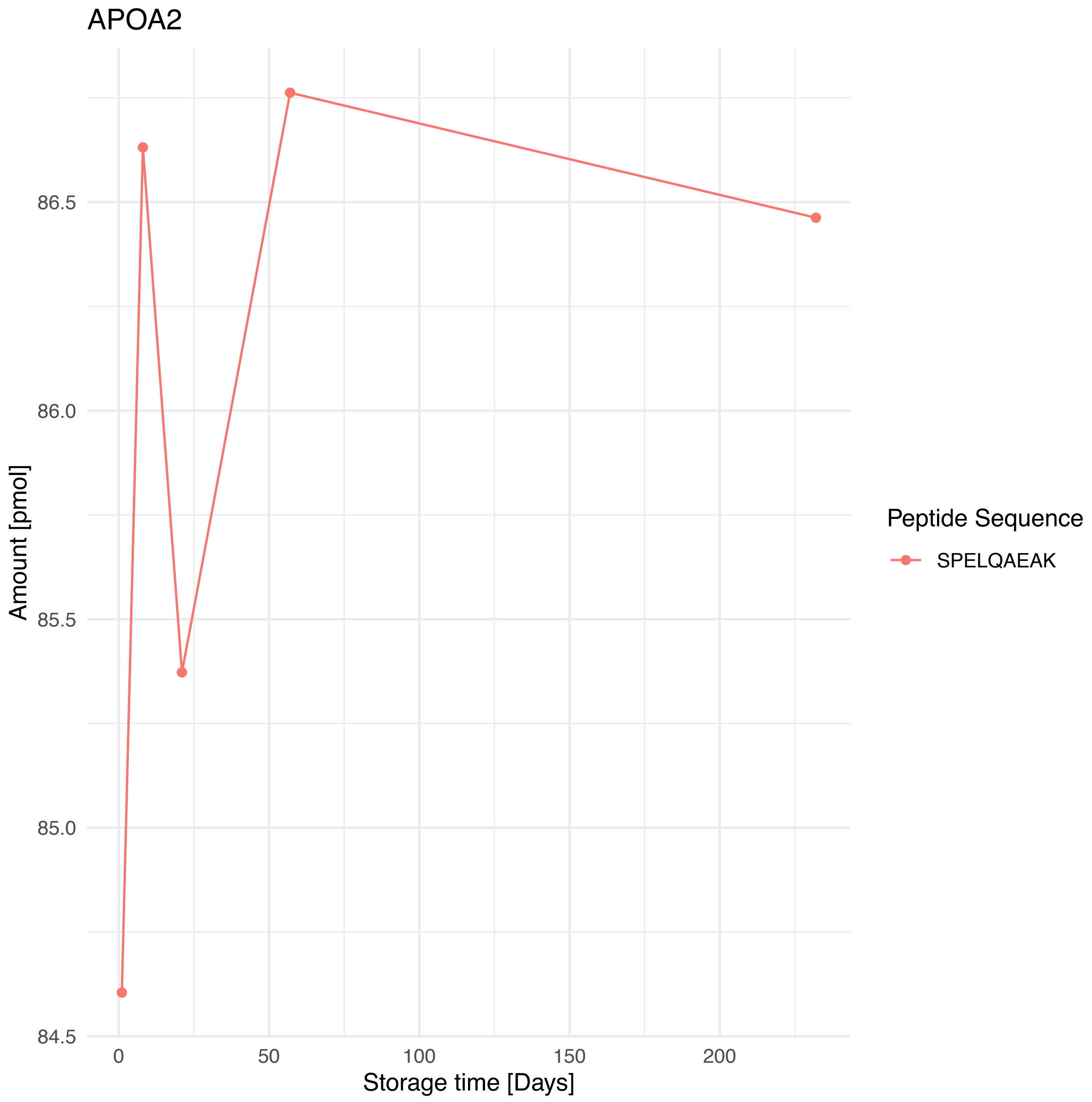
APOA2
Amount [pmol]
86.5
86.0
85.5
85.0
84.5
0
50
100
150
200
Storage time [Days]
Peptide Sequence
SPELQAEAK

Figure S6

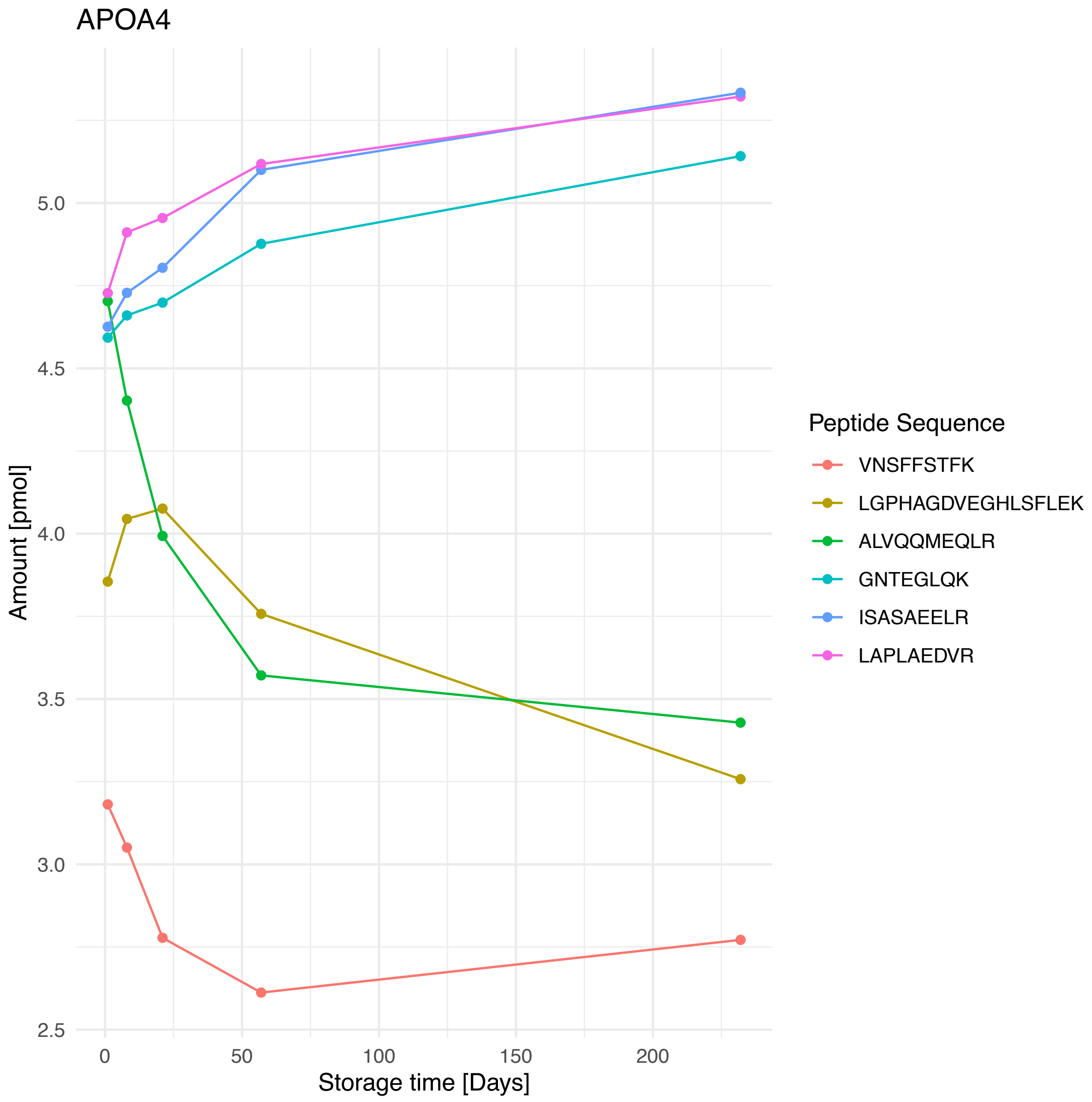
APOA4
5.0
4.5
4.0
3.5
3.0
2.5
Amount [pmol]
0
50
100
150
200
Storage time [Days]
Peptide Sequence
VNSFFSTFK
LGPHAGDVEGHLSFLEK
ALVQQMEQLR
GNTEGLQK
ISASAEELR
LAPLAEDVR

Figure S7

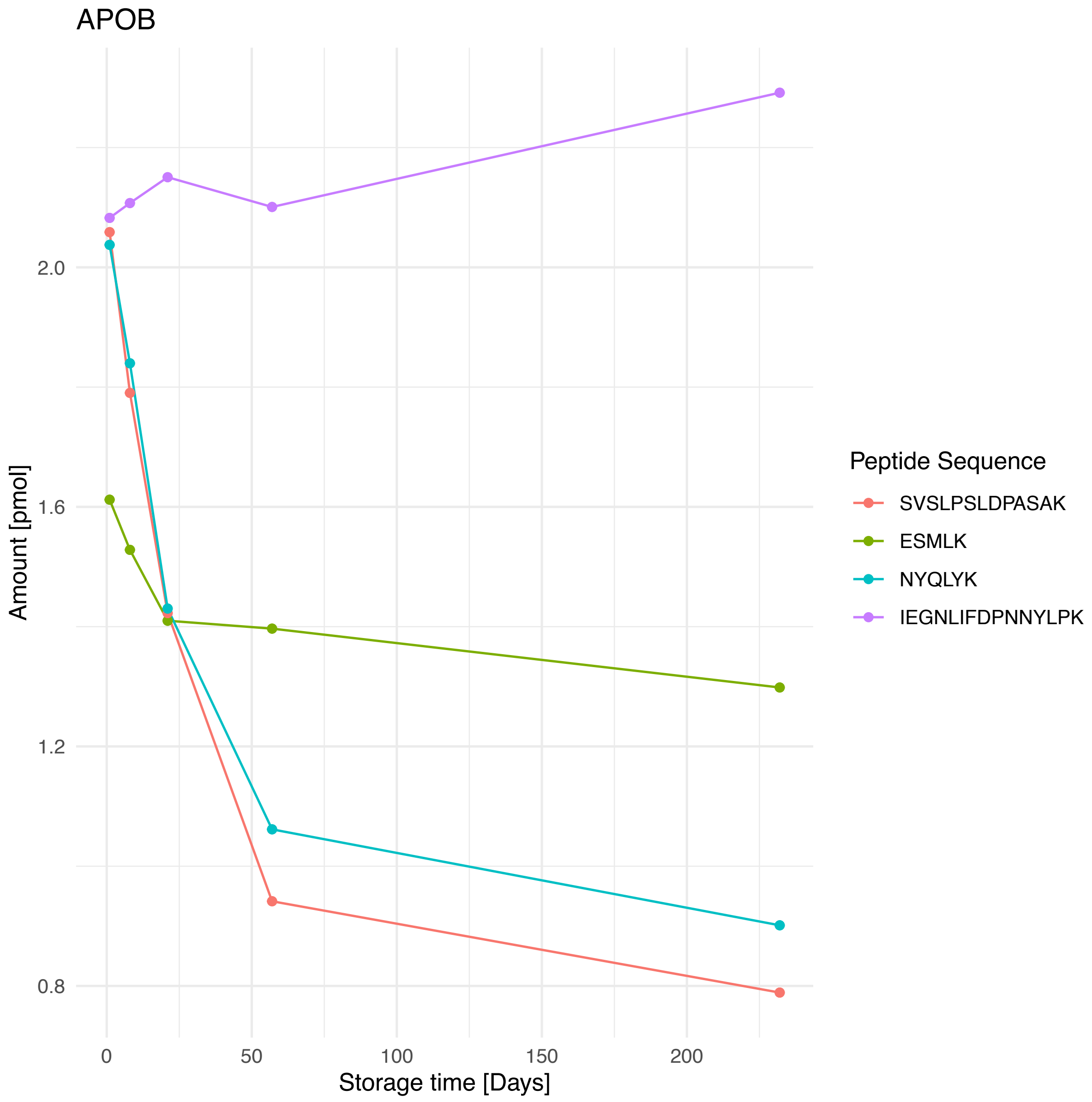

APOB
2.0
1.6
1.2
0.8
Amount [pmol]
0
50
100
150
200
Storage time [Days]
Peptide Sequence
SVSLPSLDPASAK
ESMLK
NYQLYK
IEGNLIFDPNNYLPK

Figure S8

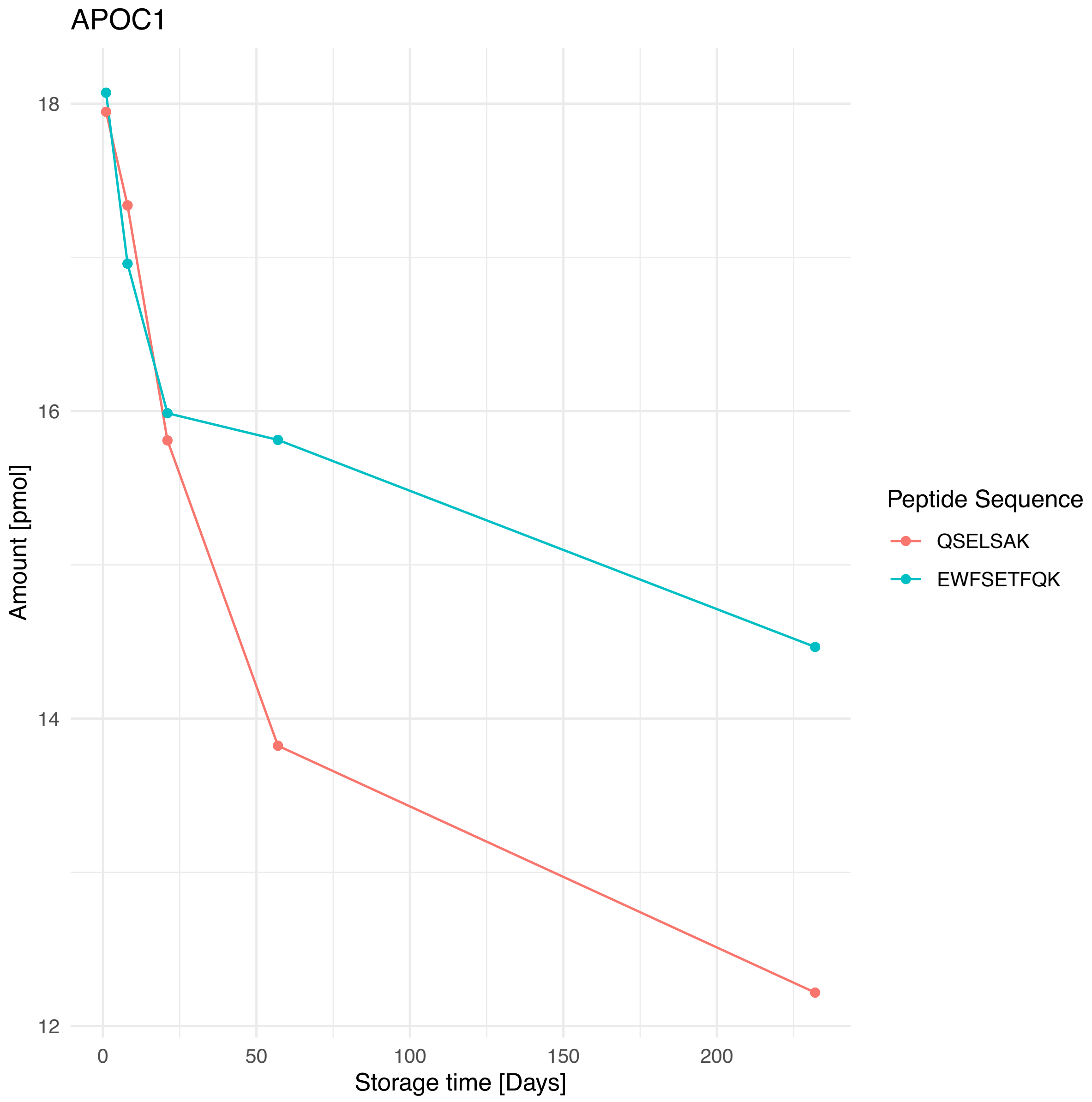

APOC1
Amount [pmol]
18
16
14
12
0
50
100
150
200
Storage time [Days]
Peptide Sequence
QSELSAK
EWFSETFQK

Figure S9

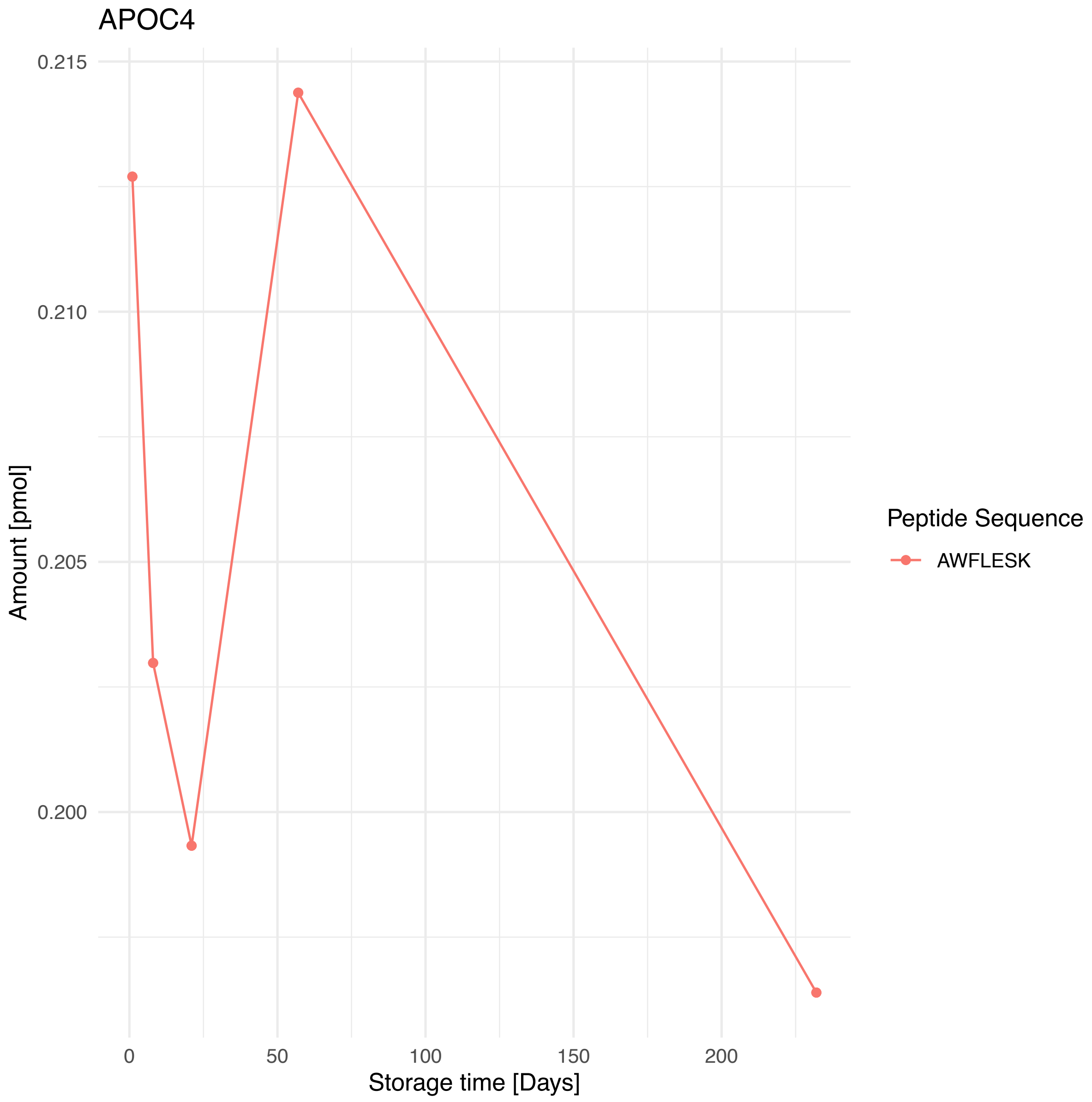

APOC4
0.215
0.210
0.205
0.200
Amount [pmol]
0
50
100
150
200
Storage time [Days]
Peptide Sequence
AWFLESK

Figure S10

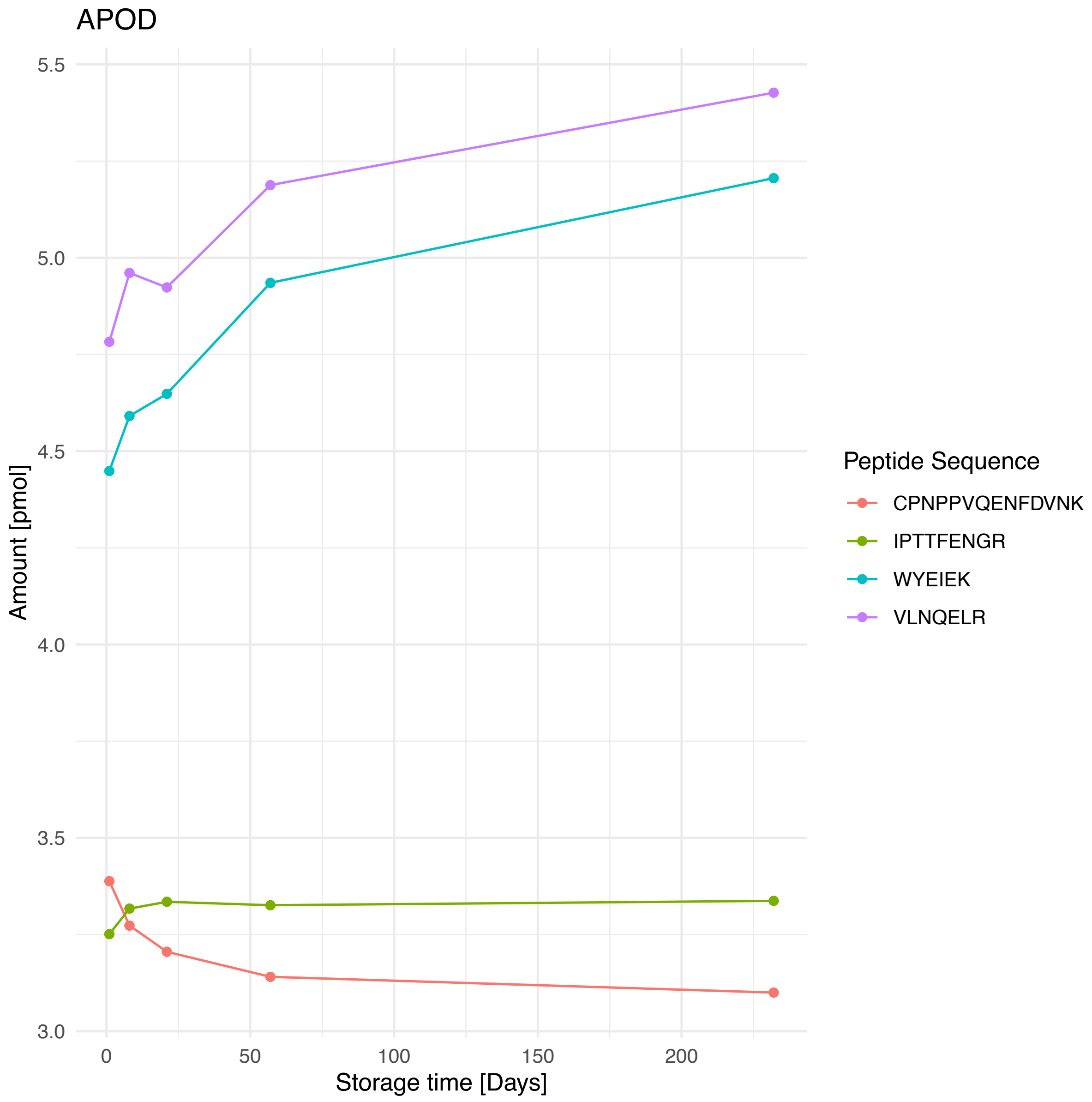
APOD
5.5
5.0
4.5
4.0
3.5
3.0
Amount [pmol]
0
50
100
150
200
Storage time [Days]
Peptide Sequence
CPNPPVQENFDVNK
IPTTFENGR
WYEIEK
VLNQELR

Figure S11

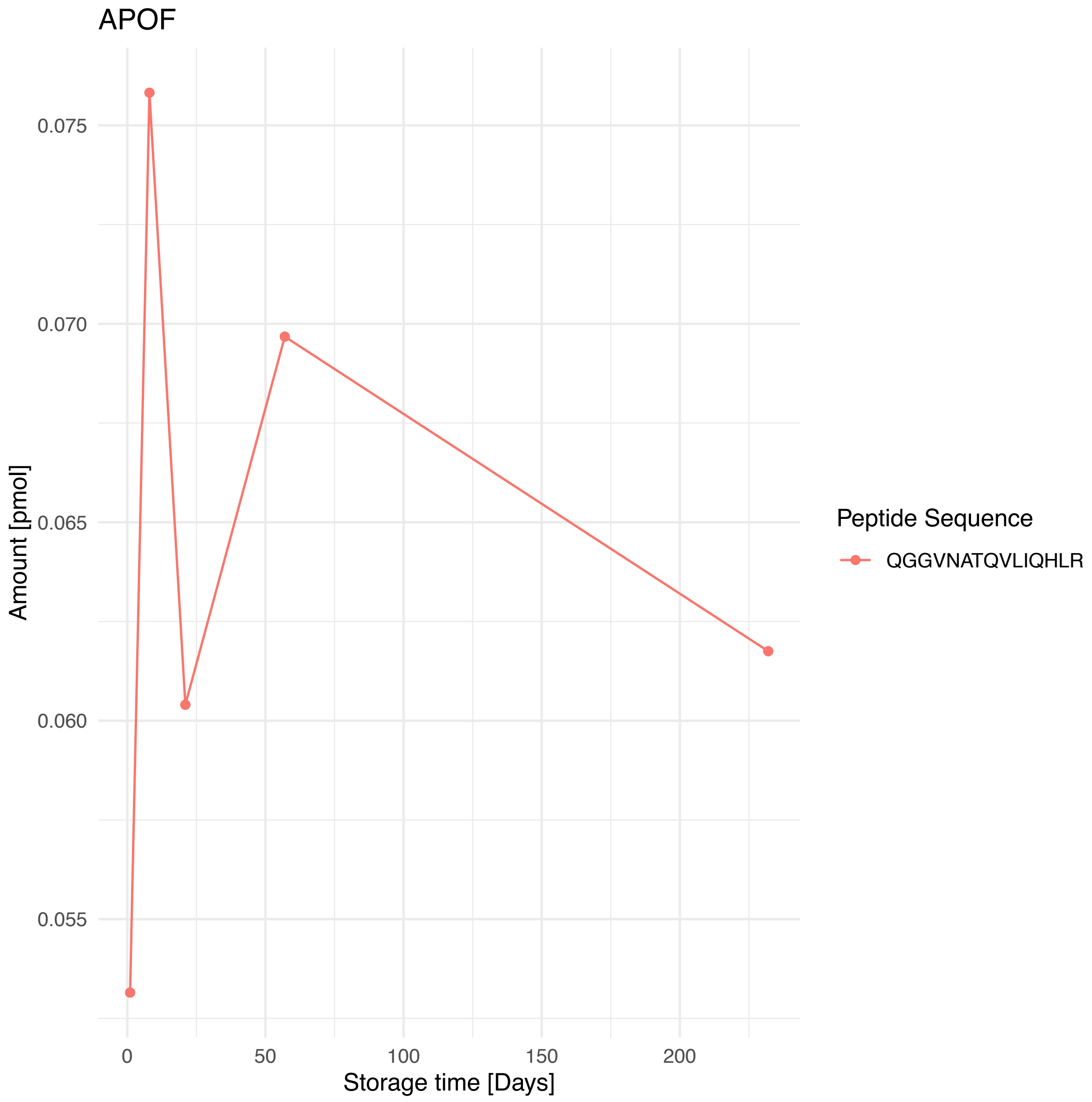
APOF
Amount [pmol]
0.075
0.070
0.065
0.060
0.055
0
50
100
150
200
Storage time [Days]
Peptide Sequence
QGGVNATQVLIQHLR

Figure S12

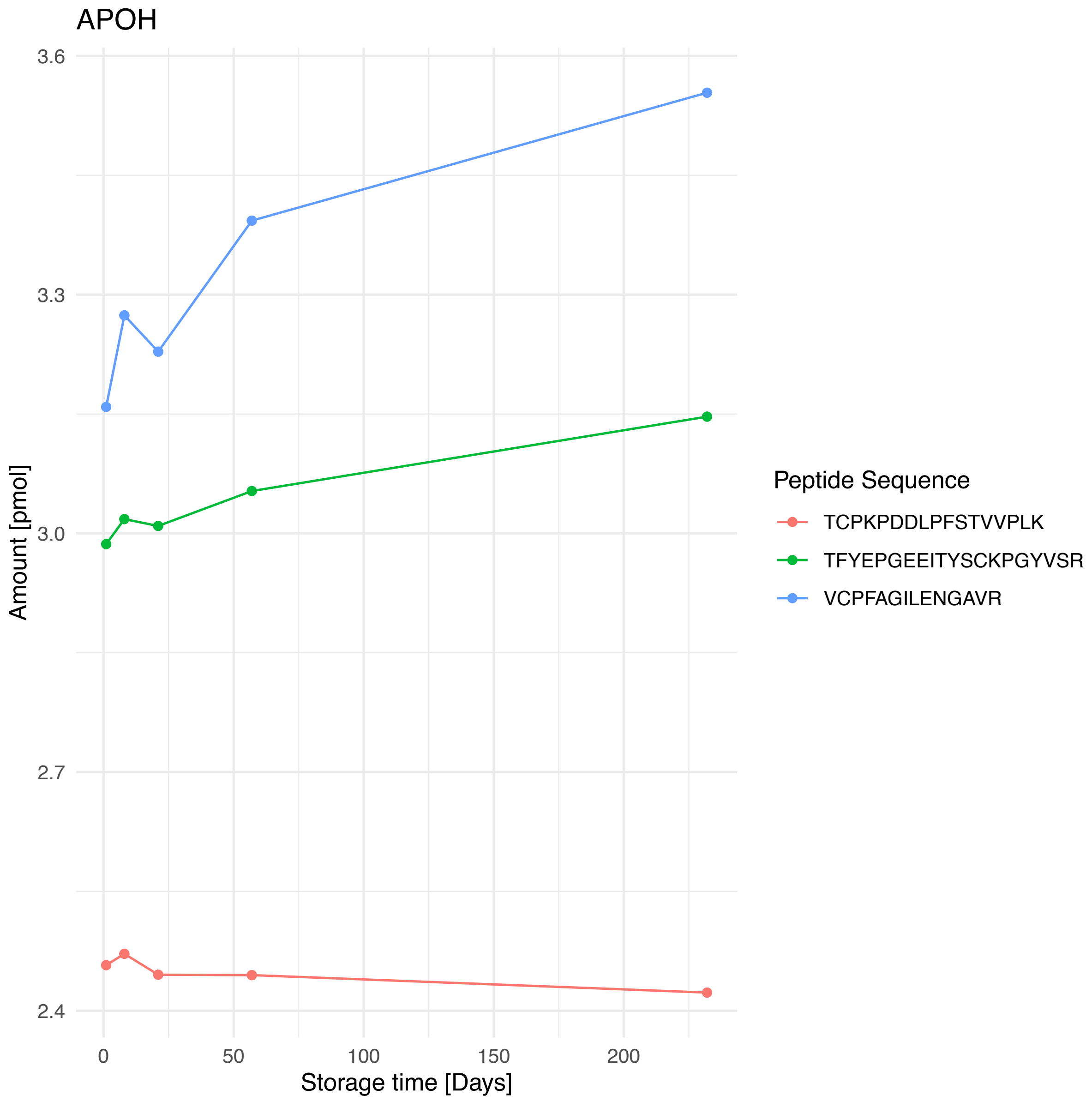
APOH
3.6
3.3
3.0
2.7
2.4
Amount [pmol]
0
50
100
150
200
Storage time [Days]
Peptide Sequence
TCPKPDDLPFSTVVPLK
TFYEPGEEITYSCKPGYVSR
VCPFAGILENGAVR

Figure S13

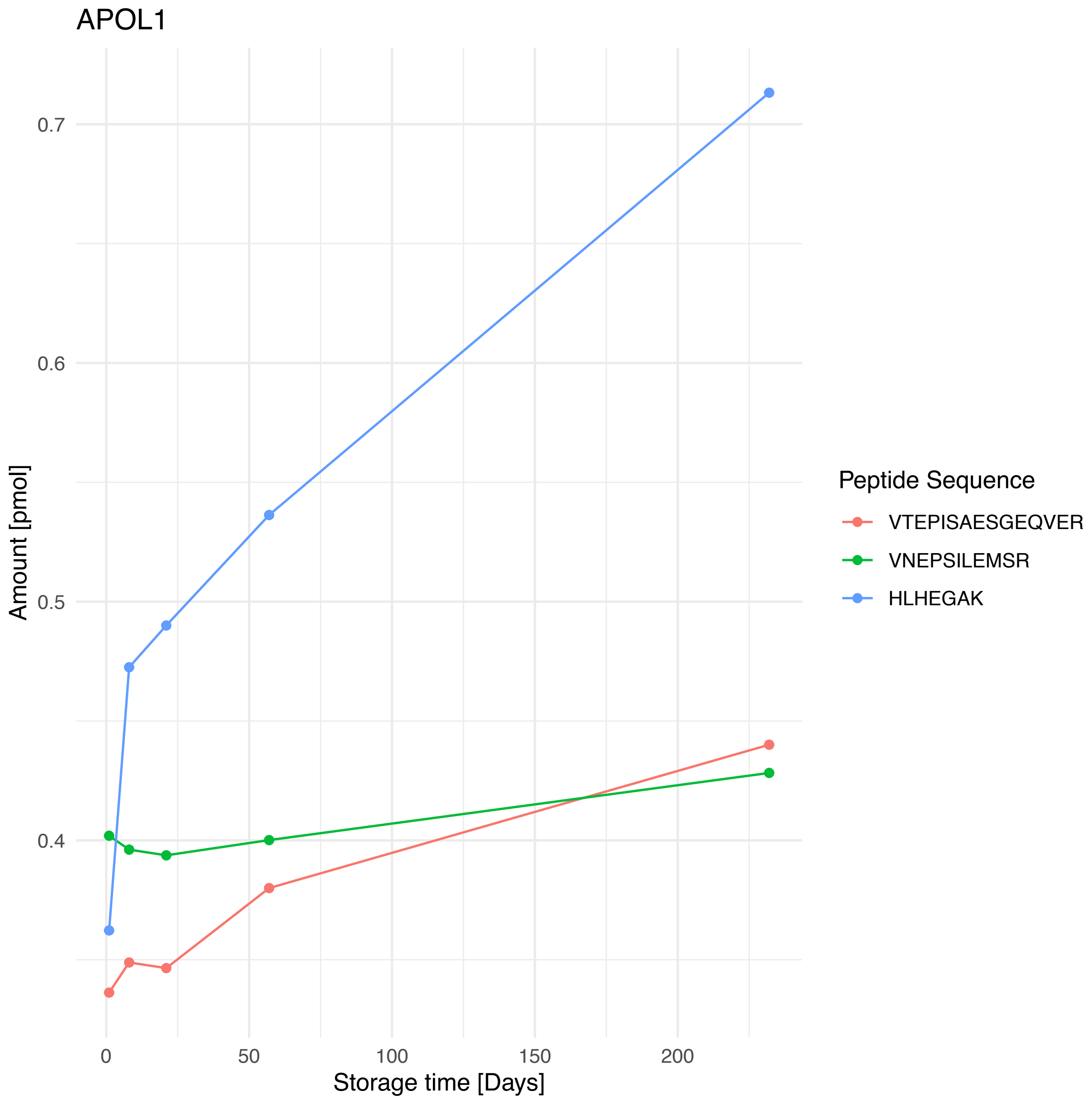
APOL1
Amount [pmol]
0.7
0.6
0.5
0.4
0
50
100
150
200
Storage time [Days]
Peptide Sequence
VTEPISAESGEQVER
VNEPSILEMSR
HLHEGAK

Figure S14

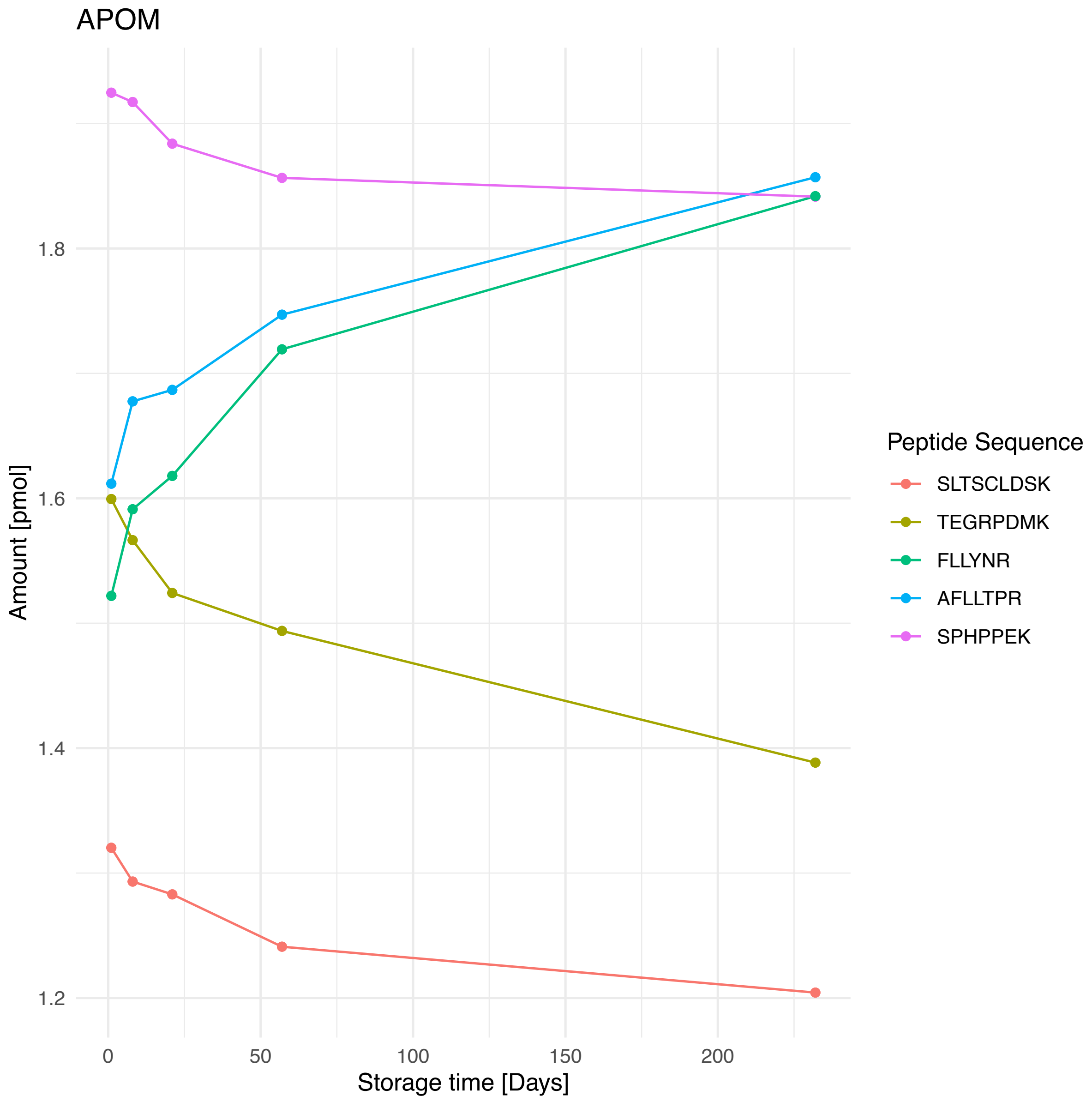

APOM
1.8
1.6
1.4
1.2
Amount [pmol]
0
50
100
150
200
Storage time [Days]
Peptide Sequence
SLTSCLDSK
TEGRPDMK
FLLYNR
AFLLTPR
SPHPPEK

Figure S15

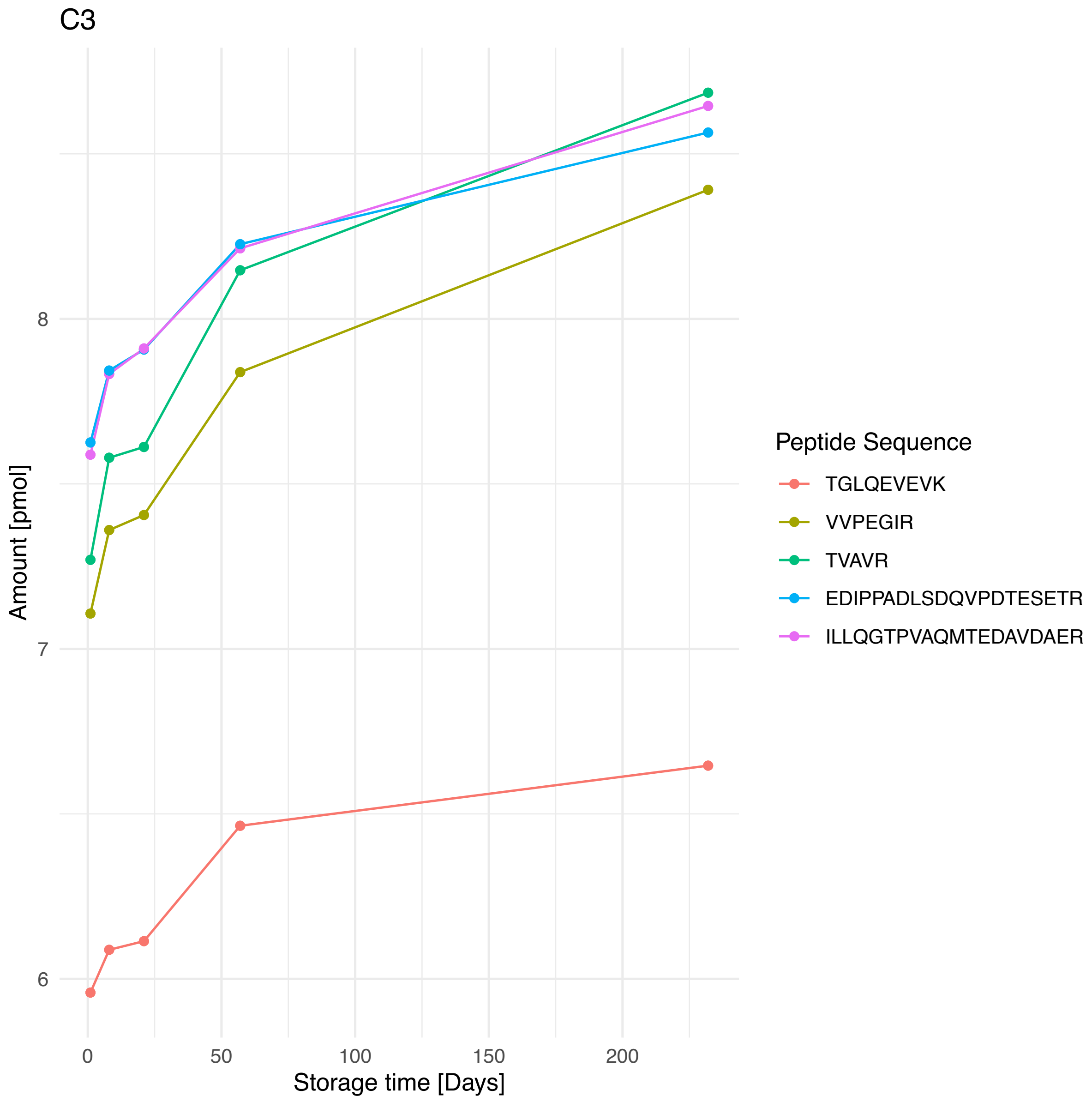

C3
Amount [pmol]
8
7
6
0
50
100
150
200
Storage time [Days]
Peptide Sequence
TGLQEVEVK
VVPEGIR
TVAVR
EDIPPADLSDQVPDTESETR
ILLQGTPVAQMTEDAVDAER

Figure S16

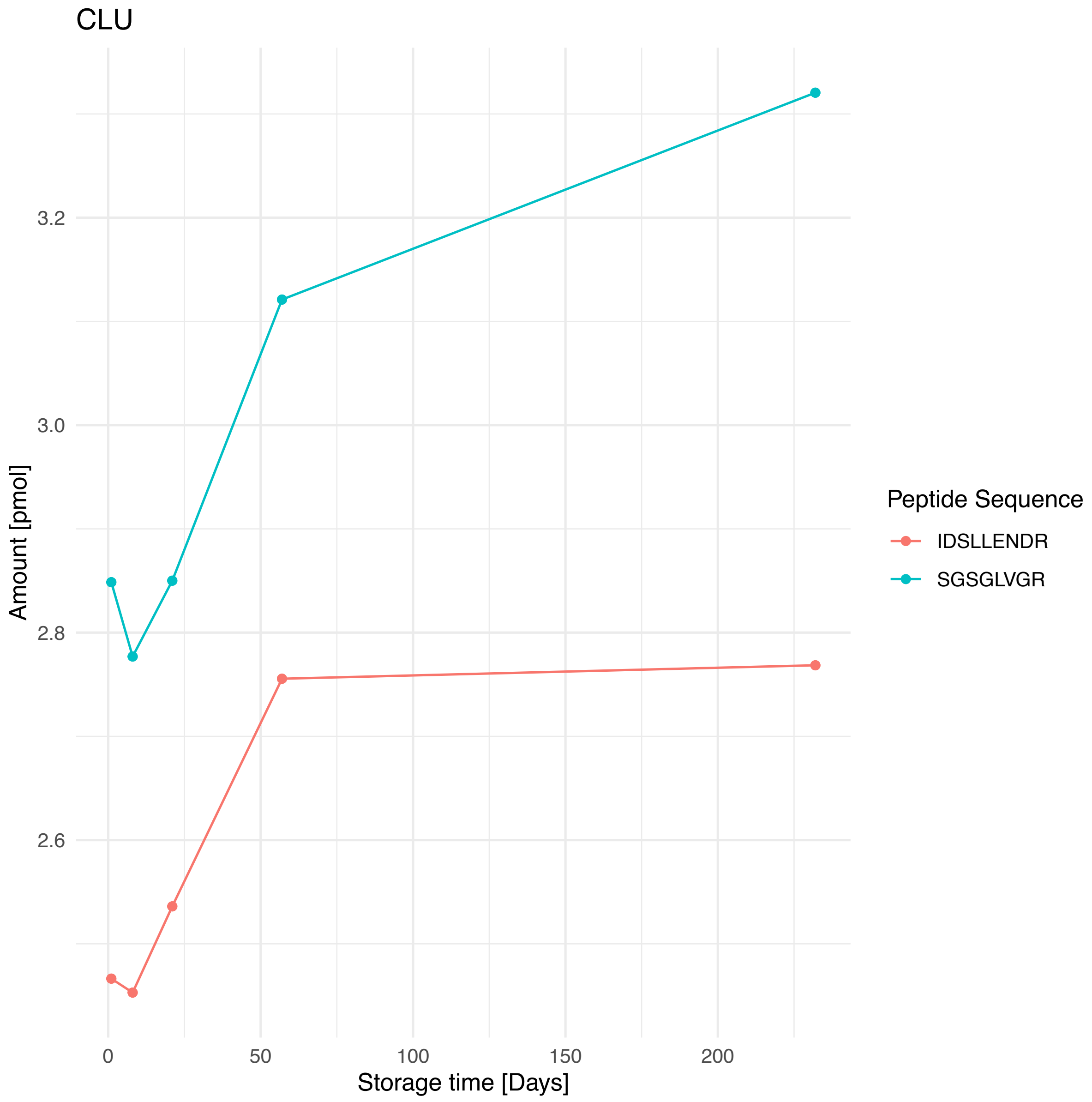
CLU
Amount [pmol]
3.2
3.0
2.8
2.6
0
50
100
150
200
Storage time [Days]
Peptide Sequence
IDSLLENDR
SGSGLVGR

Figure S17

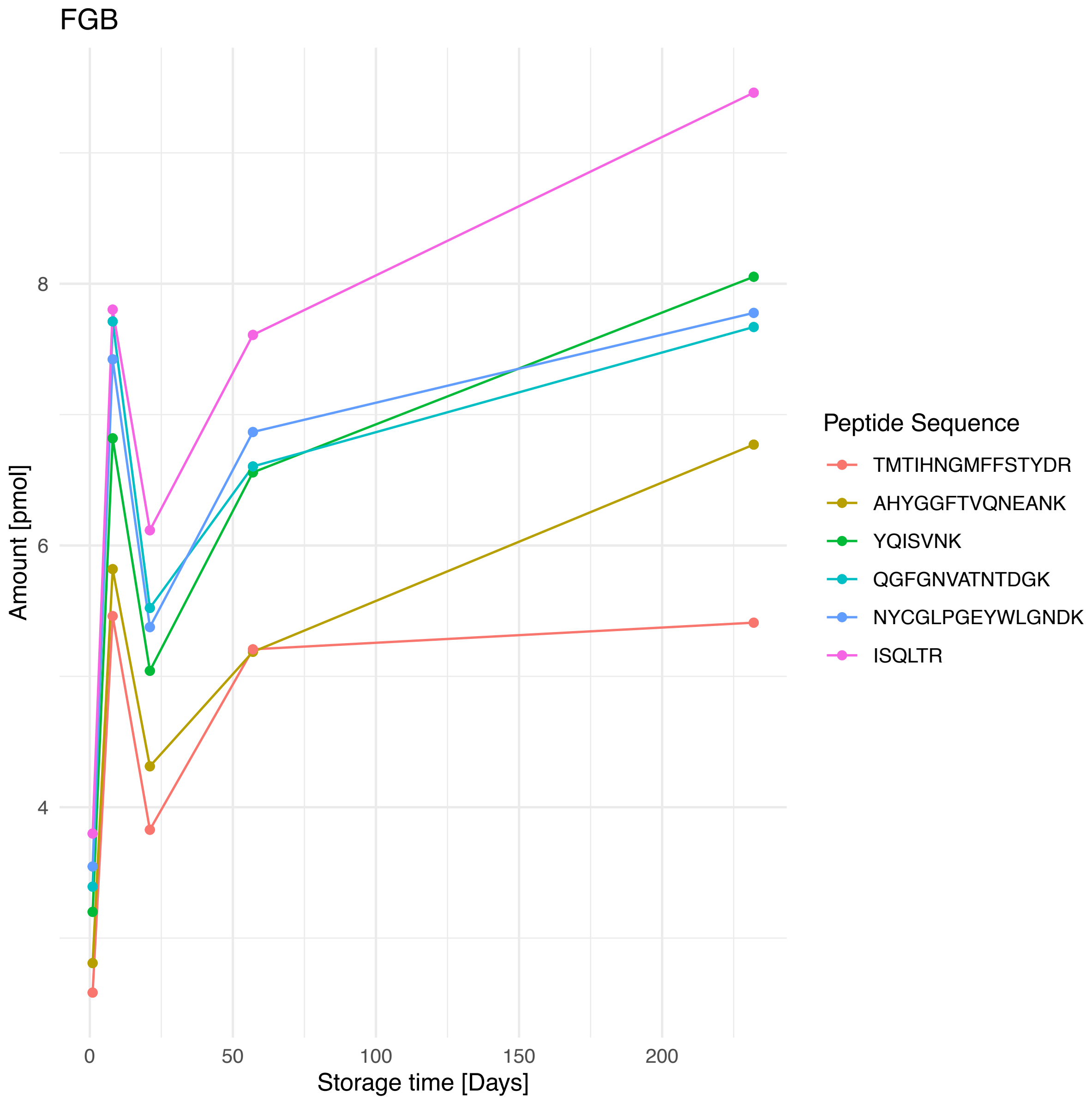
FGB
Amount [pmol]
4
6
8
0
50
100
150
200
Storage time [Days]
Peptide Sequence
TMTIHNGMFFSTYDR
AHYGGFTVQNEANK
YQISVNK
QGFGNVATNTDGK
NYCGLPGEYWLGNDK
ISQLTR

Figure S18

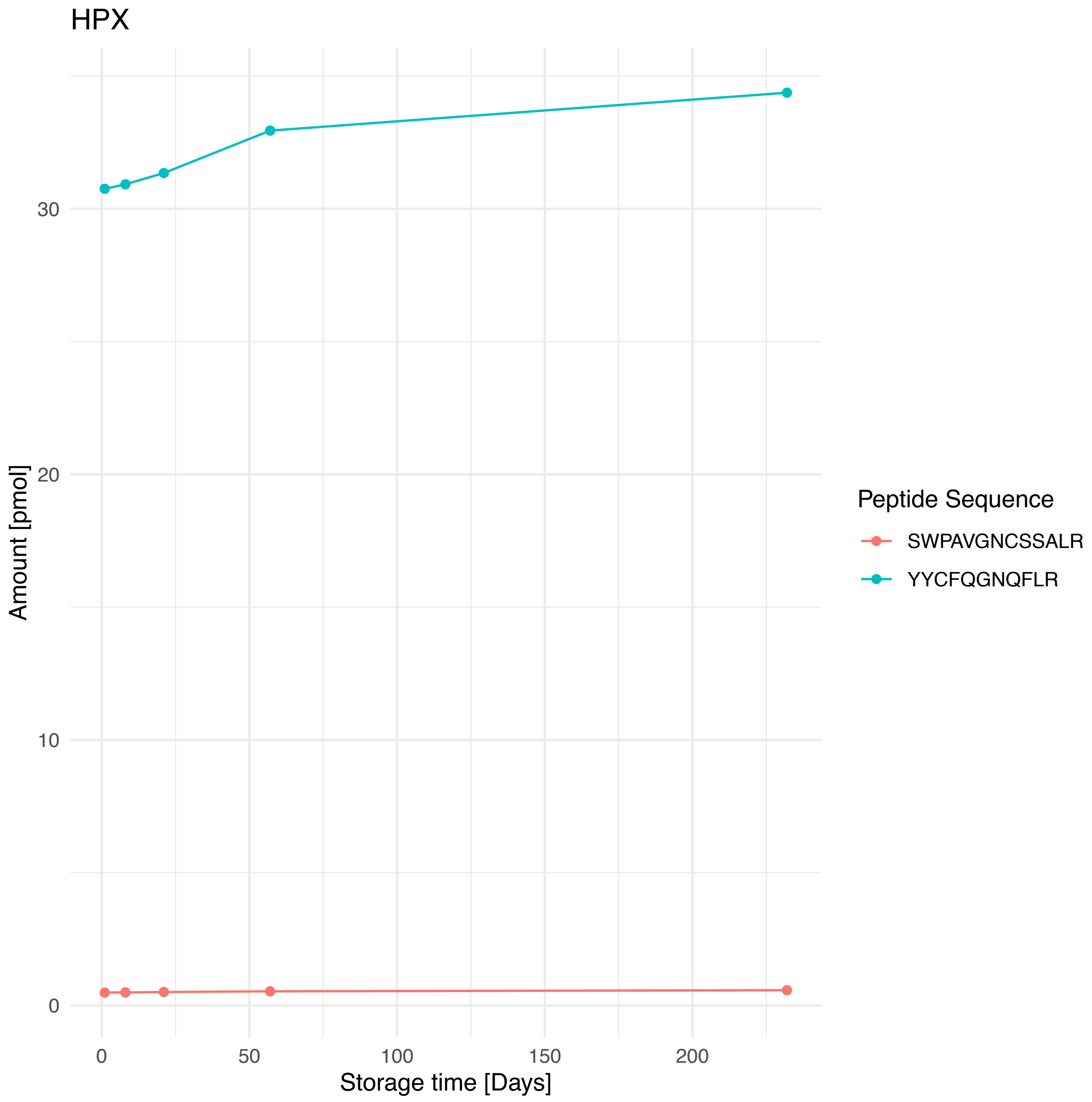
HPX
Amount [pmol]
0
10
20
30
0
50
100
150
200
Storage time [Days]
Peptide Sequence
SWPAVGNCSSALR
YYCFQGNQFLR

Figure S19

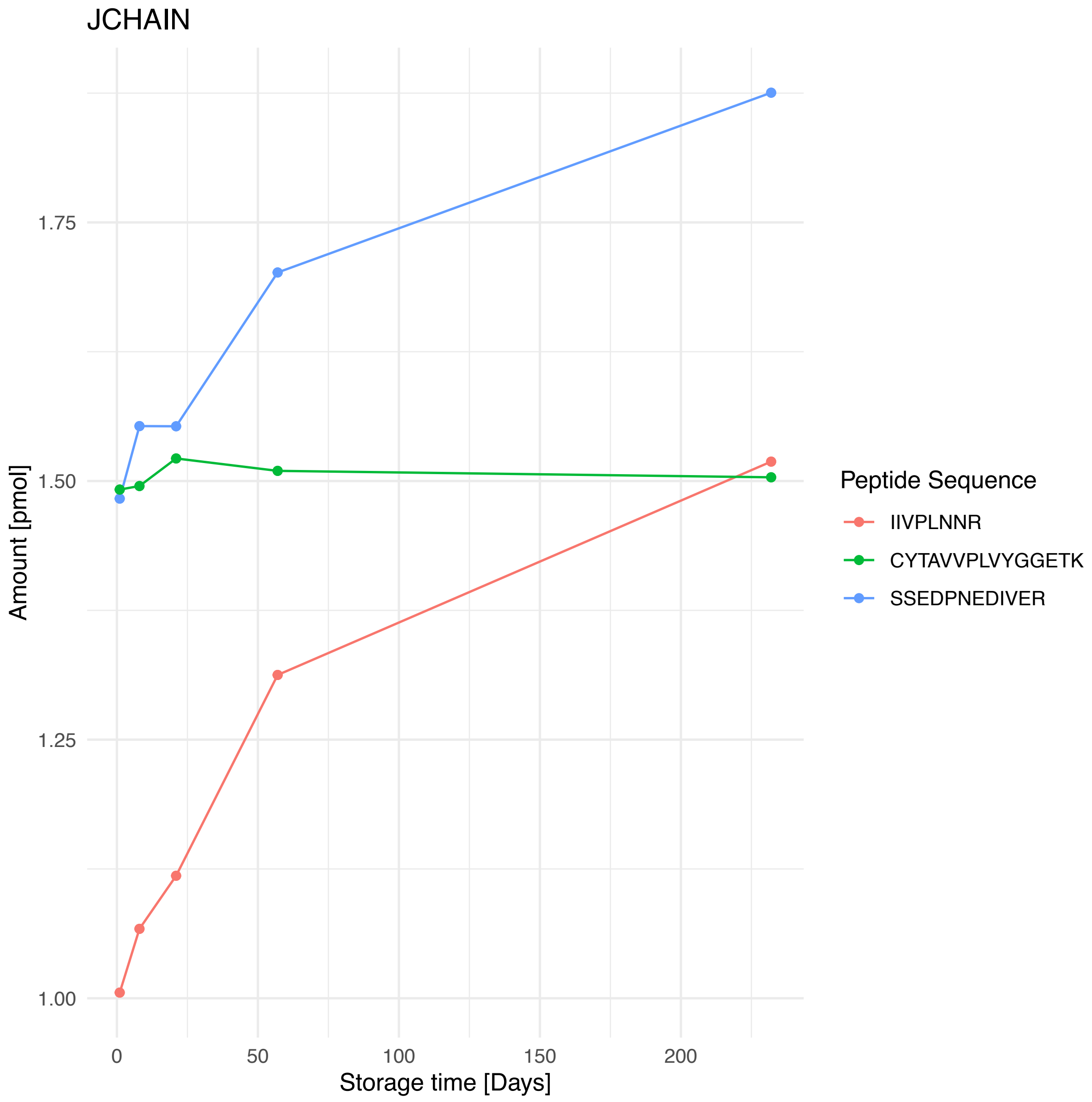
JCHAIN
Amount [pmol]
1.75
1.50
1.25
1.00
0
50
100
150
200
Storage time [Days]
Peptide Sequence
IIVPLNNR
CYTAVVPLVYGGETK
SSEDPNEDIVER

Figure S20

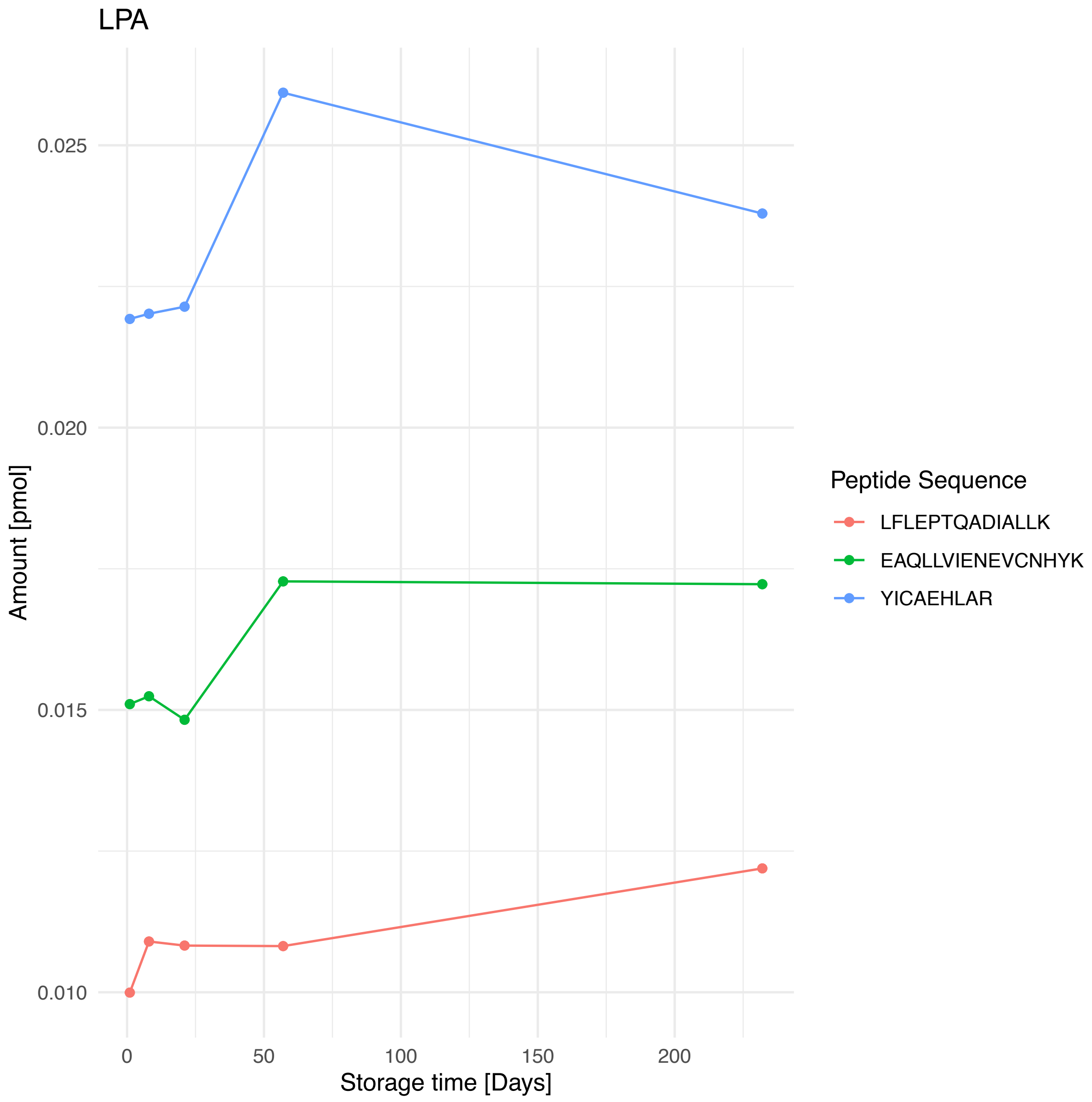
LPA
0.025
0.020
0.015
0.010
Amount [pmol]
0
50
100
150
200
Storage time [Days]
Peptide Sequence
LFLEPTQADIALLK
EAQLLVIENEVCNHYK
YICAEHLAR

Figure S21

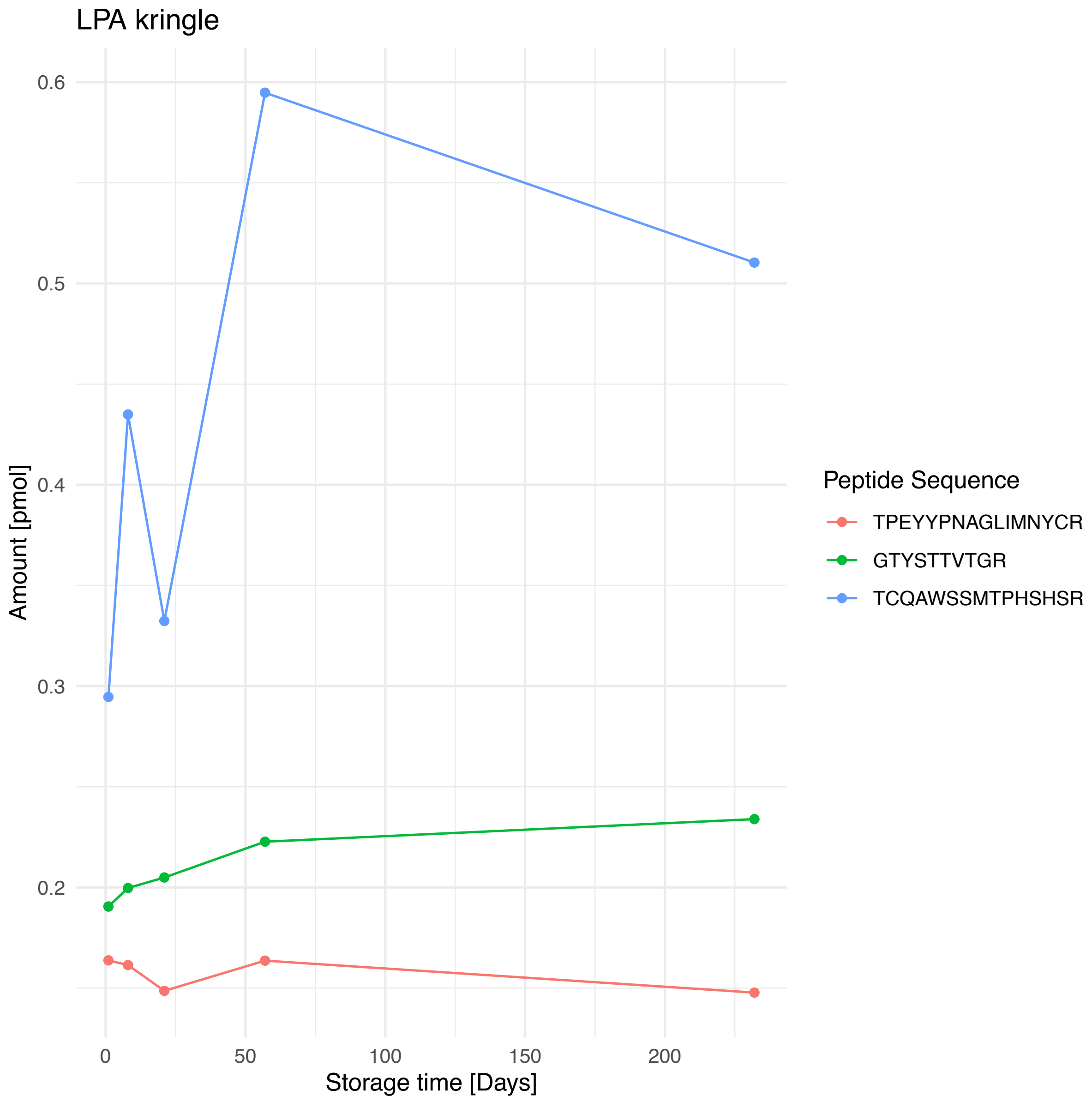
LPA kringle
Amount [pmol]
0.6
0.5
0.4
0.3
0.2
0
50
100
150
200
Storage time [Days]
Peptide Sequence
TPEYYPNAGLIMNYCR
GTYSTTVTGR
TCQAWSSMTPHSHSR

Figure S22

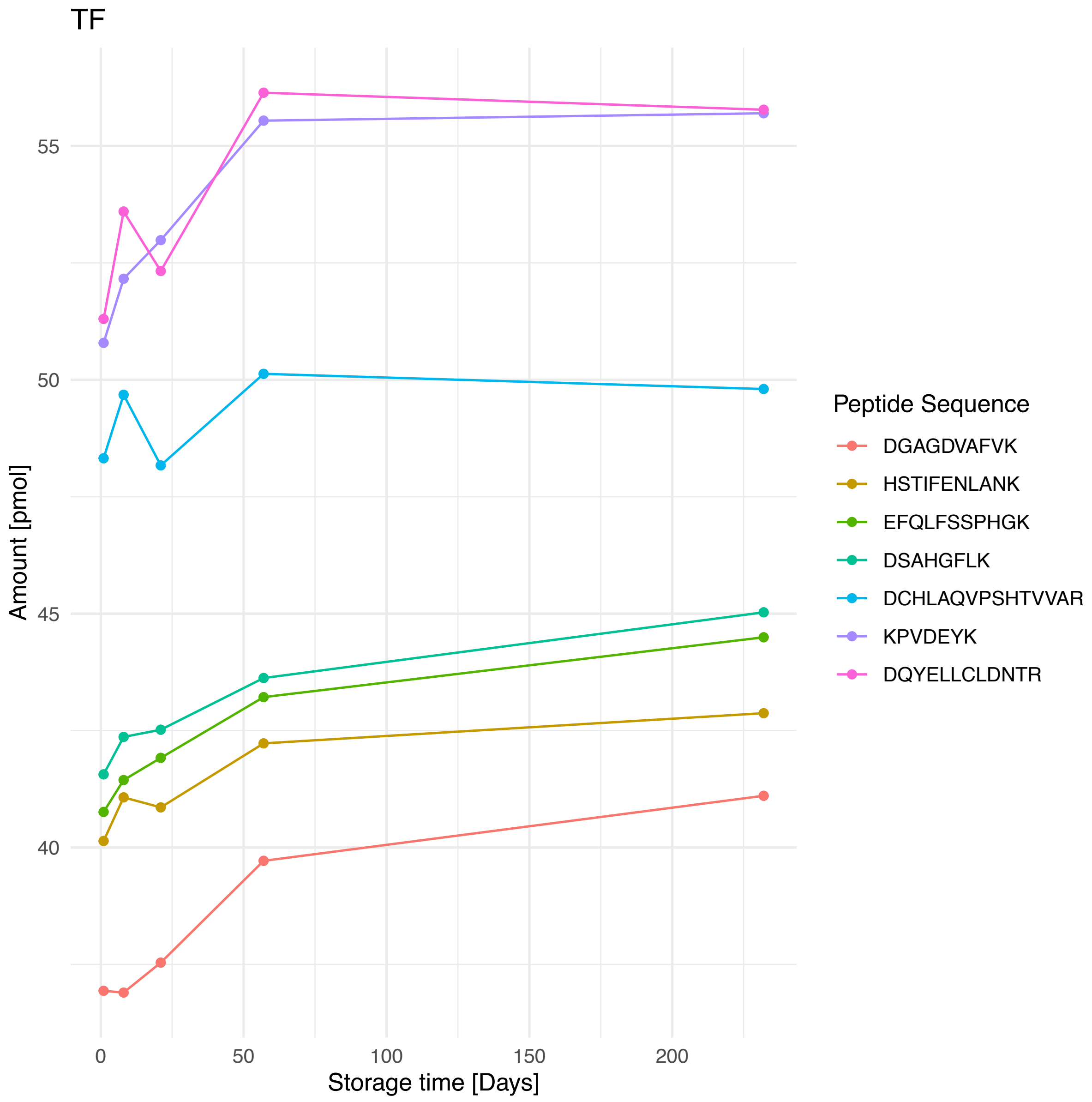

TF
55
50
45
40
0
50
100
150
200
Amount [pmol]
Storage time [Days]
Peptide Sequence
DGAGDVAFVK
HSTIFENLANK
EFQLFSSPHGK
DSAHGFLK
DCHLAQVPSHTVVAR
KPVDEYK
DQYELLCLDNTR
